# Supplementary material for: Association of microRNA-210-3p with NT-proBNP, sST2, and Galectin-3 in heart failure patients with preserved and reduced ejection fraction: A cross-sectional study
Source: PLoS One. 2025 Apr 3;20(4):e0320365. doi: 10.1371/journal.pone.0320365 (PMC11991677; doi:10.1371/journal.pone.0320365)
Supplement: Attachment — Submitted filename: PONE-D-24-15389_R1_reviewer (3).pdf [file pone.0320365.s004.pdf]

PLOS ONE

# Association of NT-proBNP, sST2, Galectin-3, and microRNA-210-3p in heart failure patients with preserved and reduced ejection fraction- cross-sectional study

--Manuscript Draft--

| | |
|---|---|
| Manuscript Number: | PONE-D-24-15389R1 |
| Article Type: | Research Article |
| Full Title: | Association of NT-proBNP, sST2, Galectin-3, and microRNA-210-3p in heart failure patients with preserved and reduced ejection fraction- cross-sectional study |
| Short Title: | NT-proBNP, sST2, Gal-3, Mir210-3p in HF |
| Corresponding Author: | Santhi Silambanan, MD, DNB<br>Sri Ramachandra Institute of Higher Education and Research (Deemed to be University)<br>Chennai, Tamil Nadu INDIA |
| Keywords: | Hear failure, ejection fraction, NT-proBNP, sST2, galectin-2, microRNA-210-3p |
| Abstract: | Background<br>Hear failure (HF) is a growing health problem and around two percentage are affected in the general population. The prevalence seems to be increasing due to improved management of communicable and noncommunicable diseases. Accurate diagnostic markers which have the potential in early diagnosis of HF is lacking. The aim of this study was to compare the levels of biomarkers -sST2, galectin-3 and microRNA-210-3p with the gold standard marker- NT-proBNP in heart failure patients with preserved and reduced ejection fractions.<br>Materials and Methods<br>The cross-sectional study was conducted in 270 hypertensive heart failure patients in the age group of 30 to 75 years of both genders. The participants with evidence of HF were recruited from the Department of Cardiology in a tertiary care hospital in Chennai, India. Blood samples were obtained for analysis of NT-proBNP, sST2, and galectin-3 were measured by ELISA. For microRNA-210-3p analysis, 20 apparently healthy individuals were included. MicroRNA was analyzed by qRT-PCR. Institutional ethics committee approval and written informed consent were obtained. Statistical analysis was performed using R software version 4.2.1. The obtained data were subjected to normality of distribution. Based on the type of distribution, appropriate statistical tools were used to analyze the obtained data. p value ≤ 0.05 was considered to be statistically significant.<br>Results<br>NT-proBNP, sST2, galectin-3 and microRNA-210-3p were significantly higher in HFrEF compared to that of HFpEF and showed correlation among the markers. MAGGIC score showed positive correlation with all the biomarkers. The cut-off of MAGGIC score was 14, miRNA-210-3p was s 5.03, NT-proBNP was 355.5 and sST2 was 20.11. However, galectin-3 appeared to have limited value, with an AUC of 0.51 and a non-significant p value of 0.60.<br>Conclusion<br>All the biomarkers were significantly elevated in HFrEF compared to HFpEF; hence, the biomarkers were able to differentiate the various HF cases. MicroRNA-210-3p could be an early marker in the diagnosis of heart failure. The strategy of employing multi-marker approach could help in the early diagnosis as well as in stratifying the HF patients. This could aid in implementing targeted therapies. |
| Order of Authors: | Jasmine Chandra A, PhD |
| | Mohanalakshmi P, MD |
| | Sudagar Singh RB, MD |
| | Mahesh Kumar K, PhD |
| | Karthikeyan Rajamani, PhD |
| | Santhi Silambanan, MD, DNB |

Powered by Editorial Manager® and ProduXion Manager® from Aries Systems Corporation

| | |
|---|---|
| **Response to Reviewers:** | The authors are grateful for all the suggestions raised by the reviewers and editors. Herewith we are attaching the responses to the queries. We have taken utmost care and responsibility in addressing the queries. Once again thank you very much.<br><br>Responses to Journal requirements:14-09-2024<br><br>1.Please ensure that your manuscript meets PLOS ONE's style requirements, including those for file naming.<br>Response: The manuscript has been modified according to the PLOS ONE's requirements.<br>2.When completing the data availability statement of the submission form, you indicated that you will make your data available on acceptance. We strongly recommend all authors decide on a data sharing plan before acceptance, as the process can be lengthy and hold up publication timelines. Please note that, though access restrictions are acceptable now, your entire data will need to be made freely accessible if your manuscript is accepted for publication. This policy applies to all data except where public deposition would breach compliance with the protocol approved by your research ethics board. If you are unable to adhere to our open data policy, please kindly revise your statement to explain your reasoning and we will seek the editor's input on an exemption. Please be assured that, once you have provided your new statement, the assessment of your exemption will not hold up the peer review process.<br>Response: The authors confirm that the data supporting the findings of this study are available within the article. Raw data were generated at the location of research, Sri Ramachandra Institute of Higher Education and Research, Chennai, India. No supplementary material is available with this research.<br>3.Please remove your figures from within your manuscript file, leaving only the individual TIFF/EPS image files, uploaded separately. These will be automatically included in the reviewers' PDF.<br>Responses: Figures are removed from the manuscript file and uploaded separately as TIFF files.<br><br>Response to the reviewers comments:<br>1. Is the manuscript technically sound, and do the data support the conclusions?<br><br>The manuscript must describe a technically sound piece of scientific research with data that supports the conclusions. Experiments must have been conducted rigorously, with appropriate controls, replication, and sample sizes. The conclusions must be drawn appropriately based on the data presented.<br>Reviewer #1: Yes<br>Reviewer #2: Partly<br>Response: The manuscript has been edited with the responses to the reviewers, which we think could improve the content and presentation of the manuscript.<br> 2. Has the statistical analysis been performed appropriately and rigorously?<br>Reviewer #1: Yes<br>Reviewer #2: No<br>Response: Correlation studies and ROC curve have been modified. The information is added in the methodology, results, discussion and conclusion.<br> 3. Have the authors made all data underlying the findings in their manuscript fully available?<br><br>The PLOS Data policy requires authors to make all data underlying the findings described in their manuscript fully available without restriction, with rare exception (please refer to the Data Availability Statement in the manuscript PDF file). The data should be provided as part of the manuscript or its supporting information, or deposited to a public repository. For example, in addition to summary statistics, the data points behind means, medians and variance measures should be available. If there are restrictions on publicly sharing data—e.g. participant privacy or use of data from a third party—those must be specified.<br>Reviewer #1: Yes<br>Reviewer #2: Yes<br>4. Is the manuscript presented in an intelligible fashion and written in standard English? |

Powered by Editorial Manager® and ProduXion Manager® from Aries Systems Corporation

PLOS ONE does not copyedit accepted manuscripts, so the language in submitted articles must be clear, correct, and unambiguous. Any typographical or grammatical errors should be corrected at revision, so please note any specific errors here.
Reviewer #1: Yes
Reviewer #2: Yes
5. Review Comments to the Author

Please use the space provided to explain your answers to the questions above. You may also include additional comments for the author, including concerns about dual publication, research ethics, or publication ethics. (Please upload your review as an attachment if it exceeds 20,000 characters)
Reviewer #1: The authors reported that NT-proBNP, sST2 and galectin-3 were significantly higher in HFrEF compared to that of HFpEF. Also, microRNA-210-3p showed significantly increased expression in HFpEF compared to controls. The findings seem to be interesting, however, I would like to make some comments.

1.Major concerns.
- The lack of the novelty is the concern that I would like to express around the article, while data for miR-210-3p deserve to be published.
Response: The study was intended to be conducted since NT-proBNP levels do not correlate with the severity of disease especially in cases of HFpEF. Hence, we researched to find out whether sST2 and Gal-3 could be able to diagnose those cases which were not identified by NT-proBNP. Also, microRNAs could serve as very early biomarkers of the disease. Like we do profile of certain biomarkers in case of liver or renal diseases, a profile of biomarkers in HF will reduce the chances of morbidity and mortality. Of late women tend to develop hypertension almost similar to that of men. Studies show that women respond poorly to antihypertensives compared to men. Hence, the chances of increasing cases of HFpEF is high in future. Keeping all these in mind, we proceeded with the study.

- The authors did not validate the findings and the comparisons with widely used scores (MAGGIC, Barcelona-HF, etc.) were not performed
Methodology: The Meta-Analysis Global Group in Chronic Heart Failure (MAGGIC) mortality risk score was calculated in all the study participants. The MAGGIC score consists of thirteen clinical predictor variables such as age, sex, body mass index (BMI), systolic blood pressure (SBP), ejection fraction (EF), serum creatinine level, participant is a current smoker, has diabetes mellitus, and chronic obstructive pulmonary disease, belongs to New York Heart Association (NYHA) class, heart failure (HF) diagnosed for at least eighteen months, use of beta-blockers and angiotensin-converting enzyme (ACE) inhibitors.

2. Minor concerns
- Structure of the paper is not logic. Section Results contains a paragraph for B-mode Echo, while it could replaced to the subsection Methods. Please, check and correct.
Response: The following removed in results and added in methodology
The study participants were subjected to the transthoracic 2D doppler echocardiography using Phillips and GE Healthcare echocardiography with patients lying supine in the left lateral decubitus position. EF values in healthy individuals and in heart failure patients vary with ethnicity and type of ECHO instrument. According to the definition of European and United States guidelines, the normal EF is 52-72% in men and 54-74% in women [21]. According to Chengode, EF > 55% is considered to be normal. It can be 45%–54%, 30%–44% and < 30% in mild, moderate and severe LV dysfunction respectively [22].
Anthropometric characteristics such as body mass index (BMI) in Kg/m2 and waist to hip ratio (WHR) were measured as per the Asia-Pacific guidelines [23]. Systolic and diastolic blood pressures were measured using standard procedure and patients were identified as hypertensives based on the JNC7 blood pressure classification [24].
According to International Expert Committee, diabetes mellitus was diagnosed based on glycated hemoglobin (HbA1c) ≥6.5%, fasting plasma glucose ≥126 mg/dL and 2-hour plasma glucose or random plasma glucose ≥200 mg/dL [25].
LV ejection fraction (EF) remains the major parameter for phenotyping, diagnosis, prognosis and treatment decisions in HF. In the present study, EF of 50% was used as the cut-off to categorize the participants into two groups as group 1 – HF with EF ≤ 49% and group 2 -HF with EF 50%. As per the 2016- ESC Guidelines, EF of 40- 49%

Powered by Editorial Manager® and ProduXion Manager® from Aries Systems Corporation

is categorized as HFmrEF. HFmrEF is an intermediate clinical entity between HFrEF and HFpEF, but more similar to HFrEF with regard to the high prevalence of AMI in these patients [7]. Patients with HFmrEF are similar to HFrEF in being younger males, increased systolic blood pressure and reduced LV and atrial dilatation. Also, HFmrEF are found to benefit from HFrEF treatment strategies [7]. In the present study, based on the above literature data, HFmrEF has been included in HFrEF.

- The authors did not report inclusion / exclusion criteria, flow chart for patients enrolment, comorbidity status, anthropometric findings. Table 1 does not include the parameters mentioned above.
Response: Figure 1 shows the flow chart indicating the recruitment of participants into the study along with patient characteristics. Table 1 shows the demographic data of the study participants.

- Biomarkers evaluation and blood sampling. It remains unclear now and in which ways the sampling were performed.
Response: The heart failure patients who were attending cardiology department were recruited. Based on EF as shown by ECHO they were categorized as group I or 2. Subsequently blood samples were drawn for analysis of biomarkers and microRNA. Serum and plasma were separated and stored at -80C until analysis.

- Did the authors discover other correlations between biomarkers and comorbidities, metabolic status, i.e. fasting glucose, HOMA-IR, lipid profile.
Response: The correlations of cardiac biomarkers with lipids, MAGGIC score and HbA1c are shown in table 6

Reviewer #2: With the aim of investigating levels of biomarkers in HF and to compare between HFrEF and HFpEF, the authors performed a cross-sectional study enrolling 270 patients. The authors are to be commended for the great effort. However, there are some points in need of further clarification.

Thank you very much for analyzing our manuscript critically. I am grateful for the valuable suggestions and giving us the opportunity to answer to the queries. Indeed, we are thankful and most obliged for all the constructive comments. Thank you

Specific points:

-Abstract:
Too much information about methods; please shorten it
Suddenly, the authors named in the result section controls. Please, add in the method.
Responses: Abstract -methodology has been modified. The information about controls has been added in the methodology

-Introduction: too long. Please, go straight to the main focus of the paper.
Response: Introduction was edited and excess information has been removed.

-Methods: to include only Hypertensive patients with HF and then add patients with DM could be considered as a mistake.
Response: Initially the study was decided to be conducted in hypertensive patients only, since hypertension has not been studied in Indian population. Due to the sudden onset of COVID-19 pandemic, patient visit to the hospital was limited. Hence, with the discussion and approval of research advisory committee members, it was decided to include hypertensives who also had diabetes mellitus. Ethics approval was sought again for the change in the criteria of recruitment of patients.

-Results: the result section should include only results. Please, delete all the information that are not results.
Results section has been modified and only results have been included. Information related to methodology have been added in methods section.

Please, add also comparison with regard to sex (M vs F) and age.
Response: Tables 3 and 4 have been added to compare the groups according to gender and age respectively.

Powered by Editorial Manager® and ProduXion Manager® from Aries Systems Corporation

| | |
|---|---|
| | -Discussion:<br>Too long and not focused on the main results. Pleas shorten it and make more focused on the main topic; in addition, add subparagraphs to improve readability.<br>Response: Discussion has been edited and shortened as per the suggestions.<br><br>To date, the role of biomarkers, and in particular the role of multimarkers, in HF can be considered in different stage (e.g., diagnosis, prognosis, management). Please comment using references (i.e., PMID 33673947 and 30832821)<br>Response: I had the opportunity to go through both the manuscripts. Both are really good, with good presentation and lot of information, pointing to almost the same findings which I have put forth in the manuscript. I have them in manuscript also.<br><br>Figures:<br>the figure are very low quality. Please increase.<br>Figure 1: please, miRNA and sST2 are too similar in color. Please change.<br>Response: The color has been changed. New figure with better clarity is attached<br><br>Consider to add in the text and in the discussion that one of the strengths of the present paper is that it has been performed in India (adding interesting additional data of patients whose ethnicity is classically not well investigated).<br>Response: Strength of the study has been added.<br>The study is carried out in India, where the resource settings are limited. India as such is highly diverse in economy, food, physical habits, literacy rate across the various states. But the study was carried out in a tertiary care hospital in a Metropolitan city. So to major extent it takes into account of the various factors. A multi-marker study in a relatively large sample size comprising of both genders remains to be major strength of the study.<br><br><br>Additional comment:<br>If the authors used AI for the preparation of this manuscript, please declare.<br>Response: The authors declare that AI for not used in preparation of this manuscript.<br>6. PLOS authors have the option to publish the peer review history of their article (what does this mean?). If published, this will include your full peer review and any attached files.<br><br>If you choose "no", your identity will remain anonymous but your review may still be made public.<br><br>Do you want your identity to be public for this peer review? For information about this choice, including consent withdrawal, please see our Privacy Policy.<br>Reviewer #1: No<br>Reviewer #2: Yes: Dr Andrea Salzano, MD, PhD |
| **Additional Information:** | |
| **Question** | **Response** |
| **Financial Disclosure**<br><br>Enter a financial disclosure statement that describes the sources of funding for the work included in this submission. Review the submission guidelines for detailed requirements. View published research articles from *PLOS ONE* for specific examples.<br><br><br>This statement is required for submission and **will appear in the published article** if the submission is accepted. Please make | Yes |

| | |
|---|---|
| sure it is accurate.<br><br>**Funded studies**<br>Enter a statement with the following details:<br>• Initials of the authors who received each award<br>• Grant numbers awarded to each author<br>• The full name of each funder<br>• URL of each funder website<br>• Did the sponsors or funders play any role in the study design, data collection and analysis, decision to publish, or preparation of the manuscript?<br><br>Did you receive funding for this work? | |
| Please add funding details.<br>as follow-up to "**Financial Disclosure**<br><br>Enter a financial disclosure statement that describes the sources of funding for the work included in this submission. Review the submission guidelines for detailed requirements. View published research articles from *PLOS ONE* for specific examples.<br><br>This statement is required for submission and **will appear in the published article** if the submission is accepted. Please make sure it is accurate.<br><br>**Funded studies**<br>Enter a statement with the following details:<br>• Initials of the authors who received each award<br>• Grant numbers awarded to each author<br>• The full name of each funder<br>• URL of each funder website<br>• Did the sponsors or funders play any role in the study design, data collection and analysis, decision to publish, or preparation of the manuscript?<br><br>Did you receive funding for this work?" | By JCA<br>U02B180535<br>From Sri Ramachandra University<br>Funding sources help in data collection and analysis |
| Please select the country of your main research funder (please select carefully | INDIA - IN |

| | |
|---|---|
| as in some cases this is used in fee calculation).<br>as follow-up to "**Financial Disclosure**<br><br>Enter a financial disclosure statement that describes the sources of funding for the work included in this submission. Review the submission guidelines for detailed requirements. View published research articles from *PLOS ONE* for specific examples.<br><br>This statement is required for submission and **will appear in the published article** if the submission is accepted. Please make sure it is accurate.<br><br>**Funded studies**<br>Enter a statement with the following details:<br>• Initials of the authors who received each award<br>• Grant numbers awarded to each author<br>• The full name of each funder<br>• URL of each funder website<br>• Did the sponsors or funders play any role in the study design, data collection and analysis, decision to publish, or preparation of the manuscript?<br><br>Did you receive funding for this work?" | |
| **Competing Interests**<br><br>Use the instructions below to enter a competing interest statement for this submission. On behalf of all authors, disclose any competing interests that could be perceived to bias this work—acknowledging all financial support and any other relevant financial or non-financial competing interests.<br><br>This statement is required for submission and **will appear in the published article** if the submission is accepted. Please make sure it is accurate and that any funding sources listed in your Funding Information later in the submission form are also | The authors have declared that no competing interests exist. |

| | |
|---|---|
| declared in your Financial Disclosure statement.<br><br>View published research articles from *PLOS ONE* for specific examples.<br><br>**NO authors have competing interests**<br><br>Enter: *The authors have declared that no competing interests exist.*<br><br>**Authors with competing interests**<br><br>Enter competing interest details beginning with this statement:<br><br>*I have read the journal's policy and the authors of this manuscript have the following competing interests: [insert competing interests here]*<br><br>* typeset | |
| **Ethics Statement**<br><br>Enter an ethics statement for this submission. This statement is required if the study involved:<br><br>• Human participants<br>• Human specimens or tissue<br>• Vertebrate animals or cephalopods<br>• Vertebrate embryos or tissues<br>• Field research<br><br>Write "N/A" if the submission does not require an ethics statement.<br><br>General guidance is provided below. Consult the submission guidelines for detailed instructions. **Make sure that all information entered here is included in the Methods section of the manuscript.** | Sri Ramachandra Institute of Higher Education and Research |

*Powered by Editorial Manager® and ProduXion Manager® from Aries Systems Corporation*

| | |
|---|---|
| **Format for specific study types**<br><br>**Human Subject Research (involving human participants and/or tissue)**<br>• Give the name of the institutional review board or ethics committee that approved the study<br>• Include the approval number and/or a statement indicating approval of this research<br>• Indicate the form of consent obtained (written/oral) or the reason that consent was not obtained (e.g. the data were analyzed anonymously)<br><br>**Animal Research (involving vertebrate animals, embryos or tissues)**<br>• Provide the name of the Institutional Animal Care and Use Committee (IACUC) or other relevant ethics board that reviewed the study protocol, and indicate whether they approved this research or granted a formal waiver of ethical approval<br>• Include an approval number if one was obtained<br>• If the study involved *non-human primates*, add *additional details* about animal welfare and steps taken to ameliorate suffering<br>• If anesthesia, euthanasia, or any kind of animal sacrifice is part of the study, include briefly which substances and/or methods were applied<br><br>**Field Research**<br><br>Include the following details if this study involves the collection of plant, animal, or other materials from a natural setting:<br>• Field permit number<br>• Name of the institution or relevant body that granted permission | |
| **Data Availability**<br><br>Authors are required to make all data underlying the findings described fully available, without restriction, and from the time of publication. PLOS allows rare exceptions to address legal and ethical concerns. See the PLOS Data Policy and FAQ for detailed information. | Yes - all data are fully available without restriction |

| | |
|---|---|
| A Data Availability Statement describing where the data can be found is required at submission. Your answers to this question constitute the Data Availability Statement and **will be published in the article**, if accepted.<br><br>**Important:** Stating 'data available on request from the author' is not sufficient. If your data are only available upon request, select 'No' for the first question and explain your exceptional situation in the text box.<br><br>Do the authors confirm that all data underlying the findings described in their manuscript are fully available without restriction? | |
| **Describe where the data may be found in full sentences. If you are copying our sample text, replace any instances of XXX with the appropriate details.**<br><br>• If the data are **held or will be held in a public repository**, include URLs, accession numbers or DOIs. If this information will only be available after acceptance, indicate this by ticking the box below. For example: *All XXX files are available from the XXX database (accession number(s) XXX, XXX.).*<br>• If the data are all contained **within the manuscript and/or Supporting Information files**, enter the following: *All relevant data are within the manuscript and its Supporting Information files.*<br>• If neither of these applies but you are able to provide **details of access elsewhere**, with or without limitations, please do so. For example:<br><br>*Data cannot be shared publicly because of [XXX]. Data are available from the XXX Institutional Data Access / Ethics Committee (contact via XXX) for researchers who meet the criteria for access to confidential data.*<br><br>*The data underlying the results presented in the study are available from (include the name of the third party* | All relevant data are within the manuscript and its Supporting Information files. |

Powered by Editorial Manager® and ProduXion Manager® from Aries Systems Corporation

| | |
|---|---|
| *and contact information or URL).*<br>• This text is appropriate if the data are owned by a third party and authors do not have permission to share the data.<br><br>* typeset | |
| Additional data availability information: | Tick here if the URLs/accession numbers/DOIs will be available only after acceptance of the manuscript for publication so that we can ensure their inclusion before publication.; Tick here if your circumstances are not covered by the questions above and you need the journal's help to make your data available. |

Powered by Editorial Manager® and ProduXion Manager® from Aries Systems Corporation

Manuscript
Click here to access/download;Manuscript;Manuscript 17-09-2024.docx

**Original Research**

**Title page**

# Association of NT-proBNP, sST2, Galectin-3, and microRNA-210-3p in heart failure patients with preserved and reduced ejection fraction- a cross-sectional study

Jasmine Chandra A[1], Sudagar Singh RB[2], Mohanalakshmi P[3], Mahesh Kumar K[4], Karthikeyan Rajamani[5], **Santhi Silambanan[6*]**

[1] Department of Biochemistry, Sri Ramachandra Institute of Higher Education and Research, Chennai, India.

[2] Department General Medicine, Sri Ramachandra Institute of Higher Education and Research, Chennai, India.

[3] Department of Biochemistry, Sri Muthukumaran Medical College Hospital and Research Institute, Chennai, India.

[4] Department of Physiology and Biochemistry, Government Yoga and Naturopathy Medical College and Hospital, Chennai, India.

[5] Department of Public Health, Sri Ramachandra Institute of Higher Education and Research, Chennai, India.

[6*] Department of Biochemistry, Sri Ramachandra Institute of Higher Education and Research, Chennai, India.

**Address for communication of corresponding author**:

Dr Santhi Silambanan

email: santhisilambanan@sriramachandra.edu.in, santhisilambanan@gmail.com

# Association of NT-proBNP, sST2, Galectin-3, and microRNA-210-3p in heart failure patients with preserved and reduced ejection fraction- a cross-sectional study


## Abstract

## Background

Heart failure (HF) is a growing health problem and around two percent are affected in the general population. The prevalence seems to be increasing due to improved management of communicable and non-communicable- diseases. Accurate diagnostic markers that have the potential for early diagnosis of HF are lacking. This study aimed to compare the levels of biomarkers -sST2, galectin-3, and microRNA-210-3p with the gold standard marker- NT-proBNP in heart failure patients with preserved and reduced ejection fractions.

## Materials and Methods

The cross-sectional study was conducted on 270 hypertensive heart failure patients in the age group of 30 to 75 years of both genders. The participants with evidence of HF were recruited from the Department of Cardiology in a tertiary care hospital in Chennai, India. Blood samples were obtained for analysis of NT-proBNP, sST2, and galectin-3 were measured by ELISA. For microRNA-210-3p analysis, 20 healthy individuals were included. MicroRNA was analyzed by qRT-PCR. Institutional ethics committee approval and written informed consent were obtained. Statistical analysis was performed using R software version 4.2.1. The obtained data were subjected to the normality of distribution. Based on the type of distribution, appropriate statistical tools were used to analyze the obtained data. p-value $\leq 0.05$ was considered to be statistically significant.

## Results

NT-proBNP, sST2, galectin-3, and microRNA-210-3p were significantly higher in HFrEF compared to HFpEF and showed a correlation among the markers. MAGGIC score showed a positive correlation with all the biomarkers. The cut-off of MAGGIC score was 14, miRNA-210-3p was s 5.03, NT-proBNP was 355.5 and sST2 was 20.11. However, galectin-3 appeared to have limited value, with an AUC of 0.51 and a non-significant p-value of 0.60.

## Conclusion

All the biomarkers were significantly elevated in HFrEF compared to HFpEF; hence, the biomarkers were able to differentiate the various HF cases. MicroRNA-210-3p could be an early marker in the diagnosis of heart failure. The strategy of employing a multi-marker approach could help in the early diagnosis as well as in stratifying the HF patients. This could aid in implementing targeted therapies.



## Keywords

Hear failure, ejection fraction, NT-proBNP, sST2, galectin-3, microRNA-210-3p


## Introduction

Heart failure (HF) is a complex multisystem disorder due to structural and functional alterations in the heart [1]. It affects 64.34 million people, leading to 9.91 million years lost due to disability (YLDs). In the US, HF prevalence has been projected to increase from 2.42% in 2012 to 2.97% in 2030 [2]. HF is emerging as a health burden in Asia as well, with its prevalence ranging from 1.3 and 6.7%, which is considered to be higher than that of Western countries [3]. Among the Asian countries, the prevalences are 1.3%, 1.0%, 6.7%, and 4.5% in China, Japan, Malaysia, and Singapore respectively [4]. In India, the number of new HF cases may increase from 0.118 - 0.708 million in 2000 to 0.214 -1.3 million in 2025 [5].

HF patients are grouped into three phenotypes based on the ejection fraction (EF) of the left ventricle (LV), which is measured by echocardiography (ECHO). HF is categorized as HF with reduced EF (HFrEF) with EF<40%, HF with preserved EF (HFpEF) with EF>50%, and HF with mid-range/ mildly reduced EF (EFmrEF) with EF 41-49% [6]. HFrEF is preceded by various cardiovascular diseases (CVD) especially acute myocardial infarction (AMI), valvular, and other cardiac diseases [7]. Hypertension is the most important cause of HFpEF, with a prevalence of 60 - 89% among HF patients [7]. HFpEF is prevalent among the older population, especially women. Apoptosis and autophagy cause concentric hypertrophy and LV wall stiffening. The prevalence of HFrEF has been declining, while HFpEF is increasing [8]. To date, HF is diagnosed by ECHO examination and measurement of serum natriuretic peptide (NP) levels. Based on these investigations, the chances of missing the early-stage HF could be high, especially when EF is more than 50% as in HFpEF [7].

The diagnosis of HF is based on the Framingham Diagnostic Criteria for HF [9]. Diagnosis and management of HFrEF have been studied in detail and have been implemented effectively in practice [8]. However, HFpEF and HFmrEF are poorly investigated and managed, particularly in developing countries [10]. The biomarkers have the potential to indicate the pathophysiology of the disease process [11]. The European Society of Cardiology (ESC) guidelines (2016) recommend that patients suspected to have acute HF should have their plasma NPs tested [7]. American Heart Association/ American College of Cardiology (AHA/ACC) guidelines on the management of HF recommend the measurement of soluble suppression of tumorigenicity 2 (sST2) in patients with acute HF for a more appropriate stratification [12]. sST2 levels at the time of admission are more valuable than NPs in assessing the risk of mortality one year after admission for acute HF [13]. Galectin-3 (Gal-3) is involved in inflammation, repair, and fibrosis. Thus, in HF, Gal-3 is a predictor of cardiovascular mortality and the frequency of hospital admissions [14]. MicroRNA-210-3p is upregulated in

tissue hypoxic conditions and thus regulates cell differentiation, proliferation, migration, apoptosis, mitochondrial metabolism, and angiogenesis [15]. Since the prevalence of metabolic disorders such as hypertension, diabetes mellitus, and obesity are high; the chances for them to develop cardiac problems are also high. Studies on the application of biomarkers in the diagnosis and prognosis of HF are limited. In poor economic settings, HF imposes huge mortality and mortality, thus increasing the burden on society. This study aimed to determine the association of sST2, Gal-3, and microRNA-210-3p with the gold standard marker, NT-proBNP in heart failure patients with preserved and reduced ejection fractions.

# Materials and Methods

The prospective cross-sectional study was conducted among 270 HF patients of both genders in the age group of 30 to 75 years. The sample size was calculated based on the study by Spinar et al. 2019 [16]. Hypertensive patients with evidence of HF were recruited from the Department of Cardiology at Sri Ramachandra Institute of Higher Education and Research, Chennai, India from 01-12-2020 to 15-02-2023. Initially, the study was decided to be conducted only on hypertensive patients with HF. Due to the COVID-19 pandemic, recruiting patients with hypertension alone was highly challenging. Hence, with the advice of the research advisory committee members, it was decided to include hypertensive HF patients who also had diabetes mellitus; and revised ethics approval accordingly. Thus, among the 270 patients, 168 patients had diabetes mellitus in addition to hypertension. Diagnosis of HF was based on Framingham Heart Failure Diagnostic criteria [9].

The study participants were subjected to the transthoracic 2D Doppler echocardiography using Phillips and GE Healthcare echocardiography with patients lying supine in theleft lateral decubitus position. According to the definition of European and United States guidelines, the normal EF is 52-72% in men and 54-74% in women [17]. According to Chengode, EF > 55% is considered to be normal. It can be 45–54%, 30 - 44%, and < 30% in

mild, moderate, and severe LV dysfunction respectively [18]. Anthropometric characteristics such as body mass index (BMI) in $Kg/m^2$ and waist-to-hip ratio (WHR) were measured as per the Asia-Pacific guidelines [19]. Systolic and diastolic blood pressures were measured using standard procedure and patients were identified as hypertensives based on the JNC7 blood pressure classification [20]. According to the International Expert Committee, diabetes mellitus was diagnosed based on glycated hemoglobin (HbA1c) ≥6.5%, fasting plasma glucose ≥126 mg/dL, and 2-hour plasma glucose or random plasma glucose ≥200 mg/dL [21].

LV ejection fraction (EF) remains the major parameter for phenotyping, diagnosis, prognosis, and treatment decisions in HF. In the present study, EF of 50% was used as the cut-off to categorize the participants into two groups group 1 – HF with EF ≤ 49% and group 2 - HF with EF ≥ 50%. As per the 201[illegible] ESC Guidelines, EF of 40- 49% is categorized as HFmrEF. HFmrEF is an intermediate clinical entity between HFrEF and HFpEF, but more similar to HFrEF of the high prevalence of AMI in these patients [7]. Patients with HFmrEF are similar to HFrEF in being younger males, with increased systolic blood pressure, and reduced LV and atrial dilatation. Also, HFmrEF is found to benefit from HFrEF treatment strategies [7]. In the present study, based on the above literature data, HFmrEF has been included in HFrEF.

The participants were grouped into group 1 with HF patients with EF ≤ 49% and group 2 with EF ≥50%. Patients with acute HF in the past 3 months, AMI in the past 6 weeks, disorders of thyroid, lung, renal, and liver, pregnancy, cancer, systemic infectious diseases, connective tissue disorders, and patients on anticancer drugs, steroids, and oral contraceptive pills were excluded from the study. The Meta-Analysis Global Group in Chronic Heart Failure (MAGGIC) mortality risk score was calculated in all the study participants. The MAGGIC score consists of thirteen clinical predictor variables such as age, sex, body mass index (BMI), systolic blood pressure (SBP), ejection fraction (EF), serum creatinine level, participant was a

current smoker, diabetes mellitus, and chronic obstructive pulmonary disease, belongs to New York Heart Association (NYHA) class, heart failure (HF) diagnosed for at least eighteen months, use of beta-blockers and angiotensin-converting enzyme (ACE) inhibitors.

NT-pro BNP, sST2, and Gal-3 were measured by ELISA:

- NT-proBNP: measurement range: 7.5 - 120 pg/mL, sensitivity: 0.5 pg/mL, CV%: intra-assay < 9% and inter-assay < 11%; Biological Reference Interval: ≤74 years: < 125 pg/mL, and ≥ 75 years: < 450 pg/mL [17].
- sST2: measurement range: 0.31-20.0 ng/mL, sensitivity: 0.19 ng/mL, CV%: intra-assay 5.4% and inter-assay 6.1%, Biological Reference Interval: 14.42 ng/mL [18].
- Gal-3: measurement range: 0.16-10.0 ng/mL, sensitivity: 0.16ng/mL, CV%: intra-assay 5.56% and inter-assay 7.27%, Biological Reference Interval: < 17.8 ng/mL [19].

MicroRNA-210-3p was analyzed by qRT-PCR. MicroRNA 210-3p was expressed as cycle threshold folds. Analysis of microRNA-210-3p: Methodology: RT-PCR, microRNA was isolated by PureFast® miRNA mini spin purification kit [20].

>hsa-miR-210-3p MIMAT0000267; CUGUGCGUGUGACAGCGGCUGA

Analysis was done on 100 samples; with 20 controls (apparently healthy individuals without hypertension or diabetes mellitus or HF), 40 patients of group 1 and 40 patients of group 2. Fold change was calculated as follows:

$$\Delta CT = CT - CT$$

$$\Delta\Delta CT = \Delta CT - \Delta CT_{control}$$

$$FC = 2^{-\Delta\Delta CT}$$

(CT: cycle threshold, FC: fold change)

## Ethics Statement

Ethics approval was obtained from the institutional ethics committee. Ethics number: IEC-NI/19/FEB/68/09, dated 10.11.2020. Written informed consent was obtained from all the study participants.

## Statistics used

Statistical analysis was performed using R software version 4.2.1. The obtained data were subjected to the Kolmogorov-Smirnov test to check for normality of distribution. Continuous variables were expressed as mean and standard deviation or median and interquartile range and the student 't' test or Mann-Whitney U test was used to compare the data between the groups. Categorical variables were expressed as frequency and percentage; the Chi-Square test or Fischer-exact test was used to compare the data. The Pearson correlation coefficient was used to test the correlation among the variables. The receiver operating characteristics (ROC) curve was used to find the area under the curve (AUC). The Youden index was used to find the cut-off values of biomarkers. P value ≤0.05 was considered statistically significant.

Large Language Models (LLMs), such as ChatGPT were not used by any of the authors in this manuscript.

# Results

Based on the EF cut-off of 50%, 170 patients have EF ≥ 50%, while the remaining 100 HF patients have reduced EF of ≤ 49%. Figure 1 shows the flow chart indicating the recruitment of participants into the study along with patient characteristics. Table 1 shows the demographic data of the study participants.

**Figure 1 shows the flow chart displaying the recruitment of study participants and their characteristics**

**Table 1 [illegible]ows the demographic data of the study participants**

| **Characteristics** | **Group 1 (n=100) HF with EF ≤ 49%** | **Group 2 (n=170) HF with EF ≥ 50%** | **p-value** |
|---|---|---|---|
| Age (Years) | | | |
| < 45 n (%) | 12 (12) | 5 (2) | <0.001** |

| | | | |
|---|---|---|---|
| 46-60 n (%) | 26 (26) | 118 (70) | |
| 61 -75 n (%) | 62 (62) | 47 (28) | |
| Gender | | | |
| Female n (%) | 34 (34) | 72 (42) | 0.21 |
| Male n (%) | 66 (66) | 98 (58) | |
| BMI ($Kg/m^2$) | | | 0.90 |
| Normal weight n (%) | 26 (26) | 42 (25) | |
| Overweight n (%) | 40 (40) | 73 (43) | |
| Obese n (%) | 34 (34) | 55 (32) | |
| Hypertension | | | |
| Present n (%) | 100 (100) | 170 (100) | 1.0 |
| Absent n (%) | 0 (0) | 0 (0) | |
| T2DM | | | |
| Present n (%) | 66 (66) | 102 (60) | 0.37 |
| Absent n (%) | 34 (34) | 68 (40) | |
| NYHA Class (n/%) | III (59/59) | I (160/94) | <0.001** |
| | IV (41/41) | II (10/6) | |

*NYHA: New York Heart Association; Expressed as frequency & percentage; Chi-Square test was done; **: p-value statistically ~~highly~~ significant*

Among the distribution of age, the majority of patients in group 1 were in the older age of 61-75 years, whereas in group 2, the majority of them were middle-aged persons. Most of the participants were males in both groups. Based on BMI, most of them were in overweight and obese categories compared to normal weight in both groups. According to NYHA class, participants in Group 1 were in NYHA classes III and IV, whereas in Group 2, the participants were in classes I and II (Table 1) Among the participants, 33% were smokers, 35% were alcoholics, and 46% were vegetarians.

The distribution of biochemical parameters such as lipid profile and cardiac biomarkers is shown in Table 2.

**Table 2: Shows levels of biomarkers among the participants**

| Characteristics | **Group 1 (n=100)**<br><br>**HF with EF ≤ 49%** | **Group 2 (n=170)**<br><br>**HF with EF ≥ 50%** | **p-value** |
|---|---|---|---|
| TC (mg/dL) | 205.29 (46.42) | 193.72 (38.19) | 0.01* |
| TG (mg/dL) | 155.80 (63.06) | 149.55 (73.58) | 0.41 |
| HDL (mg/dL) | 43.74 (13.00) | 42.76 (10.38) | 0.92 |
| LDL (mg/dL) | 126.02 (39.78) | 124.28 (33.26) | 0.54 |
| NT-proBNP (pg/mL) | 482.74 (110.94) | 287.8 (81.62) | 0.001** |
| sST2 (ng/mL) | 38.24 (13.15) | 21.40 (7.42) | 0.001** |
| Gal-3 (ng/mL) | 24.93 (10.84) | 17.77 (6.03) | 0.001** |
| MiRNA-210-3p (folds) | 11.89 (31.11) | 1.08 (1.36) | 0.03* |
| MAGGIC score (points) | 22.94 (5.92) | 8.05 (4.64) | 0.001* |

*TC: total cholesterol; TG: triglyceride; HDL: high-density lipoprotein, LDL: low-density lipoprotein, NT-proBNP: N-terminal- pro-brain natriuretic peptide, sST2: soluble suppression of tumorigenicity, Gal-3: Galectin-3; MiRNA: micro ribonucleic acid; MAGGIC: Meta-Analysis Global Group in Chronic Heart Failure*
*Expressed as mean and SD; Student t-test was used; **: p-value statistically highly significant *: p-value statistically significant.*

Among the lipids, all the variables were higher in group 1 than in group 2, but a statistically significant difference was obtained only for total cholesterol. All the cardiac biomarkers showed statistically significant differences between the groups. All the markers including microRNA-210-3p were higher in group 1 compared to group 2. Similarly, MAGGIC score also showed the same type of response among the groups (Table 2).

Table 3 shows the levels of biomarkers among the participants according to the gender of the groups.

**Table 3[illegible]hows levels of biomarkers among the participants based on gender**

| Characteristics | HF Female (n=106) | | p-value | HF Male (n=164) | | p-value |
|---|---|---|---|---|---|---|
| | Group 1: EF ≤ 49% | Group 2: EF ≥ 50%, | | Group 1: EF ≤ 49%, | Group 2: EF ≥ 50%, | |

| | (n=34) | (n=72) | | (n=66) | (n=98) | |
|---|---|---|---|---|---|---|
| Age | 61.18 (12.02) | 55.69 (9.86) | 0.01* | 62.09 (11.25) | 53.63 (10.91) | <0.001** |
| NYHA class (n/%) | III (23/22) | I (68/64) | <0.001** | III (36/22) | I (92/56) | <0.001** |
| | IV (11/10) | II (4/4) | | IV (30/18) | II (6/4) | |
| TC | 199.0 (47.52) | 190.10 (36.04) | 0.28 | 208.53 (45.87) | 196.39 (39.67) | 0.07 |
| TG | 161.74 (63.30) | 129.26 (47.30) | 0.003** | 152.74 (63.20) | 164.45 (85.23) | 0.34 |
| HDL | 42.82 (12.85) | 43.93 (10.65) | 0.64 | 44.21 (13.15) | 41.90 (10.14) | 0.20 |
| LDL | 118.62 (41.40) | 122.21 (31.01) | 0.61 | 129.83 (38.69) | 125.81 (34.90) | 0.48 |
| FPG | 146.50 (63.98) | 121.44 (37.91) | 0.01* | 133.65 (64.89) | 130.95 (49.67) | 0.76 |
| PPP[illegible] | 223.58 (91.58) | 160.26 (72.38) | <0.001** | 200.57 (92.42) | 169.44 (86.10) | 0.02* |
| HbA1c | 8.43 (2.36) | 6.62 (1.38) | <0.001** | 7.83 (2.75) | 6.81 (1.70) | 0.003** |
| BUN | 12.68 (5.88) | 9.90 (3.30) | 0.002** | 14.7 (8.21) | 13.58 (1.78) | 0.19 |
| Creatinine | 0.86 (0.40) | 0.78 (0.24) | 0.20 | 1.04 (0.38) | 0.94 (0.29) | 0.058 |
| Hb | 11.62 (1.63) | 11.83 (1.95) | 0.58 | 12.72 (2.10) | 13.58 (1.79) | 0.005** |
| NT-proBNP | 482.05 (168.08) | 291.75 (95.11) | <0.001** | 483.09 (66.35) | 284.92 (92.59) | <0.001** |
| sST2 | 38.77 (15.30) | 23.33 (18.19) | <0.001** | 37.97 (12.01) | 19.98 (9.41) | <0.001** |
| Gal-3 | 24.0 (11.22) | 18.91 (8.71) | 0.01* | 25.40 (10.69) | 16.94 (7.86) | <0.001** |
| MiRNA-210-3p# | 2.52 (0.78-6.53) | 0.2 (0.09-0.64) | 0.016* | 1.02 (0.14-6.01) | 0.25 (0.12-0.95) | 0.07 |
| MAGGIC Score | 20.88 (5.96) | 6.85 (4.34) | <0.001** | 24.0 (5.65) | 8.93 (4.67) | <0.001** |

*TC: total cholesterol; TG: triglyceride; HDL: high-density lipoprotein, LDL: low-density lipoprotein, NT-proBNP: N-terminal- pro-brain natriuretic peptide, sST2: soluble suppression of tumorigenicity, Gal-3: Galectin-3; MiRNA: micro ribonucleic acid; MAGGIC: Meta-Analysis Global Group in Chronic Heart Failure*

*Expressed as mean and SD; Student t-test was used; #: expressed as median and interquartile range; Mann-Whitney U test was used; **: p-value statistically highly significant *: p-value statistically significant.*

Among the distribution of age, in both genders, group 1 HF patients were in the older age group compared to group 2. Among the lipids, female patients showed significant alterations in triglyceride levels among the groups compared to males. Females showed significant differences in fasting and postprandial glucose levels and HbA1c, while males showed significant differences in postprandial glucose and HbA1c levels. The alterations in the glycaemic parameters probably were reflected as altered triglyceride levels in females. Blood urea nitrogen was significantly altered in females compared to males. However, creatinine was not significantly altered in both genders. Cardiac biomarkers such as NT-proBNP, sST2, galectin-3, and MAGGIC score were significantly altered when compared among the groups in both genders. Even though microRNA-210-3p was elevated in group 1 in both genders, statistical significance was seen only in females (Table 3)

The participants were grouped into those with age less than 50 years and those with more than 51 years. Table 4 shows the levels of the biomarkers among the participants based on the age distribution among the groups.

Table 4**: Shows levels of biomarkers among the participants based on age**

| Characteristics | Age ≤ 50yrs (n=78) | | p-value | Age ≥ 51yrs (n=192) | | p-value |
|---|---|---|---|---|---|---|
| | Group 1: HF with EF≤49% (n= 17) | Group 2: HF with EF≥ 50% (n= 61) | | Group 1: HF with EF ≤ 49% (n=83) | Group 2: HF with EF ≥ 50% (n = 109) | |
| Gender | | | | | | |
| Male (n/%) | 10 (13) | 42 (55) | 0.31 | 56 (29) | 57 (29) | 0.13 |
| Female (n/%) | 7 (9) | 19 (23) | | 27 (14) | 52 (28) | |
| NYHA class (n/%) | III (13/17)<br>IV (4/5) | I (58/74)<br>II (3/4) | 0.01* | III (46/24)<br>IV (37/19) | I (103/54)<br>II (6/3) | <0.001** |
| TC | 208.33 (39.65) | 201.23 (38.70) | 0.50 | 204.55 (47.87) | 189.52 (37.43) | 0.01* |
| TG | 160.59 (59.16) | 163.02 (77.77) | 0.90 | 154.82 (64.12) | 142.01 (70.37) | 0.19 |
| HDL | 44.94 (11.91) | 43.13 (13.37) | 0.61 | 43.49 (13.26) | 42.55 (8.31) | 0.54 |
| LDL | 128.88 (30.63) | 130.62 (31.99) | 0.84 | 125.43 (41.54) | 120.73 (33.57) | 0.38 |
| FPG | 141.86 (81.47) | 128.0 (48.32) | 0.37 | 136.95 (58.75) | 126.62 (43.87) | 0.16 |

| | | | | | | |
|---|---|---|---|---|---|---|
| PPPG | 269.88 (30.63) | 163.50 (78.22) | <[illegible]1 | 200.53 (79.50) | 166.10 (81.22) | 0.003** |
| HbA1c | 7.49 (2.98) | 6.62 (1.68) | 0.12 | 8.14 (2.55) | 6.79 (1.51) | <0.001** |
| BUN | 12.06 (6.59) | 9.9 (3.1) | 0.057 | 14.41 (7.68) | 10.72 (3.69) | <0.001** |
| Creatinine | 0.88 (0.40) | 0.86 (0.25) | 0.80 | 1.00 (0.40) | 0.88 (0.30) | 0.01* |
| Hb | 12.70 (2.10) | 13.19 (2.02) | 0.38 | 12.26 (2.00) | 12.65 (2.04) | 0.18 |
| NT-proBNP | 426.66 (50.15) | 266.33 (94.24) | <0.001** | 494.22 (116.65) | 299.83 (91.24) | <0.001** |
| sST2 | 43.02 (17.12) | 20.97 (10.09) | <0.001** | 37.26 ( 12.07) | 21.64 (15.64) | <0.001** |
| Gal-3 | 27.39 (10.53) | 17.62 (8.86) | <0.001** | 24.42 (10.90) | 17.86 (7.99) | <0.001** |
| MiRNA-210-3p# | 0.53 (0.05-3.01) | 0.23 (0.13-2.38) | 0.59 | 1.89 (0.16-14.26) | 0.25 (0.10-0.87) | 0.001** |
| MAGGIC score | 16.65 (4.61) | 5.52 (2.16) | <0.001** | 24.23 (5.32) | 9.46 (5.03) | <0.001** |

*TC: total cholesterol; TG: triglyceride; HDL: high-density lipoprotein, LDL: low-density lipoprotein, NT-proBNP: N-terminal- pro-brain natriuretic peptide, sST2: soluble suppression of tumorigenicity, Gal-3: Galectin-3; MiRNA: micro ribonucleic acid; MAGGIC: Meta-Analysis Global Group in Chronic Heart Failure*
*Expressed as mean and SD; Student t-test was used; #: expressed as median and interquartile range; Mann-Whitney U test was used; **: p-value statistically highly significant *: p-value statistically significant.*

There was no significant difference when compared between the genders concerning age. NYHA class showed significant differences among the groups in both age groups. Among the lipids, only total cholesterol showed a significant difference among the groups when the age of the participants was more than 51 years. Among glycemic parameters, post-prandial glucose and HbA1c levels are significantly increased in individuals more than 51 years of ag[illegible] Among renal parameters, both urea and creatinine showed significantly higher levels in individuals of more than 51 years of age. NT-proBNP, sST2, galectin-3and MAGGIC scores were significantly increased in group 1 irrespective of the age of the participants. However, MicroRNA-210-3p showed significantly high values in group 1 individuals of more than 51 years of age (Table 4[illegible]

Figures 2A and 2B show the microRNA-210-3p amplification patterns in groups [illegible]nd 2 of

heart failure patients.

**Figure 2A and 2B: MicroRNA amplification patterns among group [illegible]nd group 2 HF patients**

The analysis of biomarkers for heart failure prediction revealed that the MAGGIC score demonstrated the best overall performance with the highest AUC of 0.86 (95% CI: 0.86-0.90) (p=0.001), a cut-off score of 14 with sensitivity of 95% and specificity of 89%, suggesting its reliability for risk stratificatio[illegible]mong the individual biomarkers, miRNA-210-3p showed the most promise as a novel biomarker, outperforming traditional markers with a higher AUC of 0.79 (95% CI: 0.68-0.89), sensitivity of 87%, and specificity of 54% at a cut-off level of 5.03. Traditional biomarkers showed moderate predictive ability: NT-proBNP had an AUC of 0.65 (95% CI: 0.58-0.71) with 64% sensitivity and 46% specificity at a cut-off of 355.5, while sST2 had an AUC of 0.58 (95% CI: 0.52-0.65) with 60% sensitivity and 46% specificity at a cut-off of 20.11. Galectin-3 appeared to have limited value, with an AUC of 0.51 (95% CI: 0.44-0.58) and a non-significant p-value of 0.60 (Table 5, Fig 3)

**Table 5: Cut-off level of the biomarkers in patients with HF**

| **Variable** | **AUC** | **SE** | **p-value** | **95%CI** | **Cut-off level** | **Sensitivity (%)** | **Specificity (%)** |
|---|---|---|---|---|---|---|---|
| NT-proBNP | 0.65 | 0.03 | 0.001** | 0.58-0.71 | 355.5 | 64 | 46 |
| sST2 | 0.58 | 0.03 | 0.01* | 0.52-0.65 | 20.11 | 60 | 46 |
| Gal-3 | 0.51 | 0.03 | 0.60 | 0.44-0.58 | 17.58 | 55 | 57 |
| MiRNA-210-3p | 0.79 | 0.05 | <0.001** | 0.68-0.89 | 5.03 | 87 | 54 |
| MAGGIC score | 0.86 | 0.02 | 0.0[illegible] | 0.86-0.90 | 14 | 95 | 89 |

*NT-proBNP: N-terminal- pro brain natriuretic peptide, sST2: soluble suppression of tumorigenicity, Gal-3: galectin-3; MiRNA: micro ribonucleic acid; MAGGIC: Meta-Analysis*

*Global Group in Chronic Heart Failure*
***: p value statistically highly significant *: p value statistically significant.*

**Figure 3: ROC curve showing the cut-off level of the biomarkers in patients with HF**

Table 6 shows the correlation of NT-proBNP with MAGGIC score, other cardiac markers, microRNA-210-3p, lipid profile, and HbA1c

**Table 6: Correlation between NT-proBNP and other novel biomarkers in patients with HF**

| Correlation Matrix | MAGGIC score | NT-proBNP | sST2 | Gal-3 | MiR-210-3p | TC | TG | HDL | LDL | HbA1c |
|---|---|---|---|---|---|---|---|---|---|---|
| **MAGGIC score** | - | | | | | | | | | |
| **NT-proBNP** | 0.676<br><0.001 | — | | | | | | | | |
| **sST2** | 0.405<br><0.001 | 0.416<br><0.001 | _- | | | | | | | |
| **Gal-3** | 0.281<br><0.00[illegible] | 0.231<br><0.001 | 0.136<br>0.025 | - | | | | | | |
| **MiR-210-3p** | 0.136<br>0.167 | 0.067<br>0.497 | 0.087<br>0.377 | 0.160<br>0.102 | - | | | - | | |
| **TC** | 0.063<br>0.301 | 0.118<br>0.053 | 0.121<br>0.047 | 0.011<br>0.858 | 0.03<br>0.761 | - | | | | |
| **TG** | 0.016<br>0.799 | 0.003<br>0.963 | 0.012<br>0.848 | 0.078<br>0.203 | 0.173<br>0.077 | 0.337<br><0 .001 | - | | | |
| **HDL** | -0.081<br>0.183 | -0.04<br>0.515 | 0.098<br>0.108 | -0.061<br>0.318 | 0.015<br>0.876 | -0.501<br><0.001 | -0.061<br>0.32 | - | | |
| **LDL** | 0.058<br>0.341 | 0.041<br>0.507 | 0.034<br>0.578 | 0.003<br>0.960 | 0.010<br>0.921 | 0.820<br><0 .001 | 0.339<br><0.001 | -0.321<br><0.001 | - | |
| **HbA1c** | 0.286<br><0.001 | 0.268<br><0.001 | 0.159<br>0.012 | 0.045<br>0.484 | 0.152<br>0.124 | 0.107<br>0.093 | 0.024<br>0.702 | -0.065<br>0.306 | 0.076<br>0.232 | - |

*NT-proBNP: N-terminal- pro-brain natriuretic peptide, ST2: suppression of tumorigenicity, RNA: ribonucleic acid*
***: P value statistically highly significant *: P value statistically significant.*

Widely accepted MAGGIC score showed a positive correlation with NT-proBNP, sST2, and galectin-3. There was a positive correlation among NT-proBNP, sST2, and galectin-3 However, microRNA did not show any correlation with any of the variables probably due to the small sample size. NT-proBNP and MAGGIC scores showed a positive correlation with HbA1c. sST2 showed a positive correlation with total cholesterol and HbA1c. Total cholesterol

showed a positive correlation with triglyceride and LDL cholesterol and a negative correlation with HDL cholesterol (Table 6).

# Discussion

Heart failure (HF) is the consequence of various cardiac and noncardiac conditions [1]. In humans, after the sixth decade of life, the incidence of HF doubles in men whereas it triples in women [27]. The Cardiology Society of India-Kerala Acute Heart Failure Registry (CSI-KHFR) indicates that India is facing a huge threat of HF due to its large population, and increased prevalence of diabetes mellitus, obesity, hypertension, pulmonary diseases, and sedentary lifestyle [28]. In India, states such as Kerala, Punjab, and Tamil Nadu are the most affected, probably due to the increased prevalence of dyslipidemia and hypertension [29]. The pathological heart remodeling also varies according to the etiology of HF. AMI stimulates sterile inflammation, which in turn leads to replacement fibrosis and eccentric hypertrophy in HFrEF. HFpEF due to hypertension has concentric hypertrophy and interstitial fibrosis in the heart. Therapeutic options that are available for HFrEF are shown to be not very beneficial in HFpEF. Diagnostic procedures that may be able to distinguish the underlying pathological processes may be useful in the less understood HF phenotypes [7].

In the present study, the presenting clinical features in group 1 were dyspnea, pedal edema, and palpitation while group 2 patients complained mainly of fatigue. In group 1 (HF≤49%), 62% of patients were in the advancing age of 61 to 75 years, while in group 2 (HF≥50%), 70% were 46 to 60 years of age. The younger age involvement in group 2 indicated the earlier onset of symptoms due to pathogenesis set by the underlying disorder such as hypertension and type 2 diabetes mellitus (T2DM). Males were predominantly affected irrespective of EF. Males tend to be obese, with earlier onset of hypertension and T2DM. Most of the participants were in the overweight category in both groups. This indicated that obesity is an impending risk for the development of hypertension and T2DM with consequent HF

(Table 1). According to Teramoto et al, the mean age of HF patients in India is 58 to 68 years, which is about a decade younger than that of Western HF patients. The prevalence of HFpEF is higher than that of HFrEF. HFpEF is higher in women, while HFrEF is higher in men. HFpEF phenotype tends to increase with the aging of the population, especially among patients of more than 64 years of age [30].

In the present study, group 1 patients had higher total cholesterol than that of group 2 (p=0.01) (Table 2). Since the patients were hypertensives while 60 of them had T2DM also, atherosclerotic changes in the blood vessels could be present as evidenced by dyslipidemia. Studies done by Jarab et al, have shown that elevated LDL and decreased HDL are associated with reduced cardiac function. Inflammation associated with dyslipidemia plays a major role in the exacerbation of HF [31]. Yao et al, have concluded that dyslipidemia induced by oxidative stress, inflammation, altered autophagy, microvascular density, and cardiomyocyte mitochondrial dysfunction leads to impaired cardiac structure and function. The measures adopted to decrease serum lipids are found to improve the ventricular functioning capacity [32].

The MAGGIC score is used to predict mortality as well as stratify the patients with HF. It can be done with the routinely available parameters [22]. However, MAGGIC score is not able to predict the frequency of hospitalization, and morbidity and it does not include brain natriuretic peptide levels. Hence, relying on biomarkers is a better choice for diagnosis as well as prognosis of HF. According to Piek et al, ventricular myocyte stress stimulates the synthesis and release of natriuretic peptides (NPs) [33]. BNP or NT-proBNP is considered to be the gold standard marker for the diagnosis and prognosis of HF [34]. Also, NPs can identify asymptomatic HF in individuals with poor LV function. BNP acts by regulating the renin-angiotensin-aldosterone system (RAAS) and the sympathetic nervous system [35]. Thus, it causes vasodilatation, increases glomerular filtration rate (GFR), and prevents salt

reabsorption, thus, promoting natriuresis and diuresis [36]. In the present study, NT-proBNP levels were 482.74 ± 110.94 pg/mL and 287.81 ± 93.45pg/mL in groups 1 and 2 respectively (p=0.001) (Table 2). This indicated that group 1 patients had more severe LV volume overload than those group 2 patients. According to the study by Kang et al, although patients with HFpEF have lower NT-proBNP levels, the prognosis is similar to that of HFrEF [37]. Patients who exhibit less than 30% reduction in BNP levels show an increased risk of 180-day mortality [38]. In non-acute conditions, especially when ECHO is not immediately available, elevated NPs may help to establish an initial diagnosis [39]. According to Cao et al, BNP levels help clinicians to distinguish whether the dyspnea is of cardiac or noncardiac origi[illegible]0].

NT-proBNP has certain limitations, which preclude NPs from being used as a gold standard marker. According to Cao et al, different cut-off values used are based on whether the HF is acute or chronic [40]. Yancy et al, have concluded that there is no consensus in arriving at the age-adjusted NP cut-off levels [40]. Old age and smoking are associated with elevated NT-proBNP in males. Diabetes mellitus is inversely associated with NT-proBNP levels in females [41]. In obesity, there is decreased production and increased clearance of NPs which results in disproportionately low NP levels in obese HF. Hence, lower cut-offs are needed for diagnosing HF in patients with obesity [42]. BNP levels rise with a fall in GFR. When GFR is <60mL/min/1·73 $m^2$, LV hypertrophy (LVH) develops along with systolic and diastolic LV dysfunctions. The PRIDE study showed that a higher cut-off of NT-proBNP of >1,200pg/mL may be used for diagnosing HF in chronic kidney disease (CKD) [43].

The 201[illegible]CC Foundation/ AHA guidelines have recommended that in addition to NPs, biomarkers of inflammation and fibrosis may be used to refine the prognostic stratification of HF [44]. The 2016 ESC Guidelines stated that soluble Suppression of Tumorigenicity 2 (sST2) and other fibrosis biomarkers can be used in the clinical practice of HF [45]. sST2 is a member of the Toll-like receptor binding interleukin (IL)-33 [11].

Alternative splicing and processing of the mRNA of the ST2 gene leads to the production of either sST2 or ST2L [46]. When the IL-33/ST2L signaling mechanism is activated, it results in anti-apoptotic, anti-fibrotic, and anti-hypertrophic effects [11]. sST2 production is induced by circulatory overload causing mechanical stretch, inflammation, and fibrosis [46]. sST2 acts as a decoy receptor for IL-33 and blocks the action of IL-33, thus resulting in myocardial hypertrophy [11]. Generally, sST2 levels are low, enabling IL-33 to maintain cardioprotective effects. In HF, the elevated sST2 levels prevent ST2L from binding to IL-33. IL-33/sST2 complex thus formed, does not have a protective role on the heart. sST2 is considered a novel biomarker of cardiac strai[illegible]7]. In the present study, sST2 levels were 38.23±13.14ng/mL and 21.40±13.87 in groups 1 and 2 respectively (p=0.001) (Table 2[illegible]his probably indicate[illegible]at sST2 indicated myocardial fibrosis, which could be higher in group 1 than in group 2.

According to Aimo et al., acute HF patients with elevated sST2 levels at the time of discharge, are considered to be at high risk for prolonged hospital stay, increased dosage of HF drugs, and frequent hospital visits. In HFpEF, sST2 concentrations are associated with systemic inflammation, right ventricle (RV) dysfunction, and systemic congestion [46]. Increased plasma sST2 concentrations are associated with all-cause and cardiovascular mortality [48]. sST2 could also be a promising tool to identify the risk of sudden cardiac death (SCD) in patients with low EF. The lower biological variability of sST2 in comparison to NT-proBNP, makes sST2 a promising marker for assessing the prognosis of HF patients [49]. Soluble ST2 levels rise several weeks before a cardiac event; thus, serving as a sensitive marker in the diagnosis of HF [13]. sST2 predicts end-point events better than NT-proBNP since sST2 is not affected by BMI, renal function, age, etiology, or other traditional risk factors of HF [49]. Elevated sST2 levels predict ventricular arrhythmia, LV diastolic dysfunction, increased RV systolic pressure, and hypokinesis in chronic HF [46]. Asymptomatic individuals have higher levels of sST2 than that of healthy individuals [49]. ST2 levels in combination with NT-

proBNP could be better in risk stratification [50]. By measuring sST2 levels, patients at risk of adverse LV remodeling can be identified [51].

According to the 2017 ACC/AHA/HFSA Guidelines, galectin-3 (Gal-3) is suggested to be a diagnostic marker for risk stratification and prognosis evaluation in HF patients [52]. A study done by Gehlken et al has concluded that when Gal-3 is used as a sole marker, it could predict a 60-day prognosis in acute HFpEF. In hypertensive individuals, Gal-3 can detect early onset HFpEF. Its levels are directly related to the severity of diastolic dysfunction [53]. Although sST2 and Gal-3 have similar prognostic performances in both HFrEF and HFpEF, sST2 is considered to be superior to Gal-3. Gal-3 is a member of the β-galactoside-binding lectin family and is produced by macrophages [11]. It is highly stable and resistant to hemodynamic alterations, and is an early indicator of cardiac fibrosis, ventricular remodeling, and renal impairment in HF patients; however, it has low specificity [54]. Under hypoxic or hypocaloric conditions, Gal-3 expression is upregulated, through the activity of hypoxia-inducible factor- 1 alpha (HIF-1α) and nuclear factor – kappa beta (NF-κB); further formed Gal-3 enters systemic circulation. Gal-3 activates mitogen-activated protein kinase (MAPK) signal transduction pathway, leading to cell proliferation and cell survival [6]. In the present study, serum Gal-3 levels were 24.93±10.84 ng/mL and 17.77±8.29 in groups 1 and 2 respectively ($p=0.001$) (Table 2). According to Gehlken et al, Gal-3 plays an important role in inflammation, fibrosis, and ventricular remodeling [53]. Gal-3 is an independent predictor of mortality in patients with moderate to advanced chronic HF [55].

Studies are being done to assess the diagnostic and prognostic potential of epigenetic markers such as micro-RNAs (miRNA), long non-coding RNAs, and exosome cargo, because of their potential role in the early phases of cardiac dysfunction. miRNAs play critical roles in heart development, function, and response to ischemia/reperfusion injury. There is increasing evidence, that miRNAs may have a key role in the pathogenesis of HF through the regulation

of genes involved in adverse ventricular remodeling [56]. The dysregulation of miRNAs, and their target genes, are related to signaling pathways of HF, including cardiac hypertrophy, oxidative stress, cardiac fibrosis, etc [57]. The circulating microRNAs are relatively stable, sensitive, specific, and easily detectable by non-invasive methodology, hence, circulating microRNAs may be ideal candidate biomarkers and drug targets for HF [57]. In response to hypoxia, a specific set of microRNAs, called hypoxamirs are upregulated [56]. In the present study, microRNA 210-3p showed increased expression of 11.89-fold in group 1 and 1.08-fold in group 2 compared to controls. There was a statistically significant difference with p value of 0.031 (Table 2, Fig 2). According to Shen et al, the most down-regulated miRNA is miR-30c, and the most upregulated is miR-210-3p [57]. Through the HIF-1α pathway, miR-210-3p is independently associated with HF, cardiovascular mortality, and cardiac regeneration. miR-210-3p is found to inhibit apoptosis of cardiomyocytes. HF patients who have increased expression levels of miR-210-3p show frequent HF decompensations and adverse ventricular remodeling [56]. MiRNAs can be used alone or in combination with other biomarkers of HF such as BNP [58].

Table 3 shows the distribution of study characteristics according to the gender of the participants. In females, the alterations in the glycemic parameters probably were in parallel with that of altered triglyceride levels. Altered blood urea with normal creatinine levels indicated that females are predisposed to prerenal uremia earlier compared to males, probably due to the anatomical architecture of females. Group 1 patients had higher levels of cardiac markers and MAGGIC scores when compared to group 2 patients. Even though microRNA-210-3p was elevated in group 1 in both genders, significance was seen only in females. MicroRNA could serve as a better diagnostic and prognostic marker, since females tend to have poor prognosis compared to males (Table 3) Table 4 shows the distribution of study parameters according to the age above or below the age of 50 years. Among the lipids, only

total cholesterol showed a significant difference among the groups when the age of the participants was more than 51 years. Among glycemic parameters, post-prandial glucose and HbA1c levels are significantly increased in individuals more than 51 years of age. Among renal parameters, both urea and creatinine showed significantly higher levels in individuals of more than 51 years of age. All these changes indicated that the long-standing metabolic disorders cause end-organ damage (Table 4).

The receiver operating characteristics (ROC) curve showed an area under the curve (AUC) for NT-proBNP of 0.65 (p=0.001). The cut-off value of NT-proBNP was 355.5pg/mL with sensitivity and specificity of 64% and 46% respectively (Table 5, Fig 3). A normal electrocardiogram and/or plasma concentrations of BNP <35 pg/mL and/or NT-proBNP <125 pg/mL may be used in the diagnosis of HF [40]. In young healthy adults, BNP and NT-proBNP levels are <25pg/mL and <70pg/mL respectively [59]. When BNP is <100pg/mL, HF may not be the cause for breathlessness and alternative etiologies for dyspnea should be investigated. If the BNP level is 100 - 500pg/mL, clinical findings are needed to support the diagnosis of HF. When BNP is > 500pg/mL, HF is present, and quick management for HF is needed [40]. The AUC for sST2 of 0.58 with a 95% CI of 0.52 to 0.65 (p=0.01). The cut-off value of sST2 was 20.1ng/mL with sensitivity and specificity of 60% and 46% respectively. (Table 5, Fig 3) Since sST2 shows less biological intraindividual variability on repeated measurements, sST2 could be a better biomarker of HF than NT-proBNP. The study by Castiglione et al shows a higher cutoff of 35 ng/mL for risk stratification in acute HF [11]. This is much higher than the cut-off of the present study probably due to variations in the ethnicity and the race of the study population. sST2 and NT-proBNP levels are significantly higher in HFrEF than in patients with HFmrEF and HFpEF. The present study showed almost the same cut-off as that of the PRIDE study. In the PRIDE study, sST2 concentration of >17.26 ng/mL has a stronger correlation with adverse prognosis in acute HF, and patients with increased sST2 levels show a high risk of

death at one year [43].

In the present study, the ROC curve showed an AUC for Gal-3 of 0.51 with a 95% CI of 0.44 to 0.58; p=0.60. The cut-off value of Gal-3 was 17.58 ng/mL with sensitivity and specificity of 55% and 57% respectively. (Table 5, Fig 3) sST2 and Gal-3 had almost the same diagnostic potential and seemed to be better than that of NT-proBNP in various aspects. Thus, in HF, the diagnostic threshold of sST2, Gal-3, and NT-proBNP may be combined for better diagnosis and risk stratification of HF especially HFpEF. According to Jiang et al, the cut-off value of Gal-3 to diagnose HF is 17.8 ng/mL. Gal-3 is higher in patients with HFpEF than that in HFrEF. Gal-3 levels are associated with ECHO findings, which further substantiates that Gal-3 is useful in diagnosing HFpEF [1]. Two studies show the same Gal-3 cut-off values as that of the present study. In the DEAL study, after adjusting for confounders, Gal-3 remains as the sole predictor with the cut-off value of 17.6ng/mL. In the COACH study (Coordinating Study Evaluating Outcomes of Advising and Counseling in Heart Failure), Gal-3 values >17.8 ng/mL are related to an increased risk of new hospitalization for HF. On the contrary, in the HF-ACTION cohort, which includes ambulatory HF patients who underwent a structured exercise program, the median Gal-3 level is 14.0 ng/mL, which is lower than the present study [60].

In the present study, the ROC curve showed an AUC for microRNA210-3p of 0.79 with a 95% CI of 0.68 to 0.89 with p=<0.001. The cut-off fold change expression was 5.03 with sensitivity and specificity of 87% and 54% respectively. MicroRNA210-3p had the highest sensitivity and AUC among the biomarkers analyzed in the present study. micro-210-3p could be an early diagnostic marker of HFpEF. The microRNA detection methods should ensure accuracy and precision [61]. MAGGIC score demonstrated the overall best performance with the highest AUC of 0.86 (95% CI: 0.86-0.90) (p=0.001, a cut-off score of 14 with sensitivity of 95% and specificity of 89%, suggesting its reliability for risk stratification. (Table 5, Fig 3)

Widely accepted MAGGIC score showed a positive correlation with NT-proBNP, sST2, and galectin-3. There was a positive correlation among NT-proBNP, sST2, and galectin-3. But microRNA did not show any correlation with any of the variables probably due to the small sample size. NT-proBNP and MAGGIC scores showed a positive correlation with HbA1c. sST2 showed a positive correlation with total cholesterol and HbA1c. Total cholesterol showed a positive correlation with triglyceride and LDL cholesterol and a negative correlation with HDL cholesterol (Table 6). Jiang et al, concluded that in HF, BNP is sensitive to volume overload, while Gal-3 is sensitive to fibrosis, hence a combination of these markers may be beneficial in diagnosis [1]. Patients with elevated levels of both NPs and Gal-3 have more than twice the risk of developing HF [60]. Cui et al, have shown that Gal-3 is superior to sST2 in distinguishing HFpEF from healthy individuals and other phenotypes of HF. However, sST2 provides incremental value to NT-proBNP in HF. Although AHA has recommended Gal-3 and sST2 to be used along with NPs for risk stratification in HF, only a few data are available concerning their prognostic value in HFpEF [60]. Since Gal-3 levels are directly related to LV end-diastolic pressure in HFpEF, elevated levels are associated with frequent hospitalizations and increased mortality [62]. Only Gal-3 maintains strong prognostic ability after correction for clinical factors and NPs [38]. Similar to NPs, Gal-3 is also limited by CKD with GFR <60 ml/min/1·73m$^2$. Gal-3 is useful in predicting adverse events in HFpEF, whereas sST2 is useful in HFrEF. Serum sST2 measurements provide information on myocardial fibrosis and systolic dysfunction [62]. The combination of NT-proBNP and sST2 gives an insight into NYHA states. sST2 is relatively better than NT-proBNP in the detection of HFpEF in hypertensive patients [58]. Gal-3 has few advantages over the other two markers, in that it predicts HF risk in the general population as well as in the early fibrosis and ventricular remodeling in HF [63].

The pathogenesis of HF is highly complex, hence relying on a single biomarker will not be sufficient in diagnosing heart failure. Each biomarker has its inherent advantages and

disadvantages. Thus, a multi-biomarker strategy, such that biomarkers could provide information on different pathophysiological pathways, may offer a promising strategy that goes beyond the limits of the current management of HF [64]. In community-based screening, ECHO may be supplemented with NT-proBNP and sST2 and Gal-3 may be done in addition to stratifying the patients for effective management [65]. Also, it enhances the possibility of employing tailored therapies for individuals with heterogeneous presentations. To date, the biomarkers chosen for research are selected at random and by varied experiences of the researcher. A computer-assisted approach can be used as an alternative to select the panel of biomarkers. ACC/AHA guidelines suggest that a combination of biomarkers is beneficial for risk stratification. It is better to have an integrated protocol of biomarkers with clinical variables and imaging characteristics [11].

### Strengths:

The study was carried out in India, where the resource settings are limited. India as such is highly diverse in economy, food, physical habits, and literacy rate across the various states. However, the study was carried out in a tertiary care hospital in a Metropolitan city. To a major extent, it takes into account the various factors. A multi-marker study in a relatively large sample size comprising both genders remains be major strength of the study.

### Limitations:

The study did not include healthy individuals and hence, baseline levels of healthy individuals could not be established. Also, the influence of metabolic diseases- hypertension, diabetes mellitus, and obesity on HFpEF could not be established. A cohort of hypertensive and diabetes mellitus patients could establish the cause-and-effect relationship and a better profile of markers according to the stage of the study.

## Conclusion

The biomarkers- NT-proBNP, sST2, and galectin-3 were markedly elevated in HF patients with EF less than 50% than in patients with more than 50%. Since HF is a multi-organ syndrome, other organs could also contribute to increased galectin-3 and sST2 levels in HF. The gold-standard marker- NT-proBNP showed a positive association with both sST2 and galectin-3. Also, there was a positive association of sST2 with galectin-3. microRNA-210-3p fold changes were positively associated with galectin-3, indicating that both the markers indicate the cardiac fibrosis stage of HF. Since each marker has its specific advantages and disadvantages, a multi-marker approach could help in the early diagnosis as well as in stratifying HF patients so that appropriate target therapies can be implemented according to the pathogenic stage of the disease.

# List of abbreviations

ACE: angiotensin-converting enzyme

AHA/ACC: American Heart Association/ American College of Cardiology

AMI: acute myocardial infarction

AUC: area under the curve

BMI: body mass index

BNP: brain natriuretic peptide

CKD: chronic kidney disease

COACH study: Coordinating Study Evaluating Outcomes of Advising and Counseling in Heart Failure

CSI-KHFR: Cardiology Society of India-Kerala Acute Heart Failure Registry

CT: cycle threshold

CV: coefficient of variation

CVD: cardiovascular disease

ECG: electrocardiogram

ECHO: echocardiography

EF: ejection fraction

ELISA: enzyme-linked immunosorbent assay

ESC: European Society of Cardiology

FC: fold change

Gal-3: Galectin-3

GFR: glomerular filtration rate

HbA1c: glycated hemoglobin A1c

HDL: high-density lipoprotein

HF: heart failure

HFmrEF: heart failure with mid-range or mildly reduced ejection fraction

HFpEF: heart failure with preserved ejection fraction

HFrEF: heart failure with reduced ejection fraction

HIF-1α: hypoxia-inducible factor- 1 alpha

IL-33: interleukin-33

JNC: Joint National Committee

LDL: low-density lipoprotein

LV: left ventricle

LVH: left ventricle hypertrophy

MAGGIC: Meta-Analysis Global Group in Chronic Heart Failure

MAPK: mitogen-activated protein kinase

miRNA: microRNA

NF-κB: nuclear factor – kappa beta

NP: natriuretic peptides

NT-proBNP: N-terminal pro-brain natriuretic peptide

NYHA: New York Heart Association

qRT-PCR: quantitative reverse transcription- polymerase chain reaction

RAAS: renin–angiotensin–aldosterone system

RNA: ribonucleic acid

ROC: receiver operating characteristics

RV: right ventricle

SCD: sudden cardiac death

sST2: soluble suppression of tumorigenicity 2

T2DM: type 2 diabetes mellitus

TGL: triglyceride

WHR: waist-hip ratio

YLD: years lost due to disability

# Declarations

**Ethics approval and consent to participate**: Institutional ethics committee approval was obtained from the institutional ethics committee. Ethics number: IEC-NI/19/FEB/68/09, dated 10.11.2020. Written informed consent was obtained from all the study participants.

**Consent for publication**: Not applicable

**Availability of data and materials**: The authors confirm that the data supporting the findings of this study are available within the article. Raw data were generated at the location of research, Sri Ramachandra Institute of Higher Education and Research, Chennai, India. No supplementary material is available with this research.

**Conflict of interest:** The authors declare that there are no conflicts of interest.

**Funding declaration**: Ms. Jasmine Chandra A is the PhD scholar who received Founder-Chancellor Sri N.P.V. Ramasamy Udayar Research Fellowship (U02B180535), from Sri Ramachandra University, renamed as Sri Ramachandra Institute of Higher Education and Research (Deemed to be University) for the Full-Time PhD program for a period of three years from October 2018 to September 2021.

## Authors contributions:

All the authors contributed equally in conceptualization, data curation, formal analysis, funding acquisition, investigation, methodology, project administration, resources, software, supervision, validation, visualization, writing – original draft, and writing– review & editing.

**Acknowledgments:** The authors wish to thank the Department of Cardiology and the patients for the effective completion of the study.

# References

1. Jiang J, Yang B, Sun Y, Jin J, Zhao Z, Chen S. Diagnostic value of serum concentration of galectin-3 in patients with heart failure with preserved ejection fraction. Front Cardiovasc Med. 2022;8:829151.
2. Lippi G, Sanchis-Gomar F. Global epidemiology and future trends of heart failure. AME medical journal. 2020;5.
3. Shrivastava A, Haase T, Zeller T, Schulte C. Biomarkers for heart failure prognosis: proteins, genetic scores, and non-coding RNAs. Frontiers in cardiovascular medicine. 2020;7:601364.
4. Guo Y, Lip GY, Banerjee A. Heart failure in East Asia. Curr Cardiol Rev. 2013;9(2):112-22.
5. Huffman MD, Prabhakaran D. Heart failure: epidemiology and prevention in India. Natl Med J India. 2010;23(5):283-8.
6. Ponikowski P, Voors AA, Anker SD, Bueno H, Cleland JGF, Coats AJS, et al. ESC Scientific Document Group. 2016 ESC Guidelines for the diagnosis and treatment of acute and chronic Heart failure: The Task Force for the Diagnosis and treatment of acute and chronic heart failure of the European Society of Cardiology (ESC) Developed with the special contribution of the Heart Failure Association (HFA) of the ESC. Eur Heart J. 2016;37(27):2129-2200.
7. McDonagh TA, Metra M, Adamo M, Gardner RS, Baumbach A, Böhm M, et al. 2021 ESC Guidelines for the diagnosis and treatment of acute and chronic heart failure: Developed by the Task Force for the diagnosis and treatment of acute and chronic heart failure of the European Society of Cardiology (ESC) With the special contribution of the Heart Failure Association (HFA) of the ESC. European heart journal. 2021;42(36):3599-726.

8. Simmonds SJ, Cuijpers I, Heymans S, Jones EAV. Cellular and molecular differences between HFpEF and HFrEF: A step ahead in an improved pathological understanding. Cells. 2020;9(1):242.
9. Mahmood SS, Wang TJ. The epidemiology of congestive heart failure: the Framingham Heart Study perspective. Glob Heart. 2013;8(1):77-82.
10. Savarese G, Lund LH. Global public health burden of heart failure. Card Fail Rev. 2017;3(1):7-11.
11. Castiglione V, Aimo A, Vergaro G, Saccaro L, Passino C, Emdin M. Biomarkers for the diagnosis and management of heart failure. Heart Fail Rev. 2022;27(2):625-43.
12. Dw1orok V, Hähnel V, Bannehr M, Paar V, Edlinger C, Lichtenauer M, et al. soluble suppression of tumorigenicity 2 (sST2) in patients with predominantly decompensated right heart Failure- a prospective observational study. J Clin Med. 2023;12(23):7200.
13. Villacorta H, Maisel AS. Soluble ST2 testing: a promising biomarker in the management of heart failure. Arq Bras Cardiol. 2016;106(2):145-52.
14. Merino-Merino A, Gonzalez-Bernal J, Fernandez-Zoppino D, Saez-Maleta R, Perez-Rivera JA. The role of galectin-3 and ST2 in cardiology: a short review. Biomolecules. 2021;11(8):1167.
15. Gu1an Y, Song X, Sun W, Wang Y, Liu B. Effect of hypoxia-induced microRNA-210 expression on cardiovascular disease and the underlying mechanism. Oxid Med Cell Longev. 2019;2019:4727283.
16. Spinar J, Spinarova L, Malek F, Ludka O, Krejci J, Ostadal P, et al. Prognostic value of NT-proBNP added to clinical parameters to predict two-year prognosis of chronic heart failure patients with mid-range and reduced ejection fraction–A report from FAR NHL perspective registry. PloS one. 2019 Mar 26;14(3): e0214363.

17. Triposkiadis F, Starling RC. Chronic heart failure: diagnosis and management beyond LVEF classification. J Clin Med. 2022;11(6):1718.
18. Chengode S. Left ventricular global systolic function assessment by echocardiography. Ann Card Anaesth. 2016;19(Suppl):S26-34.
19. Karmacharya P, Shrestha GL, Singh S, Shrestha OK. Relation of waist hip ratio and body mass index with the vital capacity. JCMC. 2019; 9(29):51-5.
20. Chobanian AV, Bakris GL, Black HR, Cushman WC, Green LA, Izzo JL Jr, et al. Joint National Committee on prevention, detection, evaluation, and treatment of high blood pressure. National Heart, Lung, and Blood Institute; National High Blood Pressure Education Program Coordinating Committee. Seventh report of the Joint National Committee on prevention, detection, evaluation, and treatment of high blood pressure. Hypertension. 2003;42(6):1206-52.
21. International Expert Committee. International Expert Committee report on the role of the A1C assay in the diagnosis of diabetes. Diabetes Care. 2009;32(7):1327-34.
22. Pocock SJ, Ariti CA, McMurray JJ, Maggioni A, Kober L, Squire IB, Swedberg K, Dobson J, Poppe KK, Whalley GA, Doughty RN. Predicting survival in heart failure: a risk score based on 39 372 patients from 30 studies. *Eur Heart J*. 2013;34:1404–1413.
23. Corteville DC, Bibbins-Domingo K, Wu AH, Ali S, Schiller NB, Whooley MA. N-terminal pro-B-type natriuretic peptide as a diagnostic test for ventricular dysfunction in patients with coronary disease: data from the heart and soul study. Arch Intern Med. 2007;167(5):483-9.
24. Wang Z, Pan X, Xu H, Wu Y, Jia X, Fang Y, et al. Serum soluble ST2 is a valuable prognostic biomarker in patients with acute heart failure. Front Cardiovasc Med. 2022;9:812654.

25. de Boer RA, Lok DJ, Jaarsma T, van der Meer P, Voors AA, Hillege HL, et al. Predictive value of plasma galectin-3 levels in heart failure with reduced and preserved ejection fraction. Ann Med. 2011;43(1):60-8.
26. Available from: https://www.helini.in/uploads/3/0/9/5/30951267/purefast_microrna_mini_spin_prep_kit.pdf
27. Benjamin EJ, Blaha MJ, Chiuve SE, Cushman M, Das SR, Deo R, et al. American Heart Association Statistics Committee and Stroke Statistics Subcommittee. Heart Disease and Stroke Statistics-2017 Update: a report from the American Heart Association. Circulation. 2017;135(10):e146-603.
28. Joseph S, Panniyammakal J, Abdullakutty J, S S, Vaikathuseril L J, Joseph J, et al. The Cardiology Society of India-Kerala Acute Heart Failure Registry: poor adherence to guideline-directed medical therapy. Eur Heart J. 2021:ehab793.
29. Kumar AS, Sinha N. Cardiovascular disease in India: A 360 degree overview. Med J Armed Forces India. 2020;76(1):1-3.
30. Teramoto K, Teng TK, Chandramouli C, Tromp J, Sakata Y, Lam CS. Epidemiology and clinical features of heart failure with preserved ejection fraction. Card Fail Rev. 2022;8:e27.
31. Jarab AS, Al-Qerem W, Hamam H, Abu Heshmeh S, Mukattash TL, Alefishat EA. Factors associated with lipid control in outpatients with heart failure. Front Cardiovasc Med. 2023;10:1153310.
32. Yao YS, Li TD, Zeng ZH. Mechanisms underlying direct actions of hyperlipidemia on myocardium: an updated review. Lipids Health Dis. 2020;19(1):23.
33. Piek A, Du W, de Boer RA, Silljé HHW. Novel heart failure biomarkers: why do we fail to exploit their potential? Crit Rev Clin Lab Sci. 2018;55(4):246-63.

34. Panagopoulou V, Deftereos S, Kossyvakis C, Raisakis K, Giannopoulos G, Bouras G, et al. NT-proBNP: an important biomarker in cardiac diseases. Curr Top Med Chem. 2013;13(2):82-94.

35. Çelik A, Kılıçaslan B, Temizhan A, Güvenç TS, Altay H, Çavuşoğlu Y, et al. How to use natriuretic peptides in patients with heart failure with non-reduced ejection fraction? Anatol J Cardiol. 2023;27(6):308-18.

36. Volpe M, Carnovali M, Mastromarino V. The natriuretic peptides system in the pathophysiology of heart failure: from molecular basis to treatment. Clin Sci (Lond). 2016;130(2):57-77.

37. Kang SH, Park JJ, Choi DJ, Yoon CH, Oh IY, Kang SM, et al. KorHF Registry. Prognostic value of NT-proBNP in heart failure with preserved versus reduced EF. Heart. 2015;101(23):1881-8.

38. Patel AN, Southern WN. BNP-Response to acute heart failure treatment identifies high-risk population. Heart Lung Circ. 2020;29(3):354-60.

39. Ponikowski P, Voors AA, Anker SD, Bueno H, Cleland JGF, Coats AJS, et al. ESC Scientific Document Group. 2016 ESC Guidelines for the diagnosis and treatment of acute and chronic heart failure: The Task Force for the diagnosis and treatment of acute and chronic heart failure of the European Society of Cardiology (ESC)Developed with the special contribution of the Heart Failure Association (HFA) of the ESC. Eur Heart J. 2016;37(27):2129-200.

40. Cao Z, Jia Y, Zhu B. BNP and NT-proBNP as diagnostic biomarkers for cardiac dysfunction in both clinical and forensic medicine. Int J Mol Sci. 2019;20(8):1820.

41. Welsh P, Campbell RT, Mooney L, Kimenai DM, Hayward C, Campbell A, et al. Reference ranges for NT-proBNP (N-terminal pro-B-type natriuretic peptide) and risk

factors for higher NT-proBNP concentrations in a large general population cohort. Circ Heart Fail. 2022;15(10):e009427.

42. Singh S, Pandey A, Neeland IJ. Diagnostic and prognostic considerations for use of natriuretic peptides in obese patients with heart failure. Prog Cardiovasc Dis. 2020;63(5):649-55.
43. Anwaruddin S, Lloyd-Jones DM, Baggish A, Chen A, Krauser D, Tung R, et al. Renal fun4ction, congestive heart failure, and amino-terminal pro-brain natriuretic peptide measurement: results from the ProBNP Investigation of Dyspnea in the Emergency Department (PRIDE) Study. J Am Coll Cardiol. 2006 Jan 3;47(1):91-7.
44. Wettersten N, Horiuchi Y, Maisel A. Advancements in biomarkers for cardiovascular disease: diagnosis, prognosis, and therapy. Fac Rev. 2021;10:34.
45. Lotierzo M, Dupuy AM, Kalmanovich E, Roubille F, Cristol JP. sST2 as a value-added biomarker in heart failure. Clin Chim Acta. 2020;501:120-30.
46. Aimo A, Januzzi JL Jr, Bayes-Genis A, Vergaro G, Sciarrone P, Passino C, et al. Clinical and prognostic significance of sST2 in heart failure: JACC review topic of the week. J Am Coll Cardiol. 2019;74(17):2193-203.
47. Firouzabadi N, Dashti M, Dehshahri A, Bahramali E. Biomarkers of IL-33 and sST2 and lack of association with carvedilol therapy in heart failure. Clin Pharmacol. 2020;12:53-8.
48. Devine PA, Cardwell C, Maxwell AP. Association of soluble ST2 with all-cause and cardiovascular mortality in renal transplant recipients: a single-centre cohort study. BMC Nephrol. 2020;21(1):22.
49. Aleksova A, Paldino A, Beltrami AP, Padoan L, Iacoviello M, Sinagra G, et al. Cardiac biomarkers in the Emergency Department: the role of soluble ST2 (sST2) in acute heart

failure and acute coronary syndrome-there is meat on the bone. J Clin Med. 2019;8(2):270.

50. Bai J, Han L, Liu H. Combined use of high-sensitivity ST2 and NT-proBNP for predicting major adverse cardiovascular events in coronary heart failure. Ann Palliat Med. 2020;9(4):1976-89.
51. Janjusevic M, Fluca AL, Ferro F, Gagno G, D'Alessandra Y, Beltrami AP, et al. Traditional and emerging biomarkers in asymptomatic left ventricular dysfunction-promising non-coding RNAs and exosomes as biomarkers in early phases of cardiac damage. Int J Mol Sci. 2021;22(9):4937.
52. Blanda V, Bracale UM, Di Taranto MD, Fortunato G. Galectin-3 in cardiovascular diseases. Int J Mol Sci. 2020;21(23):9232.
53. Gehlken C, Suthahar N, Meijers WC, de Boer RA. Galectin-3 in heart failure: an update of the last 3 years. Heart Fail Clin. 2018;14(1):75-92.
54. Amin HZ, Amin LZ, Wijaya IP. Galectin-3: a novel biomarker for the prognosis of heart failure. Clujul Med. 2017;90(2):129-32.
55. Zaborska B, Sikora-Frąc M, Smarż K, Pilichowska-Paszkiet E, Budaj A, Sitkiewicz D, et al. The role of galectin-3 in heart failure-the diagnostic, prognostic and therapeutic potential-where do we stand? Int J Mol Sci. 2023;24(17):13111.
56. Zhou SS, Jin JP, Wang JQ, Zhang ZG, Freedman JH, Zheng Y, et al. miRNAS in cardiovascular diseases: potential biomarkers, therapeutic targets and challenges. Acta Pharmacol Sin. 2018;39(7):1073-84.
57. Shen NN, Wang JL, Fu YP. The microRNA expression profiling in heart failure: a systematic review and meta-analysis. Front Cardiovasc Med. 2022;9:856358.

58. Felker GM, Fiuzat M, Shaw LK, Clare R, Whellan DJ, Bettari L, et al. Galectin-3 in ambulatory patients with heart failure: results from the HF-ACTION study. Circ Heart Fail. 2012;5(1):72-8.
59. Yancy CW, Jessup M, Bozkurt B, Butler J, Casey DE Jr, Drazner MH, et al. American College of Cardiology Foundation/American Heart Association Task Force on Practice Guidelines. 2013 ACCF/AHA guideline for the management of heart failure: a report of the American College of Cardiology Foundation/American Heart Association Task Force on practice guidelines. Circulation. 2013;128(16):e240-327.
60. Shi Y, Liu J, Liu C, Shuang X, Yang C, Qiao W, et al. Diagnostic and prognostic value of serum soluble suppression of tumorigenicity-2 in heart failure with preserved ejection fraction: a systematic review and meta-analysis. Front Cardiovasc Med. 2022;9:937291.
61. Galluzzo A, Gallo S, Pardini B, Birolo G, Fariselli P, Boretto P, et al. Identification of novel circulating microRNAs in advanced heart failure by next-generation sequencing. ESC Heart Fail. 2021;8(4):2907-19.
62. Cui Y, Qi X, Huang A, Li J, Hou W, Liu K. Differential and predictive value of galectin-3 and soluble suppression of tumorigenicity-2 (sST2) in heart failure with preserved ejection fraction. Med Sci Monit. 2018;24:5139-46.
63. Rincón LM, Rodríguez-Serrano M, Conde E, Lanza VF, Sanmartín M, González-Portilla P, et al. Serum microRNAs are key predictors of long-term heart failure and cardiovascular death after myocardial infarction. ESC Heart Fail. 2022;9(5):3367-79.
64. Salzano A, Marra AM, D'Assante R, Arcopinto M, Bossone E, Suzuki T, Cittadini A. Biomarkers and Imaging: Complementary or Subtractive? Heart Fail Clin. 2019 Apr;15(2):321-331.

65. Salzano A, D'Assante R, Israr MZ, Eltayeb M, D'Agostino A, Bernieh D, De Luca M, Rega S, Ranieri B, Mauro C, Bossone E, Squire IB, Suzuki T, Marra AM. Biomarkers in Heart Failure: Clinical Insights. Heart Fail Clin. 2021 Apr;17(2):223-243.

Figure

Click here to access/download;Figure;Fig 3 ROC.tiff

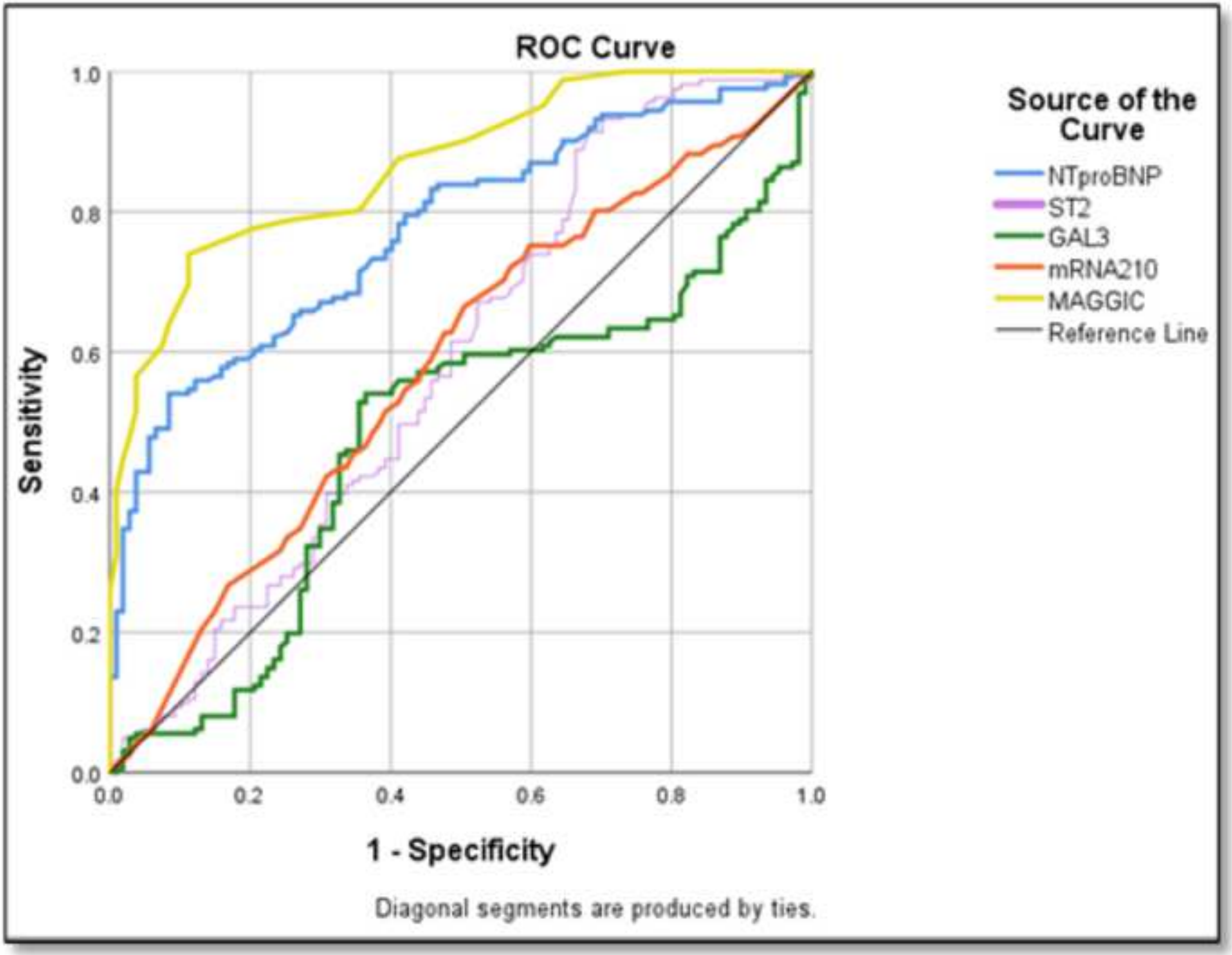

Click here to access/download;Figure;Flow chart Fig 1.tiff

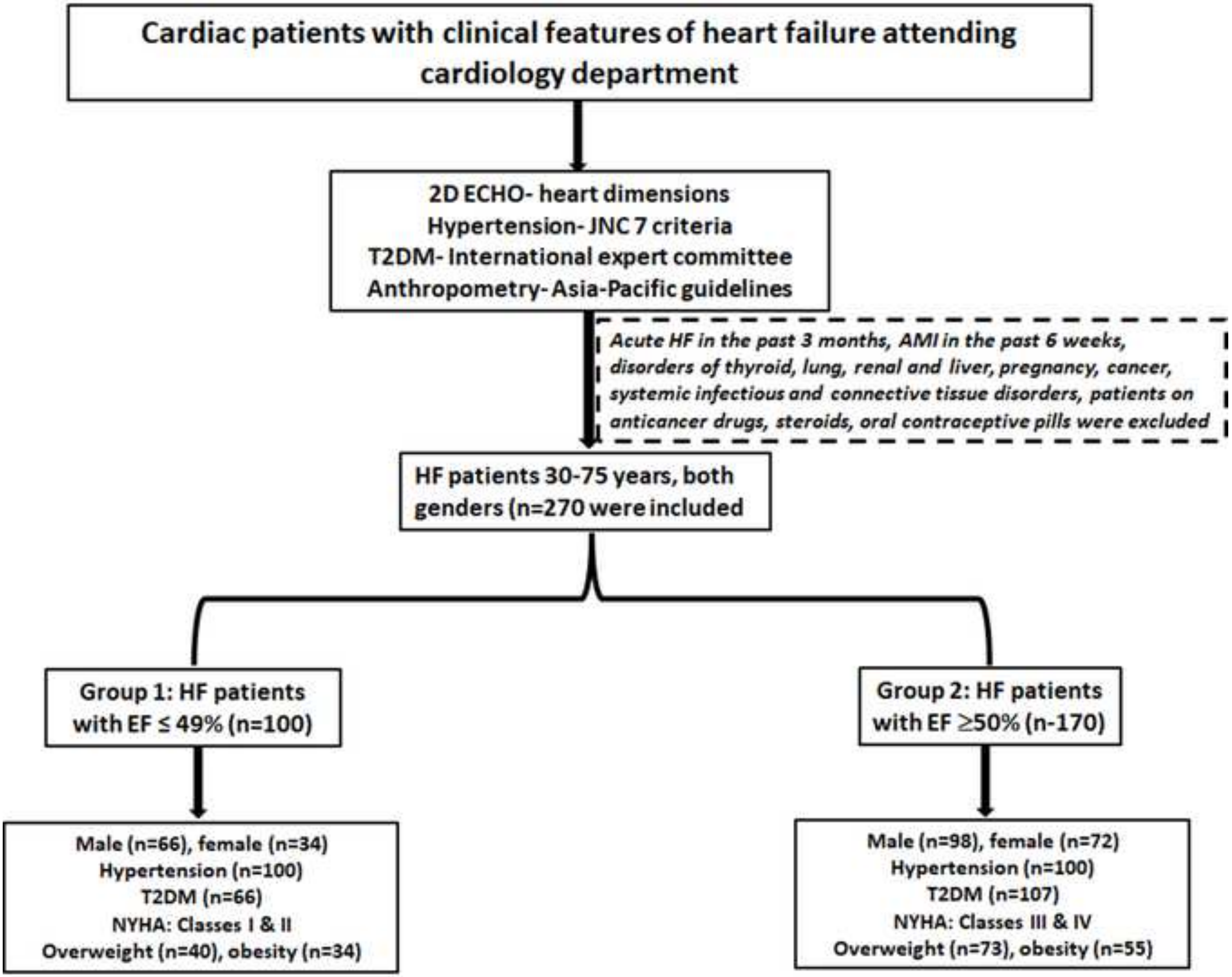

Figure

Click here to access/download;Figure;Fig 2A amplication pattern in group 1.tiff

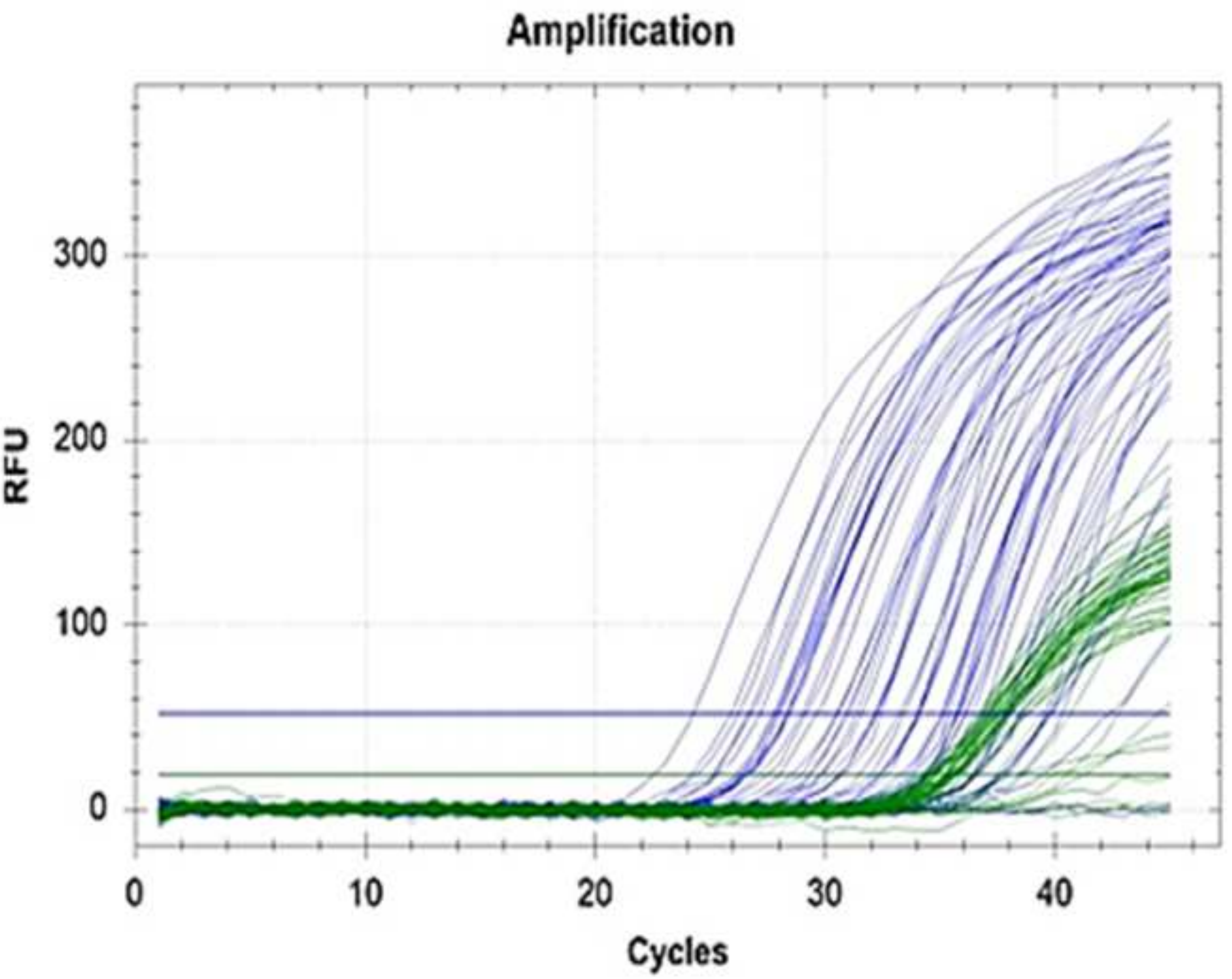

Click here to access/download;Figure;Fig 2B amplification pattern in group 2.tiff

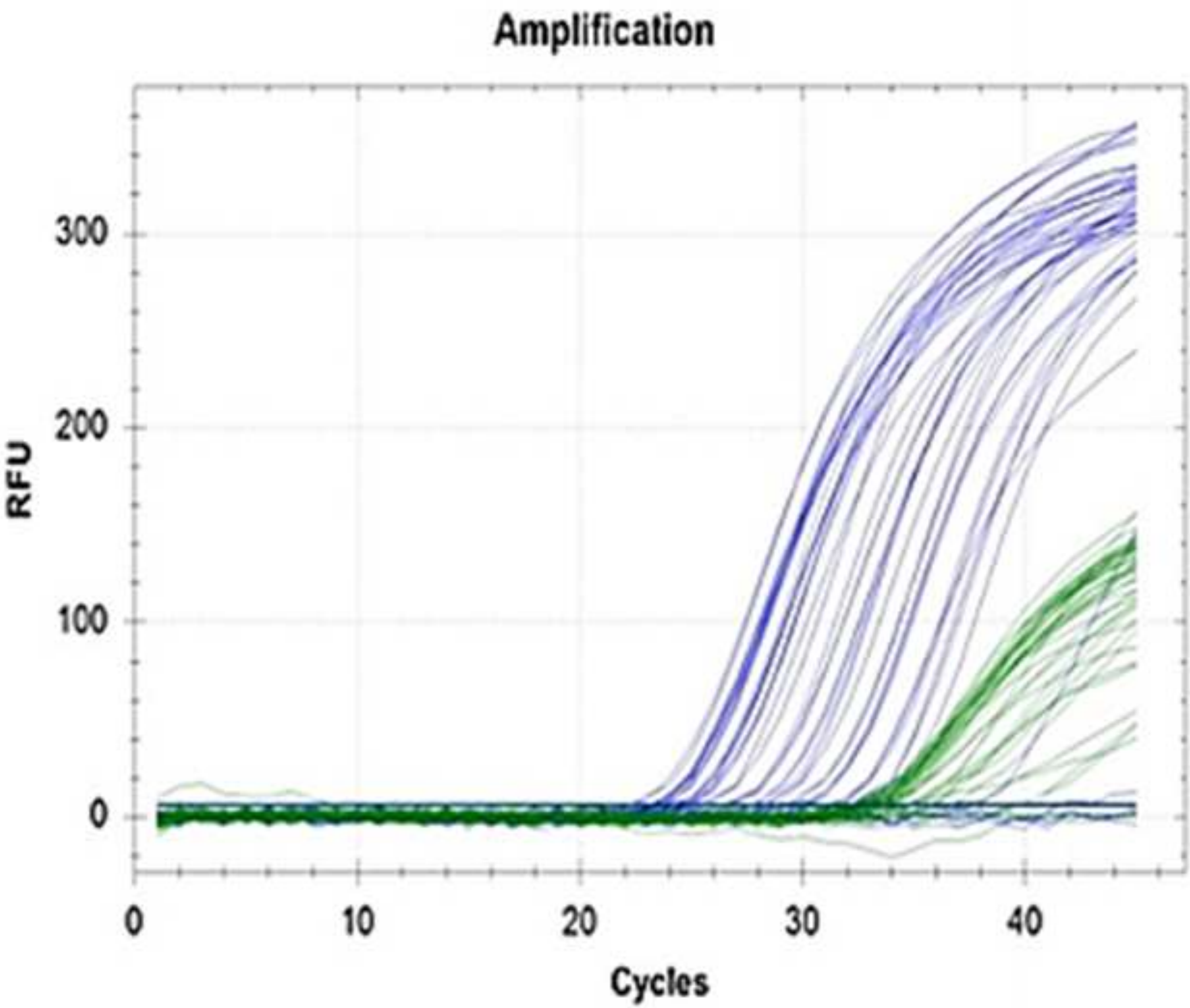

Click here to access/download
**Supporting Information**
Data set Plos one.xlsx

**Original Research**

**Title page**

# Association of NT-proBNP, sST2, Galectin-3 and microRNA-210-3p in heart failure patients with preserved and reduced ejection fraction- cross-sectional study

Jasmine Chandra A[1], Sudagar Singh RB[2], Mohanalakshmi P[3], Mahesh Kumar K[4], Karthikeyan Rajamani[5], **Santhi Silambanan**[6*]

[1] Department of Biochemistry, Sri Ramachandra Institute of Higher Education and Research, Chennai, India.

[2] Department General Medicine, Sri Ramachandra Institute of Higher Education and Research, Chennai, India.

[3] Department of Biochemistry, Sri Muthukumaran Medical College Hospital and Research Institute, Chennai, India.

[4] Department of Physiology and Biochemistry, Government Yoga and Naturopathy Medical College and Hospital, Chennai, India.

[5] Department of Public Health, Sri Ramachandra Institute of Higher Education and Research, Chennai, India.

[6*] Department of Biochemistry, Sri Ramachandra Institute of Higher Education and Research, Chennai, India.

**Address for communication of corresponding author**:

Dr Santhi Silambanan

email: santhisilambanan@sriramachandra.edu.in, santhisilambanan@gmail.com

# Association of NT-proBNP, sST2, Galectin-3 and microRNA-210-3p in heart failure patients with preserved and reduced ejection fraction- cross-sectional study


## Abstract

## Background

Hear failure (HF) is a growing health problem and around two percentage are affected in the general population. The prevalence seems to be increasing due to improved management of communicable and noncommunicable diseases. Accurate diagnostic markers which have the potential in early diagnosis of HF is lacking. The aim of this study was to compare the levels of biomarkers -sST2, galectin-3 and microRNA-210-3p with the gold standard marker- NT-proBNP in heart failure patients with preserved and reduced ejection fractions.

## Materials and Methods

The cross-sectional study was conducted in 270 hypertensive heart failure patients in the age group of 30 to 75 years of both genders. The participants ~~hypertensive patients~~ with evidence of HF were recruited from the Department of Cardiology in a tertiary care hospital in Chennai, India . ~~It was conducted from December 2020 to November 2022~~. ~~The hypertensive patients with evidence of HF were recruited from the Department of Cardiology in a tertiary care hospital in Chennai, India.~~ ~~Among the study participants, 50% of them had diabetes mellitus along with hypertesion.~~ Blood~~Serum~~ samples were obtained for analysis of~~;~~ NT-proBNP, sST2, and galectin-3 were measured by ELISA. For microRNA-210-3p analysis, 20 apparently healthy individuals were included. MicroRNA~~-210-3p~~ was analyzed by qRT-PCR. Institutional ethics committee approval and written informed consent were obtained. Statistical


**Commented [SS1]:** Modified methodology

**Commented [SS2]:** Reframmed

analysis was performed using R software version 4.2.1. The obtained data were subjected to normality of distribution. Based on the type of distribution, appropriate statistical tools were used to analyze the obtained data. p value ≤ 0.05 was considered to be statistically significant.

## Results

NT-proBNP, sST2, ~~and~~ galectin-3 and microRNA-210-3p were significantly higher in HFrEF compared to that of HFpEF and ~~.~~ showed correlation among the markers. MAGGIC score showed positive correlation with all the biomarkers. The cut-off of MAGGIC score was 14, miRNA-210-3p was s 5.03, NT-proBNP was 355.5 and sST2 was 20.11. However, galectin-3 appeared to have limited value, with an AUC of 0.51 and a non-significant p value of 0.60.~~Also, microRNA-210-3p showed significantly increased expression in HFpEF compared to controls. The fold changes were much higher in HFrEF compared to HFrEF.~~

## Conclusion

~~NT-proBNP, sST2 and galectin-3 were significantly elevated in HFrEF. NT-proBNP showed positive association with both sST2 and galectin-3. Also, there was positive association between sST2 and Gal-3. MicroRNA-210-3p showed marked increased expression in both the groups compared to controls. microRNA-210-3p fold changes were positively associated with galectin-3.~~ All the biomarkers are significantly elevated in HFrEF compared to HFpEF, hence, the biomarkers are able to differentiate the various HF cases. MicroRNA-210-3p could be an early marker of diagnosing heart failure patients. The strategy of employing multi-marker approach could help in the early diagnosis as well as in stratifying the HF patients~~.~~. This could aid in implementing targeted therapies.

**Commented [SS3]:** Results modified

## Key words

Hear failure, ejection fraction, NT-proBNP, sST2, galectin-3~~2~~, microRNA-210-3p

## Introduction

Heart failure (HF) is a complex multisystem disorder due to altered structural and functional alterations in the heart [1]. It affects 64.34 million people, leading to 9.91 million years lost due to disability (YLDs). In the US, HF prevalence has been projected to increase from 2.42% in 2012 to 2.97% in 2030 [2]. HF is emerging as a health burden in Asia as well, with its prevalence ranging from 1.3 and 6.7%, which is considered to be higher than that of Western countries [3]. Among the Asian countries, the prevalences are 1.3%, 1.0%, 6.7% and 4.5% in China, Japan, Malaysia and Singapore respectively [4]. In India, the number of new HF cases may increase from 0.118 ~~—~~-0.708 million in 2000 to 0.214 ~~—~~-1.3 million in 2025 [5].

HF patients are grouped into three phenotypes based the ejection fraction (EF) of the left ventricle (LV) as measured by echocardiography (ECHO). HF is categorized as HF with reduced EF (HFrEF) with EF<40%, HF with preserved EF (HFpEF) with EF>50% and HF with mid-range/ mildly reduced EF (EFmrEF) with EF 41-49% [6]. ~~The commonly occurring and well characterized~~ HFrEF is preceded by various cardiovascular diseases (CVD) especially acute myocardial infarction (AMI), valvular and other cardiac diseases [7]. ~~In HFpEF, patients develop LV dysfunction and clinical features of HF, even before the EF drops to less than 50% [1].~~ Hypertension is the most important cause of HFpEF, with a prevalence of 60% to 89% among HF patients [7]. HFpEF is prevalent ~~in~~ among older population especially women. Apoptosis and autophagy cause concentric hypertrophy and LV wall stiffening. The prevalence of HFrEF has been declining, while HFpEF is increasing [8]. Till date, HF is diagnosed by ECHO examination and measurement of serum natriuretic peptides (NPs) levels. Based on these investigations, the chances of missing the early-stage HF could be high especially when EF is more than 50% as in HFpEF [7].

The diagnosis of HF is based on the Framingham Diagnostic Criteria for HF [9]. ~~HF is unlikely in patients presenting with a completely normal electrocardiogram (ECG). However, accurate report is highly dependent on the imaging modality and quality, and operator efficiency [7].~~

Diagnosis and management of HFrEF has been studied in detail and have been implemented effectively in practice [8]. But, HFpEF and HFmrEF are poorly investigated and managed, particularly in the developing countries [10]. Since the~~ pathogenesis involved in HF development and progression is complex, biomarkers are gaining importance. B~~ biomarkers have the potential to indicate the pathophysiology of the disease process ~~. Hence, biomarkers may be used as diagnostic and prognostic tools in HF and also, they can discriminate recovery from complete remission ~~[11]. European Society of Cardiology (ESC) guidelines (2016) recommends that patients suspected to have acute HF should have their plasma NPs tested [7]. American Heart Association/ American College of Cardiology (AHA/ACC) guidelines on the management of HF recommends the measurement of soluble suppression of tumorigenicity 2 (sST2) in patients with acute HF for a more appropriate stratification [12]. sST2 levels at the time of admission is more valuable than NPs in assessing the risk of mortality at one year after admission for acute HF [13]. Galectin-3 (Gal-3) is ~~a beta galactoside which is ~~involved in inflammation, repair, and fibrosis. Thus, in HF, Gal-3 is a ~~superior ~~predictor of cardiovascular mortality and the frequency of hospital admissions [14]. MicroRNA-210-3p is upregulated in tissue hypoxic conditions and thus regulate cell differentiation, proliferation, migration, apoptosis, mitochondrial metabolism, and angiogenesis [15].

~~Fibrosis and scar formation in the LV of heart indicates the maladaptive ventricular responses to injury. Since the heart has limited regenerative capacity after injury, repair processes replace necrotic cardiomyocytes with fibrotic scar tissue [8]. There is an urgent need for identifying novel biomarkers which may have screening, diagnostic and prognostic potential in HF [15].~~

Since the prevalence of metabolic disorders such as hypertension, diabetes mellitus and obesity obesity are high and the chances for them to develop cardiac problems is also high. Studies on application of biomarkers in diagnosis and progrnosis of HF are limited. In poor economic settings, HF imposes huge mortality and mortality, thus increasing the burden to the society.

**Commented [SS4]:** Added informationzzz

The aim of this study was to determine the association of sST2, Gal-3 and microRNA 210-3p with the gold standard marker- NT-proBNP in heart failure patients with preserved and reduced ejection fractions.

# Materials and Methods

The prospective cross-sectional study was conducted among 270 HF patients of both genders in the age group of 30 to 75 years. Sample size was calculated based on the study by Spinar et al. 2019 [16]. Hypertensive patients with evidence of HF were recruited from the Department of Cardiology at Sri Ramachandra Institute of Higher Education and Research, Chennai, India from 01-12-2020 to 15-02-2023. Initially the study was decided to conduct only on hypertensive patients with HF. Due the COVID-19 pandemic, ~~obtaining~~recruiting exclusive hypertensive patients was highly challenging. Hence, with the decision of the research advisory committee members, it was decided to include hypertensive HF patients who also had diabetes mellitus; and revised ethics approval was obtained. Thus, among the 270 patients, 168~~35~~ patients had diabetes mellitus in addition to hypertension. Diagnosis of HF was based on Framingham Heart Failure Diagnostic criteria [9].

The study participants were subjected to the transthoracic 2D doppler echocardiography using Phillips and GE Healthcare echocardiography with patients lying supine in the left lateral decubitus position. According to the definition of European and United States guidelines, the normal EF is 52-72% in men and 54-74% in women [~~21,~~17]. According to Chengode, EF > 55% is considered to be normal. It can be 45%–54%, 30%–44% and < 30% in mild, moderate and severe LV dysfunction respectively [~~22,~~ 18]. Anthropometric characteristics such as body mass index (BMI) in $Kg/m^2$ and waist to hip ratio (WHR) were measured as per the Asia-Pacific guidelines [~~23~~ 19]. Systolic and diastolic blood pressures were measured using standard procedure and patients were identified as hypertensives based on the JNC7 blood pressure classification [~~24~~ 20]. According to International Expert Committee, diabetes mellitus was

**Commented [SS5R4]:** Edited and removed extra information in introduction

**Formatted:** Strikethrough

**Formatted:** Strikethrough

**Formatted:** Strikethrough

**Formatted:** Strikethrough

diagnosed based on glycated hemoglobin (HbA1c) ≥6.5%, fasting plasma glucose ≥126 mg/dL and 2-hour plasma glucose or random plasma glucose ≥200 mg/dL [~~25~~ 21].

Formatted: Strikethrough

LV ejection fraction (EF) remains the major parameter for phenotyping, diagnosis, prognosis and treatment decisions in HF. In the present study, EF of 50% was used as the cut-off to categorize the participants into two groups as group 1 – HF with EF ≤ 49% and group 2 -HF with EF ≥ 50%. As per the 2016- ESC Guidelines, EF of 40- 49% is categorized as HFmrEF. HFmrEF is an intermediate clinical entity between HFrEF and HFpEF, but more similar to HFrEF with regard to the high prevalence of AMI in these patients [7]. Patients with HFmrEF are similar to HFrEF in being younger males, increased systolic blood pressure and reduced LV and atrial dilatation. Also, HFmrEF are found to benefit from HFrEF treatment strategies [7]. In the present study, based on the above literature data, HFmrEF has been included in HFrEF.

The participants were grouped into group 1 with HF patients with EF ≤ 49% and group 2 with EF ≥50%. Patients with acute HF in the past 3 months, AMI in the past 6 weeks, disorders of thyroid, lung, renal and liver, pregnancy, cancer, systemic infectious diseases, connective tissue disorders, and patients on anticancer drugs, steroids, and oral contraceptive pills were excluded from the study. The Meta-Analysis Global Group in Chronic Heart Failure (MAGGIC) mortality risk score was calculated in all the study participants. The MAGGIC score consists of thirteen clinical predictor variables such as age, sex, body mass index (BMI), systolic blood pressure (SBP), ejection fraction (EF), serum creatinine level, participant is a current smoker, has diabetes mellitus, and chronic obstructive pulmonary disease, belongs to New York Heart Association (NYHA) class, heart failure (HF) diagnosed for at least eighteen months, use of beta-blockers and angiotensin-converting enzyme (ACE) inhibitors.

**Commented [SS6]:** Removed in results and added in methodology

**Formatted:** Font color: Red

**Commented [SS7]:** Information regarding MAGGIC score has been added. New reference ha been added.

NT-pro BNP, sST2, and Gal-3 were measured by ELISA:

- NT-proBNP: measurement range: 7.5 - 120 pg/mL, ~~sensitivity~~sensitivity: 0.5 pg/mL, CV%: intra-assay < 9% and inter-assay < 11%; Biological Reference Interval: ≤74 years: < 125 pg/mL, and ≥ 75 years: < 450 pg/mL [17].
- sST2: measurement range: 0.31-20.0 ng/mL, sensitivity: 0.19 ng/mL, CV%: intra-assay 5.4% and inter-assay 6.1%, Biological Reference Interval: 14.42 ng/mL [18].
- Gal-3: measurement range: 0.16-10.0 ng/mL, sensitivity: 0.16ng/mL, CV%: intra-assay 5.56% and inter-assay 7.27%, Biological Reference Interval: < 17.8 ng/mL [19].

MicroRNA-210-3p was analyzed by qRT-PCR. MicroRNA 210-3p was expressed as cycle threshold folds. Analysis of microRNA-210-3p: Methodology: RT-PCR, microRNA was isolated by PureFast® miRNA mini spin purification kit [20].

>hsa-miR-210-3p MIMAT0000267; CUGUGCGUGUGACAGCGGCUGA

Analysis was done on 100 samples; with 20 controls (apparently healthy individuals without hypertension or diabetes mellitus or HF), 40 patients of group 1 and 40 patients of group 2. Fold change was calculated as follows:

$$\Delta CT = CT - CT_0$$

$$\Delta\Delta CT = \Delta CT - \Delta CT_{control}$$

$$FC = 2^{-\Delta\Delta CT}$$ (CT: cycle threshold, FC: fold change)

## Ethics Statement

Institutional ethics committee approval was obtained from institutional ethics committee. Ethics number: IEC-NI/19/FEB/68/09, dated 10.11.2020. Written informed consent was obtained from all the study participants.

## Statistics used

Statistical analysis was performed using R software version 4.2.1. The obtained data were subjected to Kolmogorov-Smirnov test to check for normality of distribution. Continuous variables were expressed as mean and standard deviation or median and interquartile range and

student 't' test or Mann-Whitney U test was used to compare the data between the groups. Categorical variables were expressed as frequency and percentage; Chi-Square test or Fischer-exact test was used to compare the data. Pearson correlation coefficient was used to test correlation among the variables. Receiver operating characteristics (ROC) curve was done to find the area under the curve (AUC). Youden index was used to find the cut-off values of biomarkers. p value ≤0.05 was considered statistically significant.

Large Language Models (LLMs), such as ChatGPT were not used by any of the authors in this manuscript.

# Results

The study participants were subjected to the transthoracic 2D doppler echocardiography using Phillips and GE Healthcare echocardiography with patients lying supine in the left lateral decubitus position. EF values in healthy individuals and in heart failure patients vary with ethnicity and type of ECHO instrument. According to the definition of European and United States guidelines, the normal EF is 52-72% in men and 54-74% in women [21]. According to Chengode, EF > 55% is considered to be normal. It can be 45%–54%, 30%–44% and < 30% in mild, moderate and severe LV dysfunction respectively [22].

Anthropometric characteristics such as body mass index (BMI) in $Kg/m^2$ and waist to hip ratio (WHR) were measured as per the Asia-Pacific guidelines [23]. Systolic and diastolic blood pressureswere measured using standard procedure and patients were identified as hypertensives based on the JNC7 blood pressure classification [24]. According to International Expert Committee, diabetes mellitus was diagnosed based on glycated hemoglobin (HbA1c) ≥6.5%, fasting plasma glucose ≥126 mg/dL and 2-hour plasma glucose or random plasma glucose ≥200 mg/dL [25].

LV ejection fraction (EF) remains the major parameter for phenotyping, diagnosis, prognosis and treatment decisions in HF. In the present study, EF of 50% was used as the cut-off to

**Formatted:** Strikethrough

categorize the participants into two groups as group 1 – HF with EF ≤ 49% and group 2 -HF with EF ≥ 50%. As per the 2016- ESC Guidelines, EF of 40- 49% is categorized as HFmrEF. HFmrEF is an intermediate clinical entity between HFrEF and HFpEF, but more similar to HFrEF with regard to the high prevalence of AMI in these patients [7]. Patients with HFmrEF are similar to HFrEF in being younger males, increased systolic blood pressure and reduced LV and atrial dilatation. Also, HFmrEF are found to benefit from HFrEF treatment strategies [7]. In the present study, based on the above literature data, HFmrEF has been included in HFrEF.

Formatted: Strikethrough

Formatted: Strikethrough

The study participants were subjected to the transthoracic 2D doppler echocardiography using Phillips and GE Healthcare echocardiography with patients lying supine in the left lateral decubitus position. EF values in healthy individuals and in heart failure patients vary with ethnicity and type of ECHO instrument. According to the definition of European and United States guidelines, the normal EF is 52-72% in men and 54-74% in women [21]. According to Chengode, EF > 55% is considered to be normal. It can be 45%–54%, 30%–44% and < 30% in mild, moderate and severe LV dysfunction respectively [22].

Anthropometric characteristics such as body mass index (BMI) in $Kg/m^2$ and waist to hip ratio (WHR) were measured as per the Asia Pacific guidelines [23]. Systolic and diastolic blood pressureswere measured using standard procedure and patients were identified as hypertensives based on the JNC7 blood pressure classification [24]. According to International Expert Committee, diabetes mellitus was diagnosed based on glycated hemoglobin (HbA1c) ≥6.5%, fasting plasma glucose ≥126 mg/dL and 2-hour plasma glucose or random plasma glucose ≥200 mg/dL [25].

LV ejection fraction (EF) remains the major parameter for phenotyping, diagnosis, prognosis and treatment decisions in HF. In the present study, EF of 50% was used as the cut-off to categorize the participants into two groups as group 1 – HF with EF ≤ 49% and group 2 -HF

~~with EF ≥ 50%. As per the 2016 ESC Guidelines, EF of 40–49% is categorized as HFmrEF. HFmrEF is an intermediate clinical entity between HFrEF and HFpEF, but more similar to HFrEF with regard to the high prevalence of AMI in these patients [7]. Patients with HFmrEF are similar to HFrEF in being younger males, increased systolic blood pressure and reduced LV and atrial dilatation. Also, HFmrEF are found to benefit from HFrEF treatment strategies [7]. In the present study, based on the above literature data, HFmrEF has been included in HFrEF.~~

Based on the EF cut-off of 50%, 170 patients have EF ≥ 50%, while the remaining 100 HF patients have reduced EF of ≤ 49%. Figure 1 shows the flow chart indicating the recruitment of participants into the study along with patient characteristics. Table 1 shows the demographic data of the study participants.

**Table 1: shows demographic data of the study participants**

| **Characteristics** | **Group 1 (n=100) HF with EF ≤ 49%** | **Group 2 (n=170) HF with EF ≥ 50%** | **p value** |
|---|---|---|---|
| Age (Years) | | | |
| < 45 n (%) | 12 (12~~%~~) | 5 (2~~%~~) | ≤0.~~0~~001** |
| 46-60 n (%) | 26 (26~~%~~) | 118 (70~~%~~) | |
| 61 -75 n (%) | 62 (62~~%~~) | 47 (28~~%~~) | |
| Gender | | | |
| Female n (%) | 34 (34~~%~~) | 72 (42~~%~~) | 0.21 |
| Male n (%) | 66 (66~~%~~) | 98 (58~~%~~) | |
| ~~BMI (Kg/m²)~~ | | | |
| BMI (Kg/m²)~~Normal weight n (%)~~ | ~~26 (26%)~~ | ~~42 (25%)~~ | 0.90~~8~~ |
| Normal weight n (%) | 26 (26) | 42 (25) | |
| Overweight n (%) | 40 (40~~%~~) | 73 (43~~%~~) | |
| Obese n (%) | 34 (34) | 55 (32) | |

Formatted: Strikethrough
Formatted: Strikethrough
Commented [SS8]: Comorbidities have been added
Formatted: Line spacing: 1.5 lines
Formatted Table

| | | | |
|---|---|---|---|
| Hypertension | | | |
| Present n (%) | 100 (100) | 170 (100) | 1.0 |
| Absent n (%) | 0 (0) | 0 (0) | |
| T2DM | | | |
| Present n (%) | 66 (66) | 102 (60) | 0.37 |
| Absent n (%) | 34 (34) | 68 (40) | |
| | | | |
| NYHA | III (59/59) | I (160/94) | <0.001** |
| Class (n/%) | IV (41/41) | II (10/6) | |

*NYHA: New York Heart Association; Expressed as frequency & percentage; Chi-Square test was done; **: p value statistically ~~highly~~ significant*

Among the distribution of age, majority of patients in group 1 were in older age of 61-75 age, whereas among group 2, majority of them were middle aged persons. Most of the participants were males in both the groups. Based on BMI, most of them were in overweight and obese categories compared to normal weight in both the groups. According to NYHA class, participants in group 1 were in NYHA classes III and IV, whereas in group 2, the participants were in the classes I and II. (Table 1) Among the participants, 33% were smokers, 35% were alcoholics, and 46% were vegetarians.

The distribution of biochemical parameters such as lipid profile and the cardiac biomarkers is shown in table 2. ~~Among lipid profile, all the variables were higher among group 1 than in group 2, but statistically significant difference was obtained only for total cholesterol. All cardiac biomarkers showed statistically significant difference between the groups. All the markers were higher in group 1 compared to group 2 (Table 2).~~

**Table 2: Shows levels of biomarkers among the participants**

| ~~**Parameter**~~**Characteristics** | **Group 1 (n=100)** **HF with EF ≤ 49%** | **Group 2 (n=170)** **HF with EF ≥ 50%** | **p value** |
|---|---|---|---|
| ~~Total cholesterol~~TC | 205.29 (46.42) | 193.72 (38.19) | 0.01* |

**Formatted:** Line spacing: 1.5 lines (×8)
**Formatted:** Font: Italic
**Formatted:** Line spacing: single
**Formatted:** Font: Italic, Strikethrough
**Formatted:** Font: Italic
**Commented [SS9]:** Added Table 4 to compare the groups according to age of participants

| | | | |
|---|---|---|---|
| (mg/dL) | | | |
| TG~~L~~ (mg/dL) | 155.80 (63.06) | 149.55 (73.58) | 0.41 |
| HDL (mg/dL) | 43.74 (13.00) | 42.76 (10.38) | 0.92 |
| LDL (mg/dL) | 126.02 (39.78) | 124.28 (33.26) | 0.54 |
| NT-p~~P~~roBNP (pg/mL) | 482.74 (110.94) | 287.8 (81.62) | 0.001** |
| ~~Soluble~~ sST2 (ng/mL) | 38.24 (13.15) | 21.40 (7.42) | 0.001** |
| Gal~~ectin~~-3 (ng/mL) | 24.93 (10.84) | 17.77 (6.03) | 0.001** |
| MiRNA-210-3p (folds) | 11.89 (31.11) | 1.08 (1.36) | 0.03* |
| ~~MicroRNA-210-3p (folds)~~ | ~~11.89 (31.11)~~ | ~~1.08 (1.36)~~ | ~~0.031*~~ |
| MAGGIC score (points) | 22.94 (5.92) | 8.05 (4.64) | 0.001* |

*TC: total cholesterol; TG~~L~~: triglyceride; HDL: high-density lipoprotein, LDL: low-density lipoprotein, NT-proBNP: N-terminal- pro brain natriuretic peptide, sST2: soluble suppression of tumorigenicity, Gal-3: Galectin-3; MiRNA: micro ribonucleic acid; MAGGIC: Meta-Analysis Global Group in Chronic Heart Failure*
*Expressed as mean and SD; Student t test was used; **: p value statistically highly significant*
**: p value statistically significant.*

Among the lipid ~~profiles~~, all the variables were higher among group 1 than in group 2, but statistically significant difference was obtained only for total cholesterol. All cardiac biomarkers showed statistically significant difference between the groups. All the markers including microRNA-210-3p were higher in group 1 compared to group 2. Similarly, MAGGIC score also showed same type of response among the groups (Table 2).

~~The levels of lipoproteins were higher among group 1 than in group 2. Also, the cardiac biomarkers were significantly higher among group 1 than in group 2.~~ Table 3 shows the levels of biomarkers among the participants according to the gender among the groups.

**Table 3: Shows levels of biomarkers among the participants based on the gender**

| Characteristics | HF Female (n=106) | p value | HF Male (n=164) | p value |
|---|---|---|---|---|

Commented [SS10]: Table 2MAGGIC score data has been added
Formatted: Right: 0.02", Line spacing: single
Formatted: Font: Italic
Formatted: Font: Italic
Formatted: Font: Italic
Formatted: Font: Italic
Formatted: Font: Italic
Formatted: Font: Italic, Font color: Auto
Formatted: Font: Italic
Formatted: Font: Italic
Formatted: Right: 0.02", Line spacing: single
Formatted Table

| | Group 1: EF ≤ 49% (n=34) | Group 2: EF ≥ 50%, (n=72) | | Group 1: EF ≤ 49%, (n=66) | Group 2: EF ≥ 50%, (n=98) | |
|---|---|---|---|---|---|---|
| Age | 61.18 (12.02) | 55.69 (9.86) | 0.01* | 62.09 (11.25) | 53.63 (10.91) | <0.001** |
| NYHA class (n/%) | III (23/22)<br>IV (11/10) | I (68/64)<br>II (4/4) | <0.001** | III (36/22)<br>IV (30/18) | I (92/56)<br>II (6/4) | <0.001** |
| TC | 199.0 (47.52) | 190.10 (36.04) | 0.28~~3~~ | 208.53 (45.87) | 196.39 (39.67) | 0.07~~5~~ |
| TG | 161.74 (63.30) | 129.26 (47.30) | 0.003~~6~~** | 152.74 (63.20) | 164.45 (85.23~~39.67~~) | 0.34~~73~~ |
| HDL | 42.82 (12.85) | 43.93 (10.65) | 0.64~~43~~ | 44.21 (13.15) | 41.90~~81~~ (10.14) | 0.20~~33~~ |
| LDL | 118.62 (41.40) | 122.21 (31.01) | 0.61~~93~~ | 129.83 (38.69) | 125.81 (34.90) | 0.48~~3~~ |
| FPG | 146.50 (63.98) | 121.44 (37.91) | 0.01*~~4~~ | 133.65 (64.89) | 130.95 (49.67) | 0.76~~3~~ |
| PPPG | 223.58 (91.58) | 160.26 (72.38) | <0.001** | 200.57 (92.42) | 169.44 (86.10) | 0.02~~1~~* |
| HbA1c | 8.43 (2.36) | 6.62 (1.38) | <0.001** | 7.83 (2.75) | 6.81 (1.70) | 0.003**~~12~~ |
| BUN | 12.68 (5.88) | 9.90 (3.30) | 0.002~~6~~** | 14.7 (8.21) | 13.58 (1.78) | 0.19~~0.002**~~ |
| Creatinine | 0.86 (0.40) | 0.78 (0.24) | 0.20~~43~~ | 1.04 (0.38) | 0.94 (0.29) | 0.058~~15~~ |
| Hb | 11.62 (1.63) | 11.83 (1.95) | 0.58~~3~~ | 12.72 (2.10) | 13.58 (1.79) | 0.005** |
| NT-proBNP | 482.05 (168.08) | 291.75 (95.11) | <0.001** | 483.09 (66.35) | 284.92 (92.59) | <0.001** |
| sST2 | 38.77 (15.30) | 23.33 (18.19) | <0.001** | 37.97 (12.01) | 19.98 (9.41) | <0.001** |
| Gal-3 | 24.0 (11.22) | 18.91 (8.71) | 0.01*~~7~~ | 25.40 (10.69) | 16.94 (7.86) | <0.001** |
| MiRNA-210-3p# | ~~6 (17)~~ 2.52 (0.78-6.53) | 0.2 (0.09-0.64)~~5 (7)~~ | 0.016*~~9~~ | ~~27 (48)~~1.02 (0.14-6.01) | ~~3 (8)~~0.25 (0.12-0.95) | 0.07~~0.04*~~ |
| MAGGIC Score | 20.88 (5.96) | 6.85 (4.34) | <0.001** | 24.0 (5.65) | 8.93 (4.67) | <0.001** |

*TC: total cholesterol; TG: triglyceride; HDL: high-density lipoprotein, LDL: low-density lipoprotein, NT-proBNP: N-terminal- pro brain natriuretic peptide, sST2: soluble suppression of tumorigenicity, Gal-3: Galectin-3; MiRNA: micro ribonucleic acid; MAGGIC: Meta-Analysis Global Group in Chronic Heart Failure*

*Expressed as mean and SD; Student t test was used; #: expressed as median and interquartile range; Mann-whitney U test was used; **: p value statistically highly significant *: p value statistically significant.*

Among the distribution of age, in both the genders, group 1 HF patients were in older age group compared to group 2. Among the lipids, female patients showed significant alterations in

**Formatted:** Font: (Default) Times New Roman, 12 pt, Not Bold

**Formatted:** Font: (Default) Times New Roman, 12 pt, Not Bold

**Formatted:** Font: (Default) Times New Roman, 12 pt, Not Bold

**Formatted:** Font: (Default) Times New Roman, 12 pt, Not Bold

**Formatted:** Font: (Default) Times New Roman, 12 pt, Not Bold

**Formatted:** Font: (Default) Times New Roman, 12 pt, Not Bold

**Formatted:** Not Highlight

**Formatted:** Not Highlight

**Formatted:** Not Highlight

**Formatted:** Not Highlight

**Formatted:** Not Highlight

**Commented [SS11]:** Table 3 added newly where biomarkers levels are compared according to the gender

triglyceride levels among the groups compared to males. Females showed significant differences in fasting and postprandial glucose levels and HbA1c, while males showed significant differences in postprandial glucose and HbA1c levels. The alterations in the glycemic parameters probably were reflected as altered triglyceride levels in females. Blood urea nitrogen was significantly altered in females compared to males. But creatinine was not significantly altered in both the genders. Cardiac biomarkers such as NT-proBNP, sST2, galectin-3 and MAGGIC score were significantly altered when compared among the groups in both the genders. Even though microRNA-210-3p was elevated in group 1 in both the genders, significance was seen only in females. (Table 3)

The participants were grouped into those with age less than 50 years and those with more than 51 years. Table 4 shows the levels of the biomarkers among the participants based on the age distribution among the groups.

Table 4**: Shows levels of biomarkers among the participants based on the age**

| Characteristics | Age ≤ 50yrs (n=78) | | p value | Age ≥ 51yrs (n=192) | | p value |
|---|---|---|---|---|---|---|
| | Group 1: HF with EF≤49% (n= 17)~~1~~ | Group 2: HF with EF≥ 50% (n= 61)~~1~~ | | Group 1: HF with EF ≤ 49% (n=83)~~1~~ | Group 2: HF with EF ≥ 50% (n = 109)~~1~~ | |
| Gender | | | | | | |
| Male (n/%) | 10 (13) | 42 (55) | 0.31 | 56 (29) | 57 (29) | 0.13 |
| Female (n/%) | 7 (9) | 19 (23) | | 27 (14) | 52 (28) | |
| NYHA class (n/%) | III (13/17)<br>IV (4/5) | I (58/74)<br>II (3/4) | 0.01* | III (46/24)<br>IV (37/19) | I (103/54)<br>II (6/3) | <0.001** |
| TC | 208.33 (39.65) | 201.23 (38.70) | 0.50~~43~~ | 204.55 (47.87) | 189.52 (37.43) | 0.01* |
| TG | 160.59 (59.16) | 163.02 (77.77) | 0.90~~83~~ | 154.82 (64.12) | 142.01 (70.37) | 0.19~~06~~ |
| HDL | 44.94 (11.91) | 43.13 (13.37) | 0.61~~43~~ | 43.49 (13.26) | 42.55 (8.31) | 0.54~~73~~ |
| LDL | 128.88 (30.63) | 130.62 (31.99) | 0.84~~93~~ | 125.43 (41.54) | 120.73 (33.57) | 0.38~~3~~ |
| FPG | 141.86 (81.47) | 128.0 (48.32) | 0.37~~93~~ | 136.95 (58.75) | 126.62 (43.87) | 0.16~~33~~ |
| PPPG | 269.88 (30.63) | 163.50 (78.22) | ~~0.07~~<0.001 | 200.53 (79.50) | 166.10 (81.22) | ~~<~~0.003~~1~~** |
| HbA1c | 7.49 (2.98) | 6.62 (1.68) | 0.12~~53~~ | 8.14 (2.55) | 6.79 (1.51) | <0.001~~3~~** |
| BUN | 12.06 (6.59) | 9.9 (3.1) | 0.057~~53~~ | 14.41 (7.68) | 10.72 (3.69) | <0.001** |

Formatted: Font: 11 pt
Formatted: Font: 11 pt
Formatted Table
Formatted: Font: 11 pt
Formatted: Font: 11 pt
Formatted: Font: 11 pt
Formatted: Font: 11 pt
Formatted: Font: 11 pt
Formatted: Font: 11 pt
Formatted: Font: 11 pt
Formatted: Font: 11 pt
Formatted: Centered
Formatted: Centered
Formatted: Font: 11 pt
Formatted: Font: 11 pt
Formatted: Centered
Formatted: Centered
Formatted: Font: 11 pt
Formatted: Font: 11 pt
Formatted: Font: 11 pt
Formatted: Font: 11 pt
Formatted: Font: 11 pt
Formatted: Font: 11 pt
Formatted: Font: 11 pt
Formatted: Font: 11 pt

| | | | | | | |
|---|---|---|---|---|---|---|
| Creatinine | 0.88 (0.40) | 0.86 (0.25) | 0.80~~63~~ | 1.00 (0.40) | 0.88 (0.30) | 0.01* |
| Hb | 12.70 (2.10) | 13.19 (2.02) | 0.38~~43~~ | 12.26 (2.00) | 12.65 (2.04) | 0.18~~08~~ |
| NT-proBNP | 426.66 (50.15) | 266.33 (94.24) | <0.001** | 494.22 (116.65) | 299.83 (91.24) | <0.00~~1~~** |
| sST2 | 43.02 (17.12) | 20.97 (10.09) | <0.001** | 37.26 (12.07) | 21.64 (15.64) | <0.001** |
| Gal-3 | 27.39 (10.53) | 17.62 (8.86) | <0.001** | 24.42 (10.90) | 17.86 (7.99) | <0.001** |
| MiRNA-210-3p# | ~~24 (47)~~0.53 (0.05-3.01) | ~~3 (6)~~0.23 (0.13-2.38) | 0.59~~0.43~~ | ~~13 (31)~~1.89 (0.16-14.26) | 0.25 (0.10-0.87)~~4 (12)~~ | 0.001**~~0.53~~ |
| MAGGIC score | 16.65 (4.61) | 5.52 (2.16) | <0.001** | 24.23 (5.32) | 9.46 (5.03) | <0.001** |

*TC: total cholesterol; TG: triglyceride; HDL: high-density lipoprotein, LDL: low-density lipoprotein, NT-proBNP: N-terminal- pro brain natriuretic peptide, sST2: soluble suppression of tumorigenicity, Gal-3: Galectin-3; MiRNA: micro ribonucleic acid; MAGGIC: Meta-Analysis Global Group in Chronic Heart Failure*

*Expressed as mean and SD; Student t test was used; #: expressed as median and interquartile range; Mann-whitney U test was used; **: p value statistically highly significant *: p value statistically significant.*

There was no significant difference when compared between the genders with respective to age. NYHA class showed significant difference among the groups in both the age groups. Among the lipids, only total cholesterol showed significant difference among the groups when age of the participants were more than 51 years. Among glycemic parameters, post-prandial glucose and HbA1c levels are significantly increased in individuals more than 51 years of age. Among renal parameters, both urea and creatinine showed significantly higher levels in the individuals of more than 51 years of age. This is probably due to the fact that long-standing metabolic disorders cause end organ damage. NT-proBNP, sST2, galectin-3and MAGGIC score were significantly increased in group 1 irrespective of age of the participants. But, MicroRNA-210-3p showed significantly high values of group 1 individuals of more than 51 years of age (Table 4).

Figure 2 shows the microRNA-210-3p amplification patterns in groups I and 2 of heart failure patients.

**Figure 2: MicroRNA amplification patterns among group 1 and group 2 HF patients**

Formatted: Font: 11 pt
Formatted: Font: 11 pt
Formatted: Font: 11 pt
Formatted Table
Formatted: Font: 11 pt
Formatted: Font: 11 pt
Formatted: Font: 11 pt
Formatted: Font: 11 pt, Not Highlight
Formatted: Font: 11 pt, Not Highlight
Formatted: Font: 11 pt, Not Highlight
Formatted: Font: 11 pt
Formatted: Font: 11 pt, Not Highlight
Formatted: Font: 11 pt, Not Highlight
Formatted: Font: 11 pt, Not Highlight
Formatted: Font: 11 pt
Formatted: Font: 11 pt
Formatted: Font: 11 pt
Formatted: Font: 11 pt
Formatted: Font: 11 pt
Formatted: Font: 11 pt

The analysis of biomarkers for heart failure prediction revealed that the MAGGIC score demonstrated the best overall performance with the highest AUC of 0.86 (95% CI: 0.86-0.90) (p=0.001), cut-off score of 14 with sensitivity of 95% and specificity of 89%, suggesting its reliability for risk stratification. Among the individual biomarkers, miRNA-210-3p showed the most promise as a novel biomarker, outperforming traditional markers with a higher AUC of 0.79 (95% CI: 0.68-0.89), sensitivity of 87%, and specificity of 54% at a cut-off level of 5.03. Traditional biomarkers showed moderate predictive ability: NT-proBNP had an AUC of 0.65 (95% CI: 0.58-0.71) with 64% sensitivity and 46% specificity at a cut-off of 355.5, while sST2 had an AUC of 0.58 (95% CI: 0.52-0.65) with 60% sensitivity and 46% specificity at a cut-off of 20.11. Galectin-3 appeared to have limited value, with an AUC of 0.51 (95% CI: 0.44-0.58) and a non-significant p value of 0.60 (Table 5, Fig 3)

Commented [SS12]: Modified table 5 and fig 1 with inclusion of MAGGIC score

**Table 5: Cut-off level of the biomarkers in patients with HF**

| **Variable** | **AUC** | **SE** | **p value** | **95%CI** | **Cut-off level** | **Sensitivity (%)** | **Specificity (%)** |
|---|---|---|---|---|---|---|---|
| NT-proBNP | 0.65 | 0.03 | 0.001** | 0.58-0.71 | 355.5 | 64 | 46 |
| sST2 | 0.58 | 0.03 | 0.01* | 0.52-0.65 | 20.11 | 60 | 46 |
| Gal-3 | 0.51 | 0.03 | 0.60 | 0.44-0.58 | 17.58 | 55 | 57 |
| MiRNA-210-3p | 0.79 | 0.05 | <0.~~0~~001** | 0.68-0.89 | 5.03 | 87 | 54 |
| MAGGIC score | 0.86 | 0.02 | 0.001 | 0.86-0.90 | 14 | 95 | 89 |

*NT-proBNP: N-terminal- pro brain natriuretic peptide, sST2: soluble suppression of tumorigenicity, Gal-3: galectin-3; MiRNA: micro ribonucleic acid; MAGGIC: Meta-Analysis Global Group in Chronic Heart Failure*
***: p value statistically highly significant *: p value statistically significant.*

**Figure 3: ROC curve showing the cut-off level of the biomarkers in patients with HF**

Formatted: Font: Italic
Formatted: Font: Italic
Formatted: Font: Italic
Formatted: Font: Italic
Formatted: Font: Italic
Formatted: Line spacing: single

Table 6 shows the correlation of NT-proBNP with MAGGIC score, other cardiac markers, microRNA-210-3p, lipid profile and HbA1c

## Table 6: Correlation between NT-proBNP and other novel biomarkers in patients with HF

| ~~Variable~~ | ~~Correlation with variable~~ | ~~r value~~ | ~~p value~~ |
|---|---|---|---|
| ~~NT-proBNP~~ | ~~sST2~~ | ~~0.41~~ | ~~0.001**~~ |
| - | ~~Galectin-3~~ | ~~0.23~~ | ~~0.001**~~ |
| ~~sST2~~ | ~~Galectin-3~~ | ~~0.13~~ | ~~0.02*~~ |
| ~~microRNA-210-3p~~ | ~~NT-proBNP~~ | ~~0.13~~ | ~~0.23~~ |
| - | ~~sST2~~ | ~~0.03~~ | ~~0.77~~ |
| - | ~~Galectin-3~~ | ~~0.24~~ | ~~0.03*~~ |

| Correlation Matrix | MAGGIC score | NT-proBNP | sST2 | Gal-3 | MiR-210-3p | TC | TG | HDL | LDL | HbA1c |
|---|---|---|---|---|---|---|---|---|---|---|
| MAGGIC score | - | | | | | | | | | |
| NT-proBNP | 0.676<br><0.001 | - | | | | | | | | |
| sST2 | 0.405<br><0.001 | 0.416<br><0.001 | - | | | | | | | |
| Gal-3 | 0.281<br><0.001 | 0.231<br><0.001 | 0.136<br>0.025 | - | | | | | | |
| MiR-210-3p | 0.136<br>0.167 | 0.067<br>0.497 | 0.087<br>0.377 | 0.160<br>0.102 | - | | | - | | |
| TC | 0.063<br>0.301 | 0.118<br>0.053 | 0.121<br>0.047 | 0.011<br>0.858 | 0.03<br>0.761 | - | | | | |
| TG | 0.016<br>0.799 | 0.003<br>0.963 | 0.012<br>0.848 | 0.078<br>0.203 | 0.173<br>0.077 | 0.337<br><0 .001 | - | | | |
| HDL | -0.081<br>0.183 | -0.04<br>0.515 | 0.098<br>0.108 | -0.061<br>0.318 | 0.015<br>0.876 | -0.501<br><0.001 | -0.061<br>0.32 | - | | |
| LDL | 0.058<br>0.341 | 0.041<br>0.507 | 0.034<br>0.578 | 0.003<br>0.960 | 0.010<br>0.921 | 0.820<br><0 .001 | 0.339<br><0.001 | -0.321<br><0.001 | - | |
| HbA1c | 0.286<br><0.001 | 0.268<br><0.001 | 0.159<br>0.012 | 0.045<br>0.484 | 0.152<br>0.124 | 0.107<br>0.093 | 0.024<br>0.702 | -0.065<br>0.306 | 0.076<br>0.232 | - |

*NT-proBNP: N-terminal- pro brain natriuretic peptide, ST2: suppression of tumorigenicity, RNA: ribonucleic acid*

***: P value statistically highly significant *: P value statistically significant.*

**Formatted:** Font: Not Bold

**Commented [SS13]:** Table 6 shows the correlation among cardiac markers, lipids, MAGGIC, microRNA and HbA1c

**Formatted Table**

**Formatted:** Centered

**Formatted:** Font: 10 pt

Widely accepted MAGGIC score showed positive correlation with NT-proBNP, sST2 and galectin-3. There was positive correlation among NT-proBNP, sST2 and galectin-3. But microRNA did not show any correlation with any of the variables probably due to small sample size. NT-proBNP and MAGGIC score showed positive correlation with HbA1c. sST2 showed positive correlation with total cholesterol and HbA1c. Total cholesterol showed positive correlation with triglyceride and LDL cholesterol and negative correlation with HDL cholesterol (Table 6).

~~Table 3 and figure 1 show the ROC curves and the cut-off levels of the biomarkers~~

**~~Table 3: Cut-off level of the biomarkers in patients with HF~~**

| ~~Variable~~ | ~~AUC~~ | ~~SE~~ | ~~p value~~ | ~~95%CI~~ | ~~Cut-off level~~ | ~~Sensitivity (%)~~ | ~~Specificity (%)~~ |
|---|---|---|---|---|---|---|---|
| ~~NT-proBNP~~ | ~~0.65~~ | ~~0.03~~ | ~~0.001**~~ | ~~0.58-0.71~~ | ~~355.5~~ | ~~64~~ | ~~46~~ |
| ~~sST2~~ | ~~0.58~~ | ~~0.03~~ | ~~0.01*~~ | ~~0.52-0.65~~ | ~~20.11~~ | ~~60~~ | ~~46~~ |
| ~~Galectin-3~~ | ~~0.51~~ | ~~0.03~~ | ~~0.60~~ | ~~0.44-0.58~~ | ~~17.58~~ | ~~55~~ | ~~57~~ |
| ~~miRNA-210-3p~~ | ~~0.79~~ | ~~0.05~~ | ~~0.0001**~~ | ~~0.68-0.89~~ | ~~5.03~~ | ~~87~~ | ~~54~~ |

~~NT-proBNP: N terminal- pro brain natriuretic peptide, ST2: suppression of tumorigenicity, RNA: ribonucleic acid~~

~~**: p value statistically highly significant *: p value statistically significant.~~

**~~Figure 1: ROC curve showing the cut-off level of the biomarkers in patients with HF~~**

**Formatted:** Strikethrough

**Formatted:** Strikethrough

**Formatted:** Strikethrough

**Formatted:** Strikethrough

**Formatted:** Strikethrough

**Formatted:** Strikethrough

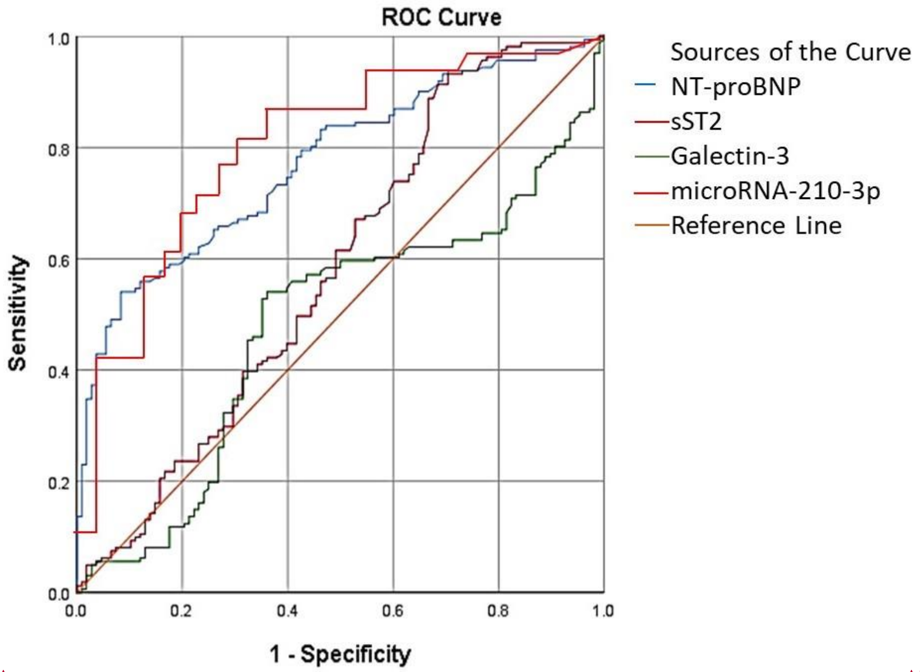


**Table 4: Correlation between NT-proBNP and novel biomarkers in patients with HF**

| Variable | Correlation with variable | r value | p value |
|---|---|---|---|
| NT-proBNP | sST2 | 0.41 | 0.001** |
| - | Galectin-3 | 0.23 | 0.001** |
| sST2 | Galectin-3 | 0.13 | 0.02* |
| microRNA-210-3p | NT-proBNP | 0.13 | 0.23 |
| - | sST2 | 0.03 | 0.77 |
| - | Galectin-3 | 0.24 | 0.03* |

NT-proBNP: N-terminal- pro-brain natriuretic peptide, ST2: suppression of tumorigenicity, RNA: ribonucleic acid

**: P value statistically highly significant *: P value statistically significant.

NT-proBNP showed positive correlations with sST2 and galectin-3. microRNA-210-3p

Formatted: Strikethrough

Formatted: Strikethrough

Formatted: Left, Space Before: Auto, After: Auto, Line spacing: single

Formatted: Strikethrough

Formatted: Strikethrough

Formatted: Strikethrough

Formatted: Strikethrough

Formatted: Strikethrough

Formatted: Strikethrough

Formatted: Strikethrough

showed positive correlation with galectin-3 alone as shown in table 4.

**Figure 2: MicroRNA amplification patterns among group 1 and group 2 HF patients**

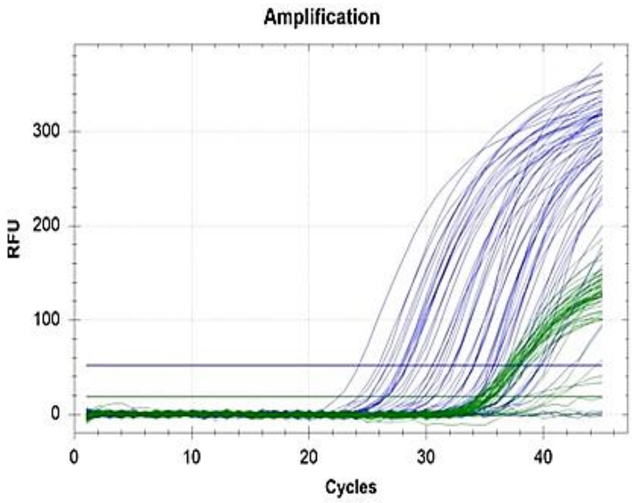


**Group 1**

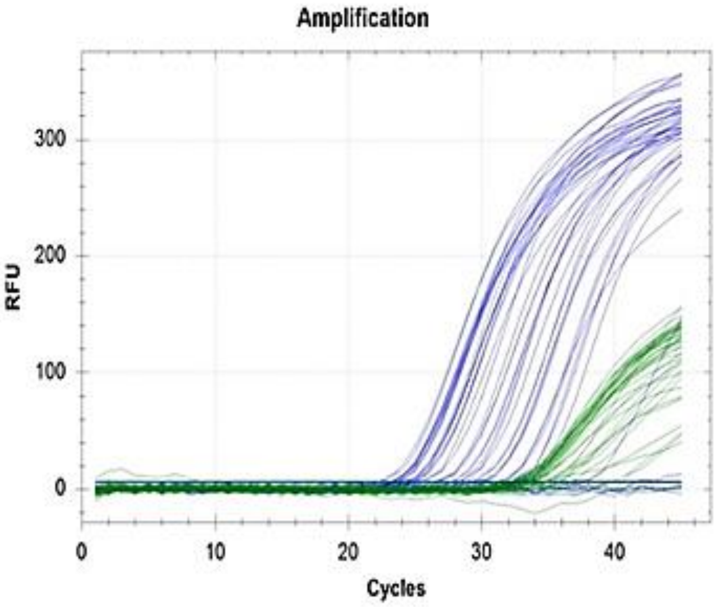

~~**Group 2**~~

# Discussion

Heart failure (HF) is the consequence of various cardiac and noncardiac conditions [1]. In humans, after the sixth decade of life, the incidence of HF doubles in men whereas it triples in women [27~~6~~]. The Cardiology Society of India-Kerala Acute Heart Failure Registry (CSI-KHFR) indicates that India is facing a huge threat of HF due to large population, and increased prevalence of diabetes mellitus, obesity, hypertension, pulmonary diseases and sedentary life style [28~~7~~]. In India, the states such as Kerala, Punjab and Tamil Nadu are the most affected, probably due to increased prevalence of dyslipidemia and hypertension [29~~8~~]. The pathological heart remodeling also varies according to the etiology of HF. AMI stimulates sterile inflammation, which in turn leads to replacement fibrosis and eccentric hypertrophy in HFrEF. HFpEF due to hypertension have concentric hypertrophy and interstitial fibrosis in the heart. Therapeutic options that are available for HFrEF are shown to be not very beneficial in HFpEF. Diagnostic procedures which may be able to distinguish the underlying pathological processes, may be useful in the less understood HF phenotypes [7].

In the present study, the presenting clinical features in group 1 were dyspnea, pedal edema and palpitation while group 2 patients complained mainly of fatigue. In group 1 (HF≤49%), 62% patients were in the advancing age of 61 to 75 years, while in group 2 (HF≥50%), 70% were 46 to 60 years of age. The younger age involvement in group 2 indicated the earlier onset of symptoms due to pathogenesis set by the underlying disorder such as hypertension and type 2 diabetes mellitus (T2DM). ~~Although group 1 patients also had hypertension with or without diabetes mellitus, the predisposing condition in these individuals was AMI.~~ Males were predominantly affected irrespective of EF. Males tend to be obese, with earlier onset of hypertension and T2DM. ~~The distribution of patients according to BMI were almost the same among the two groups.~~ Most of the participants were in the overweight category in both the

groups. This clearly indicated that obesity is an impending risk for development of hypertension and T2DM with consequent HF (Table 1). ~~Among the participants, 33% were smokers, 35% were alcoholics, and 46% were vegetarians.~~ ~~All the patients had normal renal functions and hemoglobin concentrations.~~ According to Teramoto et al, the mean age of HF patients in India is 58 to 68 years, which is about a decade younger than that of Western HF patients. The prevalence of HFpEF is higher than that of HFrEF. HFpEF is higher in women, while HFrEF is higher in men. HFpEF phenotype tends to increase with ageing of the population, especially among patients of more than 64 years of age [30~~29~~].

In the present study, ~~the participants in both the groups had higher triglycerides and low-density lipoprotein cholesterol (LDL) and lower high-density lipoprotein cholesterol (HDL), however they were not statistically significant between the groups.~~ ~~G~~group 1 patients had higher total cholesterol than that of group 2 (p=0.01) (Table 2). Since the patients were hypertensives while ~~50%~~ 60^ of them had ~~diabetes~~ T2DM also, atherosclerotic changes in the blood vessels could be present as evidenced by ~~increased LDL~~dyslipidemia ~~and decreased HDL~~. ~~Lipid profile did not show significant correlation with biomarkers such as NT-proBNP, sST2 and galectin-3.~~ Studies done by Jarab et al, have shown that elevated LDL and decreased HDL are associated with reduced cardiac function. Inflammation associated with dyslipidemia plays a major role in the exacerbation of HF [31~~0~~]. Yao et al, have concluded that dyslipidemia induced by oxidative stress, inflammation, altered autophagy, microvascular density, and cardiomyocyte mitochondrial dysfunction lead to impaired cardiac structure and function. The measures adopted to decrease serum lipids are found to improve the ventricular functioning capacity~~.~~ [32~~1~~].

The MAGGIC score is used to predict mortality as well as stratify the patients with HF. It can be done with the routinely available parameters [22]. However, MAGGIC score is not able to predict the frequency of hospitalization, morbidity and it does not include brain natriuretic

peptide levels. Hence, relying on biomarkers is a better choice for diagnosis as well as prognosis of HF. According to Piek et al, ventricular myocyte stress stimulates the synthesis and release of natriuretic peptides (NPs) [33~~2~~]. BNP or NT-proBNP is considered to be the gold standard marker for the diagnosis and prognosis of HF [34~~3~~]. Also, NPs can identify asymptomatic HF in individuals with poor LV function. BNP acts by regulating the renin–angiotensin~~–~~aldosterone system (RAAS) and the sympathetic nervous system [35~~4~~]. Thus, it causes vasodilatation, increases glomerular filtration rate (GFR) and prevents salt reabsorption, thus, promoting ~~. This results in reduction in blood pressure, ventricular preload, promotion of~~ natriuresis and diuresis [36~~5~~]. ~~In acute HF, NPs may be normal in levels, since there is not enough time for the synthesis of NPs to get increased. Celik et al, has stated that stage 1 hypertensive patients with elevated NT-proBNP have increased CVD risk than that of stage 2 hypertensives with normal NT-proBNP levels [34].~~ In the present study, NT-proBNP levels were 482.74 ± 110.94 pg/mL and 287.81 ± 93.45pg/mL in groups 1 and 2 respectively (p=0.001) (Table 2). This indicated that group 1 patients had more severe LV volume overload than that of group 2 patients. According to the study by Kang et al, ~~the mortality and morbidity are high in patients with diastolic dysfunctions with increased levels of NT-proBNP. A~~although patients with HFpEF have lower NT-proBNP levels, the prognosis is similar with that of HFrEF [37~~6~~]. ~~Reductions in the plasma BNP concentration predict improvements in the clinical symptoms [34].~~ Patients who exhibit less than 30% reduction in BNP levels show increased risk of 180-day mortality [38~~7~~]. ~~As per the ESC guidelines 2016, acute HF patients are recommended to include plasma NPs in assessment of LV function.~~ In non-acute conditions, especially when ~~echocardiography (~~ ECHO~~)~~ is not immediately available, elevated NPs may help to establish an initial diagnosis~~, and to identify patients requiring further investigations or advanced treatment~~ [3~~8]~~9. According to Cao et al, BNP levels help clinicians to distinguish whether the dyspnea is of cardiac or noncardiac origin ~~. Elevated NT-proBNP~~

Commented [SS14]: Modified

levels can be used to confirm the diagnosis of HF, however, low levels may not be able rule out HF, especially HFpEF [4039].

Receiver operating characteristics (ROC) curve showed an area under the curve (AUC) for NT-proBNP of 0.65 with 95% confidence interval (CI) of 0.58 to 0.71 (p=0.001). The cut-off value of NT-proBNP was 355.5pg/mL with sensitivity and specificity of 64% and 46% respectively (Table 3, Fig 1). In group 1, 96% and 4% of the individuals had NT-proBNP values higher and lower than the cut-off value respectively. In group 2, 48% and 52% of the individuals had NT-proBNP values higher and lower than the cut-off value respectively. Since 96% of the individuals in group 1 had higher NT-proBNP levels than the cut-off, it proves that it is a sensitive marker in diagnosing HFrEF. Since only 48% of the individuals in group 2 had higher values than the cut-off value, NT-proBNP alone may not be able to diagnose HF in patients having EF more than 50%. A normal electrocardiogram and/or plasma concentrations of BNP <35 pg/mL and/or NT-proBNP <125 pg/mL may be used in diagnosis of HF [39]. In young healthy adults, BNP and NT-proBNP levels are <25pg/mL and <70pg/mL respectively [40]. When BNP is <100pg/mL, HF may not be the cause for breathlessness and that alternative etiologies for dyspnea should be investigated. If BNP level is 100 to 500pg/mL, clinical findings are needed to support the diagnosis of HF. When BNP is > 500pg/mL, HF is definitely present and that quick management for HF is needed [39].NT-proBNP has certain limitations, which precludes NPs from being used as a gold standard marker. According to Cao et al, different cut-off values used, are based on whether the HF is of acute or chronic [4039]. Yancy et al, has concluded that there is no consensus in arriving at the age-adjusted NP cut-off levels [4039]. Old age and smoking are associated with elevated NT-proBNP in males. Diabetes mellitus is inversely associated with elevated NT-proBNP levels in females [41]. In obesity, there is decreased production and increased clearance of NPs which results in disproportionately low NP levels in obese HF. Hence, lower cut-offs are needed for diagnosing HF in patients with obesity [42]. BNP levels rise with fall in GFR. When GFR is <60mL/min/1·73 $m^2$, LV hypertrophy (LVH) develops

along with systolic and diastolic LV dysfunctions. The PRIDE study showed that a higher cut-off of NT-proBNP of >1,200pg/mL may be used for diagnosing HF in chronic kidney disease (CKD) [43].

The 2013 ACC Foundation/ AHA guidelines have recommended that in addition to NPs, biomarkers of inflammation and fibrosis may be used to refine prognostic stratification of HF [44]. The 2016 ESC Guidelines stated that soluble Suppression of Tumorigenicity 2 (sST2) and other fibrosis biomarkers can be used in clinical practice of HF [45]. sST2 is a member of the Toll-like receptor binding interleukin (IL)-33 [11]. Alternative splicing and processing of the mRNA of ST2 gene leads to the production of either sST2 or ST2L [46]. When the IL-33/ST2L signalling mechanism is activated, it results in anti-apoptotic, anti-fibrotic and anti-hypertrophic effects [11]. sST2 production is induced by circulatory overload causing mechanical stretch, inflammation, and fibrosis [46]. sST2 acts as a decoy receptor for IL-33 and blocks the action of IL-33, thus resulting in myocardial hypertrophy [11]. Generally, sST2 levels are low, enabling IL-33 to maintain cardioprotective effects. In HF, the elevated sST2 levels prevent ST2L from binding to IL-33. IL-33/sST2 complex thus formed, does not have a protective role on the heart. sST2 is considered as a novel biomarker of cardiac strain [47]. In the present study, sST2 levels were 38.23±13.14ng/mL and 21.40±13.87 in groups 1 and 2 respectively ($p=0.001$) (Table 2). This probably indicated that sST2 indicated myocardial fibrosis, which could be higher in group 1 than that in group 2.

According to Aimo et al., acute HF patients with elevated sST2 levels at the time of discharge, are considered to be at high risk for prolonged hospital stay, increased dosage of HF drugs, and frequent hospital visits. In HFpEF, sST2 concentrations are associated with systemic inflammation, right ventricle (RV) dysfunction, and systemic congestion [46]. Increased plasma sST2 concentrations are associated with all-cause and cardiovascular mortality [48]. sST2 could also be a promising tool to identify the risk of sudden cardiac death (SCD) in

patients with low EF. The lower biological variability of sST2 in comparison to NT-proBNP, makes sST2 a promising marker of assessing prognosis of HF patients [49]. Soluble ST2 levels rise several weeks before a cardiac event; thus, serving as a sensitive marker in diagnosis of HF [13]. sST2 predicts end-point events better than NT-proBNP, since sST2 is not affected by BMI, renal function, age, etiology or other traditional risk factors of HF [49]. The percentage change in sST2 levels on repeated measurements during hospitalization is predictive of three-month death regardless of NP levels [11]. Elevated sST2 levels predict ventricular arrhythmia, LV diastolic dysfunction, increased RV systolic pressure, and hypokinesis in chronic HF [46]. Asymptomatic individuals have higher levels of sST2 than that of healthy individuals [49]. ST2 levels in combination with NT-proBNP could be better in risk stratification [50]. By measuring sST2 levels, patients at a risk of adverse LV remodeling can be identified [51].

According to the 2017 ACC/AHA/HFSA Guidelines, galectin-3 (Gal-3) is suggested to be a diagnostic marker for risk stratification and prognosis evaluation in HF patients [52]. A study done by Gehlken et al, has concluded that when Gal-3 is used as a sole marker, it could predict 60-day prognosis in acute HFpEF. In hypertensive individuals, Gal-3 can detect early onset HFpEF. Its levels are directly related to the severity of diastolic dysfunction [53]. Although sST2 and Gal-3 have similar prognostic performances in both HFrEF and HFpEF, sST2 is considered to be superior to Gal-3. Gal-3 is a member of the β-galactoside-binding lectin family and is produced by macrophages [11]. It is highly stable and resistant to hemodynamic alterations, and is an early indicator of cardiac fibrosis, ventricular remodeling, and renal impairment in HF patients; however, it has low specificity [54]. Under hypoxic or hypocaloric conditions, Gal-3 expression is upregulated, through the activity of hypoxia inducible factor- 1 alpha (HIF-1α) and nuclear factor – kappa beta (NF-κB); further formed Gal-3 enters systemic circulation. Gal-3 activates mitogen activated protein kinase (MAPK) signal transduction pathway, leading to cell proliferation and cell survival [6]. In the present study, serum Gal-3

levels were 24.93±10.84 ng/mL and 17.77±8.29 in groups 1 and 2 respectively (p=0.001) (Table 2). According to Gehlken et al, Gal-3 plays an important role in inflammation, fibrosis and ventricular remodeling [53]. Gal-3 is an independent predictor of mortality in patients with moderate to advanced chronic HF [55].

Studies are being done to assess the diagnostic and prognostic potential of epigenetic markers such as micro-RNAs (miRNA), long non-coding RNAs, and exosome cargo, because of their potential role in the early phases of cardiac dysfunction. miRNAs play critical roles in heart development, function, and response to ischemia/reperfusion injury. There is increasing evidence, that miRNAs may have a key role in the pathogenesis of HF through regulation of genes involved in the adverse ventricular remodelling [56]. The dysregulation of miRNAs, and their target genes, are related to signaling pathways of HF, including cardiac hypertrophy, oxidative stress, cardiac fibrosis, etc [57]. The circulating microRNAs are relatively stable, sensitive, specific and easily detectable by non-invasive methodology, hence, circulating microRNAs may be ideal candidate biomarkers and drug targets for HF [57]. In response to hypoxia, specific set of microRNAs, called hypoxamirs are upregulated [56]. In the present study, microRNA 210-3p showed increased expression of 11.89-fold in group 1and 1.08-fold in group 2 compared to controls. There was statistically significant difference with p value of 0.031 (Table 2, Fig 2). According to Shen et al, the most down-regulated miRNA is miR-30c, and the most upregulated is miR-210-3p [57]. Through the HIF-1α pathway, miR-210-3p is independently associated with HF, cardiovascular mortality and cardiac regeneration. miR-210-3p is found to inhibit apoptosis of cardiomyocytes. HF patients having increased expression levels of miR-210-3p show frequent HF decompensations and adverse ventricular remodelling [56]. MiRNAs can be used alone or in combination with other biomarkers of HF such as BNP [58].

Table 3 shows the distribution of study characteristics according to the gender of the

participants. In females, the alterations in the glycemic parameters probably were in parallel with that of altered triglyceride levels. Altered blood urea with normal creatinine levels indicated that females are predisposed to prerenal uraemia earlier compared to males, probably due to anatomical architecture of females. Group 1 patients had higher levels of cardiac markers and MAGGIC score when compared to group 2 patients. Even though microRNA-210-3p was elevated in group 1 in both the genders, significance was seen only in females. MicroRNA could serve as a better diagnostic and prognostic markers, since females tend to have poor prognosis compared to males (Table 3) Table 4 shows the distribution of study parameters according to the age of above or below the age of 50 years. Among the lipids, only total cholesterol showed significant difference among the groups when age of the participants were more than 51 years. Among glycemic parameters, post-prandial glucose and HbA1c levels are significantly increased in individuals more than 51 years of age. Among renal parameters, both urea and creatinine showed significantly higher levels in the individuals of more than 51 years of age. All these changes indicated that the long-standing metabolic disorders cause end organ damage (Table 4).

Receiver operating characteristics (ROC) curve showed an area under the curve (AUC) for NT-proBNP of 0.65 (p=0.001). The cut-off value of NT-proBNP was 355.5pg/mL with sensitivity and specificity of 64% and 46% respectively (Table 5, Fig 3). A normal electrocardiogram and/or plasma concentrations of BNP <35 pg/mL and/or NT-proBNP <125 pg/mL may be used in diagnosis of HF [40]. In young healthy adults, BNP and NT-proBNP levels are <25pg/mL and <70pg/mL respectively [59]. When BNP is <100pg/mL, HF may not be the cause for breathlessness and that alternative etiologies for dyspnea should be investigated. If BNP level is 100 - 500pg/mL, clinical findings are needed to support the diagnosis of HF. When BNP is > 500pg/mL, HF is definitely present and that quick management for HF is needed [40]. The AUC for sST2 of 0.58 with

**Formatted:** Body Text, Right: 0.02"

NT-proBNP has certain limitations, which precludes NPs from being used as a gold standard marker. According to Cao et al, different cut-off values used, are based on whether the HF is of acute or chronic [39]. Yancy et al, has concluded that there is no consensus in arriving at the age-adjusted NP cut-off levels [39]. Old age and smoking are associated with elevated NT-proBNP in males. Diabetes is inversely associated with elevated NT-proBNP in females [41]. In obesity, there is decreased production and increased clearance of NPs which results in disproportionately low NP levels in obese HF. Hence, lower cut-offs are needed for diagnosing HF in patients with obesity [42]. BNP levels rise with fall in GFR. When GFR is <60mL/min/1·73 $m^2$, LV hypertrophy (LVH) develops along with systolic and diastolic LV dysfunctions. The PRIDE study showed that a higher cut-off of NT-proBNP of >1,200pg/mL may be used for diagnosing HF in chronic kidney disease (CKD) [43].

The 2013 ACC Foundation/ AHA guidelines have recommended that in addition to NPs, biomarkers of inflammation and fibrosis may be used to refine prognostic stratification of HF [44]. The 2016 ESC Guidelines stated that soluble Suppression of Tumorigenicity 2 (sST2) and other fibrosis biomarkers can be used in clinical practice of HF [45]. sST2 is a member of the Toll-like receptor binding interleukin (IL)-33 [11]. Alternative splicing and processing of the mRNA of ST2 gene leads to the production of either sST2 or ST2L [46]. When the IL-33/ST2L signalling mechanism is activated, it results in anti-apoptotic, anti-fibrotic and anti-hypertrophic effects [11]. sST2 production is induced by circulatory overload causing mechanical stretch, inflammation, and fibrosis [46]. sST2 acts as a decoy receptor for IL-33 and blocks the action of IL-33, thus resulting in myocardial hypertrophy [11]. Generally, sST2 levels are low, enabling IL-33 to maintain cardioprotective effects. In HF, the elevated sST2 levels prevent ST2L from binding to IL-33. IL-33/sST2 complex thus formed, does not have a protective role on the heart. sST2 is considered as a novel biomarker of cardiac strain [47]. In

**Formatted:** Right: 0"

**Formatted:** Body Text

the present study, sST2 levels were 38.23±13.14ng/mL and 21.40±13.87 in groups 1 and 2 respectively (p=0.001) (Table 2). This probably indicated that sST2 indicated myocardial fibrosis, which could be higher in group 1 than that in group 2. According to Aimo et al., acute HF patients with elevated sST2 levels at the time of discharge, are considered to be at high risk for prolonged hospital stay, increased dosage of HF drugs, and frequent hospital visits. In HFpEF, sST2 concentrations are associated with systemic inflammation, right ventricle (RV) dysfunction, and systemic congestion [46]. Increased plasma sST2 concentrations are associated with all-cause and cardiovascular mortality [48]. sST2 could also be a promising tool to identify the risk of sudden cardiac death (SCD) in patients with low EF. The lower biological variability of sST2 in comparison to NT-proBNP, makes sST2 a promising marker of assessing prognosis of HF patients [49]. Soluble ST2 levels rise several weeks before a cardiac event; thus, serving as a sensitive marker in diagnosis of HF [13]. sST2 predicts end-point events better than NT-proBNP, since sST2 is not affected by BMI, renal function, age, etiology or other traditional risk factors of HF [49]. The percentage change in sST2 levels on repeated measurements during hospitalization is predictive of three-month death regardless of NP levels [11]. Elevated sST2 levels predict ventricular arrhythmia, LV diastolic dysfunction, increased RV systolic pressure, and hypokinesis in chronic HF [46]. Asymptomatic individuals have higher levels of sST2 than that of healthy individuals [49]. ST2 levels in combination with NT-proBNP could be better in risk stratification [50]. By measuring sST2 levels, patients at a risk of adverse LV remodeling can be identified [51].

In the present study, ROC curve showed AUC for sST2 of 0.58 with 95% CI of 0.52 to 0.65 (p=0.01). The cut-off value of sST2 was 20.1ng/mL with sensitivity and specificity of 60% and 46% respectively. (Table 35, Fig 31) In group 1, 94% and 6% of the individuals had sST2 values higher and lower than the cut-off value respectively. In group 2, 33% and 67% of the individuals had sST2 values higher and lower than the cut-off value respectively. Around 95%

~~of group 1 patients had sST2 and NT-proBNP above the cutoff levels. But, in group 2 only 33% of patients had high sST2 levels compared to NT-proBNP which was elevated in 48% of the individuals. However, ~~ s~~S~~ince sST2 shows less biological intraindividual variability on repeated measurements, sST2 could be a better biomarker of HF than NT-proBNP. The study by Castiglione et al, shows a higher cutoff of 35 ng/mL for risk stratification in acute HF [11]. This is much higher than the cut-off of the present study probably due to variations in the eth~~i~~nicity and the race of the study population. ~~According to Aleksova et al, sST2 levels of 35 -70ng/mL are associated with mild and moderate acute HF and levels >70ng/mL are associated with severe HF, these patients have significant activation of the neurohormonal system and fibrotic pathways. It is mandatory for immediate hospitalization and treatment with the aim of bringing down the sST2 levels by at least 30% at the time of discharge [49]. ~~sST2 and NT-proBNP levels are significantly higher in HFrEF than in patients with HFmrEF and HFpEF. The present study showed almost the same cut-off as that of the PRIDE study. In the PRIDE study, sST2 concentration of >17.26 ng/mL have a stronger correlation with adverse prognosis in acute HF and patients with increased sST2 levels show high risk of death at one year [43].

~~According to the 2017 ACC/AHA/HFSA Guidelines, galectin 3 (Gal-3) is suggested to be a diagnostic marker for risk stratification and prognosis evaluation in HF patients [52]. A study done by Gehlken et al, has concluded that when Gal-3 is used as a sole marker, it could predict 60-day prognosis in acute HFpEF. In hypertensive individuals, Gal-3 can detect early onset HFpEF. Its levels are directly related to the severity of diastolic dysfunction [53]. Although sST2 and Gal-3 have similar prognostic performances in both HFrEF and HFpEF, sST2 is considered to be superior to Gal-3. Gal-3 is a member of the β-galactoside-binding lectin family and is produced by macrophages [11]. It is highly stable and resistant to hemodynamic alterations, and is an early indicator of cardiac fibrosis, ventricular remodeling, and renal impairment in HF patients; however, it has low specificity [54]. Under hypoxic or hypocaloric~~

conditions, Gal-3 expression is upregulated, through the activity of hypoxia inducible factor- 1 alpha (HIF-1α) and nuclear factor – kappa beta (NF κB); further formed Gal-3 enters systemic circulation. Gal-3 activates mitogen activated protein kinase (MAPK) signal transduction pathway, leading to cell proliferation and cell survival [6]. In the present study, serum Gal-3 levels were 24.93±10.84 ng/mL and 17.77±8.29 in groups 1 and 2 respectively (p=0.001) (Table 2). According to Gehlken et al, Gal-3 plays an important role in inflammation, fibrosis and ventricular remodeling. Gal-3 levels are independently associated with HF. Gal-3 is an early marker of fibrotic process [53]. Gal-3 is an independent predictor of mortality in patients with moderate to advanced chronic HF. Gal-3 is correlated with various collagen breakdown products [55].

In the present study, ROC curve showed AUC for Gal-3 of 0.51 with 95% CI of 0.44 to 0.58; p=0.60. The cut-off value of Gal-3 was 17.58 ng/mL with sensitivity and specificity of 55% and 57% respectively. (Table 53, Fig 3 1) In group 1, 72% and 28% of the individuals had Gal-3 values higher and lower than the cut-off value respectively. In group 2, 42% and 58% of the individuals had Gal-3 values higher and lower than the cut-off value respectively. In group 1, Gal-3 levels were higher than the cut-off only in 72% of individuals which was much lesser than that of NT-proBNP and sST2. But in group 2, individuals more than the cut-off were almost the same as that of NT-proBNP which were higher than that of sST2. That showed that sST2 and Gal-3 had almost same diagnostic potential and seem to be better than that of NT-proBNP in various aspects. Thus, in HF, the diagnostic threshold of sST2, Gal-3 and NT-proBNP may be combined for better diagnosis and risk stratification of HF especially HFpEF. According to Jiang et al, cut-off value of Gal-3 to diagnose HF is 17.8 ng/mL. Gal-3 is higher in patients with HFpEF than that in HFrEF. Gal-3 levels are associated with ECHO findings, which further substantiates that Gal-3 is useful in diagnosing HFpEF [1]. Two studies show the same Gal-3 cut-off values as that of the present study. In the DEAL study, after adjusting

for confounders, Gal-3 remains as a sole predictor with the cut-off value of 17.6ng/mL. In the COACH study (Coordinating Study Evaluating Outcomes of Advising and Counseling in Heart Failure), Gal-3 values >17.8 ng/mL are related to an increased risk of new hospitalization for HF. On the contrary, in the HF-ACTION cohort, which includes ambulatory HF patients who underwent a structured exercise program, the median Gal-3 level is 14.0 ng/mL, which is lower than the present study [~~56~~60].

In the present study, ROC curve showed AUC for microRNA210-3p of 0.79 with 95% CI of 0.68 to 0.89 with <p=0.001. The cut-off fold change expression was 5.03 with sensitivity and specificity of 87% and 54% respectively. MicroRNA210-3p had the highest sensitivity and AUC among the biomarkers analyzed in the present study. micro-210-3p could be an early diagnostic marker of HFpEF. The microRNA detection methods should ensure accuracy and precision [61]. MAGGIC score demonstrated the overall best performance with the highest AUC of 0.86 (95% CI: 0.86-0.90) (p=0.001, cut-off score of 14 with sensitivity of 95% and specificity of 89%, suggesting its reliability for risk stratification. (Table 5, Fig 3)

Widely accepted MAGGIC score showed positive correlation with NT-proBNP, sST2 and galectin-3. There was positive correlation among NT-proBNP, sST2 and galectin-3. But microRNA did not show any correlation with any of the variables probably due to small sample size. NT-proBNP and MAGGIC score showed positive correlation with HbA1c. sST2 showed positive correlation with total cholesterol and HbA1c. Total cholesterol showed positive correlation with triglyceride and LDL cholesterol and negative correlation with HDL cholesterol (Table 6).

~~In the present study on HF patients, NT proBNP was positively correlated with sST2 and Gal-3. In addition, sST2 was positively correlated with Gal-3 (Table 4).~~ Jiang et al, concluded that in HF, BNP is sensitive to volume overload, while Gal-3 is sensitive to fibrosis, hence a combination of these markers may be beneficial in diagnosis [1]. Patients with elevated levels

**Formatted:** Normal, Right: 0"

of both NPs and Gal-3 have more than twice the risk of developing HF [60~~56~~]. Cui et al, has shown that Gal-3 is superior to sST2 in distinguishing HFpEF from healthy individuals and other phenotypes of HF. However, sST2 provides incremental value to NT-proBNP in HF. Although AHA has recommended Gal-3 and sST2 to be used along with NPs for risk stratification in HF, only few data are available concerning their prognostic value in HFpEF [60~~56~~]. Since Gal-3 levels are directly related to LV end-diastolic pressure in HFpEF, elevated levels are associated with frequent hospitalizations and increased mortality [62~~57~~]. Only Gal-3 maintains strong prognostic ability after correction for clinical factors and NPs [38~~7~~]. Similar to NPs, Gal-3 is also limited by CKD with GFR <60 ml/min/1·73m$^2$. Gal-3 is useful in predicting adverse events in HFpEF, whereas sST2 is useful in HFrEF. Serum sST2 measurements provide information on myocardial fibrosis and systolic dysfunction [62~~57~~]. Combination of NT-proBNP and sST2 give an insight into NYHA states. sST2 is relatively better than NT-proBNP in the detection of HFpEF in hypertensive patients [58]. Gal-3 has few advantages over other two markers, in that it predicts HF risk in the general population as well as in the early fibrosis and ventricular remodeling in HF [63~~59~~].

~~Studies are being done to assess the diagnostic and prognostic potential of epigenetic markers such as micro-RNAs (miRNA), long non-coding RNAs, and exosome cargo, because of their potential role in the early phases of cardiac dysfunction. miRNAs play critical roles in heart development, function, and response to ischemia/reperfusion injury. There is increasing evidence that miRNAs may have a key role in the pathogenesis of HF through regulation of genes involved in adverse ventricular remodelling [60]. MiRNAs can be used alone or in combination with other biomarkers of HF such as BNP [58]. The dysregulation of miRNAs, and their target genes, are related to signaling pathways of HF, including cardiac hypertrophy, oxidative stress, cardiac fibrosis, etc [61]. The circulating miRNAs are relatively stable and easily detectable by non-invasive methodology, hence, circulating miRs may be ideal candidate~~

biomarkers and drug targets for HF. miRNAs are found to show high sensitivity and specificity in HF. However, studies show inconsistent results in the number and levels of microRNAs [61]. In response to hypoxia, specific set of microRNAs, called hypoxamirs are upregulated [60]. In the present study, microRNA 210-3p was analyzed in 40 patients of group 1 and 40 patients of group 2. Twenty apparently healthy individuals served as controls. MicroRNA 210-3p showed increased expression of 11.89-fold in group 1and 1.08-fold in group 2 compared to controls. There was statistically significant difference with p value of 0.031 (Table 3, Fig 2). According to Shen et al, the most down-regulated miRNA is miR-30c, and the most upregulated is miR-210-3p [61]. Through the HIF-1α pathway, miR-210-3p is independently associated with HF, cardiovascular mortality and cardiac regeneration. miR-210-3p is found to inhibit apoptosis of cardiomyocytes. HF patients having increased expression levels of miR-210-3p show frequent HF decompensations and adverse ventricular remodelling [60]. Lower concentrations of miR-210 are associated with clinical improvement over three-month follow-up period. Increased levels of miR-210 in advanced stage of HF are associated with NT-proBNP levels. miR-210 has a pro-fibrotic action in the infarcted heart tissue and high levels have been found in the fibroblasts and macrophages of failing hearts [62].

A better understanding of the role of miRNAs in HF may highlight molecular and cellular mechanisms of the CVD and allow to improve HF patients' management and therapies [62]. miR-210-3p, miR-22-5p, miR-22-3p, miR-21-3p, and miR-339-3p are positively correlated and miR-125a-5p is negatively correlated with NT-proBNP levels. MiRs do not show a higher diagnostic potential than other biomarkers, probably because the end-stage HF disease involves multiple organs. Since HF is multi-organ disease miRNAs may reflect an extended inflammatory state more than the original cardiac pathology [62]. In the present study, ROC curve showed AUC for microRNA210-3p of 0.79 with 95% CI of 0.68 to 0.89 with $<p=0.001$. The cut-off fold change expression was 5.03 with sensitivity and specificity of 87% and 54%

respectively. MicroRNA210-3p had the highest sensitivity and AUC among the biomarkers analyzed in the present study. MicroRNA-210-3p was positively correlated with Gal-3 in patients with HF (p=0.03) (Table 4). In group 1, 73% and 27% of the individuals had microRNA-210-3p values higher and lower than the cut-off value respectively. In group 2, 68% and 32% of the individuals had microRNA-210-3p values higher and lower than the cut-off value respectively. Since the percentage of individuals who showed higher fold changes than the cut-off value was the highest when compared to NT-proBNP, sST2 and Gal-3 especially in group 2. Hence, micro-210-3p could be an early diagnostic marker of HFpEF. As per Shen et al, in Asian countries, let-7i-5p and miR-195 are upregulated and miR-129 is downregulated; whereas in non-Asian countries, the upregulated and downregulated miRNAs are let-7e-5p and miR-30c respectively. The microRNA detection methods should ensure accuracy and precision [61].The pathogenesis of HF is highly complex, hence relying on a single biomarker will not be sufficient in diagnosing heart failure. Each biomarker has its inherent advantages and disadvantages. Thus, a multi-biomarker strategy, such that biomarkers could provide information on different pathophysiological pathways, may offer promising strategy that goes beyond the limits of the current management of HF [64]. In community-based screening, ECHO may be supplemented with NTproBNP and sST2 and Gal-3 may be done in addition to stratify the patients for effective management [65]. multiple markers could indicate various pathogenic stages of the disease which will facilitate early diagnosis of HF [49]. Along with NPs, fibrosis markers such as sST2 and Gal-3 are being suggested in the ACC)/AHA HF guidelines, for aiding risk stratification of these patients [32]. Also, it enhances the possibility of employing tailored therapies to the individuals with heterogeneous presentation. Till date, the biomarkers chosen for research are selected at random and by varied experiences of the researcher. A computer assisted approach can be used as an alternative to select the panel of biomarkers. ACC/AHA guidelines suggest that a combination of biomarkers

**Commented [SS15]:** Added

is beneficial for risk stratification. It is better to have an integrated protocol of biomarkers with clinical variables and imaging characteristics [11].

Formatted: Normal (Web), Pattern: Clear (White)

Formatted: Font: (Default) Times New Roman, 14 pt, Bold

## Strengths:

The study is carried out in India, where the resource settings are limited. India as such is highly diverse in economy, food, physical habits, literacy rate across the various states. But the study was carried out in a tertiary care hospital in a Metropolitan city. So to major extent it takes into account of the various factors. A multi-marker study in a relatively large sample size comprising of both genders remains to be major strength of the study.

Commented [SS16]: Added

~~The pathogenesis of HF is highly complex, hence relying on a single biomarker will not be sufficient in diagnosing heart failure. Each biomarker has its inherent advantages and disadvantages. Thus, multiple markers could indicate various pathogenic stages of the disease which will facilitate early diagnosis of HF [49]. Along with NPs, fibrosis markers such as sST2 and Gal-3 are being suggested in the ACC)/AHA HF guidelines, for aiding risk stratification of these patients [32]. Also, it enhances the possibility of employing tailored therapies to the individuals with heterogeneous presentation. Till date, the biomarkers chosen for research are selected at random and by varied experiences of the researcher. A computer assisted approach can be used as an alternative to select the panel of biomarkers. ACC/AHA guidelines suggest that a combination of biomarkers is beneficial for risk stratification. It is better to have an integrated protocol of biomarkers with clinical variables and imaging characteristics [11].~~

## Limitations:

The study did not include apparently healthy individuals and hence, baseline levels of healthy individuals could not be established. Also, the influence of metabolic diseases- hypertension, diabetes mellitus and obesity on HFpEF could not be established. A cohort of hypertensive and

diabetes mellitus patients could establish the cause-and-effect relationship and a better profile of markers according to the stage of the study.

# Conclusion

The biomarkers- NT-proBNP, sST2 and galectin-3 were markedly elevated in HF patients with EF less than 50% than in patients with more than 50%. Since HF is a multi-organ syndrome, other organs could also contribute to increased galectin-3 and sST2 levels in HF. The gold-standard marker- NT-proBNP showed positive association with both sST2 and galectin-3. Also, there was positive association of sST2 with galectin-3. ~~MicroRNA-210-3p showed marked increased expression in HF with EF than 50%.~~ microRNA-210-3p fold changes were positively associated with galectin-3, indicating that both the markers indicate cardiac fibrosis stage of HF. Since each marker has its specific advantages and disadvantages, multi-marker approach could help in the early diagnosis as well as in stratifying HF patients so that appropriate target therapies can be implemented according to the pathogenic stage of the disease.

# List of abbreviations

ACE: angiotensin converting enzyme

AHA/ACC: American Heart Association/ American College of Cardiology

AMI: acute myocardial infarction

AUC: area under the curve

BMI: body mass index

BNP: brain natriuretic peptide

CKD: chronic kidney disease

COACH study: Coordinating Study Evaluating Outcomes of Advising and Counseling in Heart Failure

CSI-KHFR: Cardiology Society of India-Kerala Acute Heart Failure Registry

CT: cycle threshold

CV: coefficient of variation

CVD: cardiovascular disease

ECG: electrocardiogram

ECHO: echocardiography

EF: ejection fraction

ELISA: enzyme linked immunosorbent assay

ESC: European Society of Cardiology

FC: fold change

Gal-3: Galectin-3

GFR: glomerular filtration rate

HbA1c: glycated hemoglobin A1c

HDL: high-density lipoprotein

HF: heart failure

HFmrEF: heart failure with mid-range or mildly reduced ejection fraction

HFpEF: heart failure with preserved ejection fraction

HFrEF: hear failure with reduced ejection fraction

HIF-1α: hypoxia inducible factor- 1 alpha

IL-33: interleukin-33

JNC: Joint National Committee

LDL: low-density lipoprotein

LV: left ventricle

LVH: left ventricle hypertrophy

MAGGIC: Meta-Analysis Global Group in Chronic Heart Failure

**Formatted:** Font color: Auto

MAPK: mitogen activated protein kinase

miRNA: microRNA

NF-κB: nuclear factor – kappa beta

NP: natriuretic peptides

NT-proBNP: N-terminal pro brain natriuretic peptide

NYHA: New York Heart Association

qRT-PCR: quantitative reverse transcription- polymerase chain reaction

RAAS: renin–angiotensin–aldosterone system

RNA: ribonucleic acid

ROC: receiver operating characteristics

RV: right ventricle

SCD: sudden cardiac death

sST2: soluble suppression of tumorigenicity 2

T2DM: type 2 diabetes mellitus

TGL: triglyceride

WHR: waist hip ratio

YLD: years lost due to disability

# Declarations

**Ethics approval and consent to participate**: Institutional ethics committee approval was obtained from institutional ethics committee. Ethics number: IEC-NI/19/FEB/68/09, dated 10.11.2020. Written informed consent was obtained from all the study participants.

**Consent for publication**: Not applicable

**Availability of data and materials**: The authors confirm that the data supporting the findings of this study are available within the article. Raw data were generated at the location of research, Sri Ramachandra Institute of Higher Education and Research, Chennai, India. No supplementary material is available with this research.

**Commented [SS17]:** Reframed

**Conflict of interest:** The authors declare that there are no conflicts of interests.

**Funding declaration**: Ms. Jasmine Chandra A is the PhD scholar who received Founder-Chancellor Sri N.P.V. Ramasamy Udayar Research Fellowship, from Sri Ramachandra Institute of Higher Education and Research (Deemed to be University) for the Full-Time PhD program for a period of three years from October 2018 to September 2021.

## Authors contributions:

All the authors contributed equally in conceptualisation, data curation, formal analysis, funding acquisition, investigation, methodology, project administration, resources, software, supervision, validation, visualisation, writing – original draft, and writing– review & editing.

**Acknowledgements:** The authors wish to thank the Department of Cardiology and the patients for effective completion of the study.

# References


1. Jiang J, Yang B, Sun Y, Jin J, Zhao Z, Chen S. Diagnostic value of serum concentration of galectin-3 in patients with heart failure with preserved ejection fraction. Front Cardiovasc Med. 2022;8:829151.
2. Lippi G, Sanchis-Gomar F. Global epidemiology and future trends of heart failure. AME medical journal. 2020;5.
3. Shrivastava A, Haase T, Zeller T, Schulte C. Biomarkers for heart failure prognosis: proteins, genetic scores and non-coding RNAs. Frontiers in cardiovascular medicine. 2020;7:601364.
4. Guo Y, Lip GY, Banerjee A. Heart failure in East Asia. Curr Cardiol Rev. 2013;9(2):112-22.
5. Huffman MD, Prabhakaran D. Heart failure: epidemiology and prevention in India. Natl Med J India. 2010;23(5):283-8.

6. Ponikowski P, Voors AA, Anker SD, Bueno H, Cleland JGF, Coats AJS, et al. ESC Scientific Document Group. 2016 ESC Guidelines for the diagnosis and treatment of acute and chronic heart failure: The Task Force for the diagnosis and treatment of acute and chronic heart failure of the European Society of Cardiology (ESC) Developed with the special contribution of the Heart Failure Association (HFA) of the ESC. Eur Heart J. 2016;37(27):2129-2200.
7. McDonagh TA, Metra M, Adamo M, Gardner RS, Baumbach A, Böhm M, et al. 2021 ESC Guidelines for the diagnosis and treatment of acute and chronic heart failure: Developed by the Task Force for the diagnosis and treatment of acute and chronic heart failure of the European Society of Cardiology (ESC) With the special contribution of the Heart Failure Association (HFA) of the ESC. European heart journal. 2021;42(36):3599-726.
8. Simmonds SJ, Cuijpers I, Heymans S, Jones EAV. Cellular and molecular differences between HFpEF and HFrEF: A step ahead in an improved pathological understanding. Cells. 2020;9(1):242.
9. Mahmood SS, Wang TJ. The epidemiology of congestive heart failure: the Framingham Heart Study perspective. Glob Heart. 2013;8(1):77-82.
10. Savarese G, Lund LH. Global public health burden of heart failure. Card Fail Rev. 2017;3(1):7-11.
11. Castiglione V, Aimo A, Vergaro G, Saccaro L, Passino C, Emdin M. Biomarkers for the diagnosis and management of heart failure. Heart Fail Rev. 2022;27(2):625-43.
12. Dwlorok V, Hähnel V, Bannehr M, Paar V, Edlinger C, Lichtenauer M, et al. soluble suppression of tumorigenicity 2 (sST2) in patients with predominantly decompensated right heart Failure- a prospective observational study. J Clin Med. 2023;12(23):7200.


**Formatted:** Font: (Default) Times New Roman, 12 pt, Font color: Custom Color(RGB(34,34,34))

**Formatted:** Font: (Default) Times New Roman, 12 pt

**Formatted:** Font: (Default) Times New Roman, 12 pt, Font color: Custom Color(RGB(33,33,33))

**Formatted:** Font: (Default) Times New Roman, 12 pt

**Formatted:** Font: (Default) Times New Roman, 12 pt, Font color: Custom Color(RGB(33,33,33))

**Formatted:** Font: (Default) Times New Roman, 12 pt

**Formatted:** Font: (Default) Times New Roman, 12 pt, Font color: Custom Color(RGB(33,33,33))

**Formatted:** Font: (Default) Times New Roman, 12 pt

**Formatted:** Font: (Default) Times New Roman, 12 pt, Font color: Custom Color(RGB(33,33,33))

**Formatted:** Font: (Default) Times New Roman, 12 pt

**Formatted:** Font: (Default) Times New Roman, 12 pt, Font color: Custom Color(RGB(33,33,33))

**Formatted:** Font: (Default) Times New Roman, 12 pt

13. Villacorta H, Maisel AS. Soluble ST2 testing: a promising biomarker in the management of heart failure. Arq Bras Cardiol. 2016;106(2):145-52.

14. Merino-Merino A, Gonzalez-Bernal J, Fernandez-Zoppino D, Saez-Maleta R, Perez-Rivera JA. The role of galectin-3 and ST2 in cardiology: a short review. Biomolecules. 2021;11(8):1167.

15. Gu1an Y, Song X, Sun W, Wang Y, Liu B. Effect of hypoxia-induced microRNA-210 expression on cardiovascular disease and the underlying mechanism. Oxid Med Cell Longev. 2019;2019:4727283.

16. Spinar J, Spinarova L, Malek F, Ludka O, Krejci J, Ostadal P, et al. Prognostic value of NT-proBNP added to clinical parameters to predict two-year prognosis of chronic heart failure patients with mid-range and reduced ejection fraction–A report from FAR NHL prospective registry. PloS one. 2019 Mar 26;14(3):e0214363.

17. Triposkiadis F, Starling RC. Chronic heart failure: diagnosis and management beyond LVEF classification. J Clin Med. 2022;11(6):1718.

18. Chengode S. Left ventricular global systolic function assessment by echocardiography. Ann Card Anaesth. 2016;19(Suppl):S26-34.

19. Karmacharya P, Shrestha GL, Singh S, Shrestha OK. Relation of waist hip ratio and body mass index with the vital capacity. JCMC. 2019; 9(29):51-5.

20. Chobanian AV, Bakris GL, Black HR, Cushman WC, Green LA, Izzo JL Jr, et al. Joint National Committee on prevention, detection, evaluation, and treatment of high blood pressure. National Heart, Lung, and Blood Institute; National High Blood Pressure Education Program Coordinating Committee. Seventh report of the Joint National Committee on prevention, detection, evaluation, and treatment of high blood pressure. Hypertension. 2003;42(6):1206-52.


**Formatted:** Font: (Default) Times New Roman, 12 pt, Font color: Custom Color(RGB(33,33,33))

**Formatted:** Font: (Default) Times New Roman, 12 pt

**Formatted:** Font: (Default) Times New Roman, 12 pt, Font color: Custom Color(RGB(33,33,33))

**Formatted:** Font: (Default) Times New Roman, 12 pt

**Formatted:** Font: (Default) Times New Roman, 12 pt, Font color: Custom Color(RGB(33,33,33))

**Formatted:** Font: (Default) Times New Roman, 12 pt

**Formatted:** Font: (Default) Times New Roman, 12 pt, Font color: Custom Color(RGB(33,33,33))

**Formatted:** Font: (Default) Times New Roman, 12 pt

**Formatted:** Font: (Default) Times New Roman, 12 pt, Font color: Custom Color(RGB(33,33,33))

**Formatted:** Font: (Default) Times New Roman, 12 pt

21. International Expert Committee. International Expert Committee report on the role of the A1C assay in the diagnosis of diabetes. Diabetes Care. 2009;32(7):1327-34.

22. Pocock SJ, Ariti CA, McMurray JJ, Maggioni A, Kober L, Squire IB, Swedberg K, Dobson J, Poppe KK, Whalley GA, Doughty RN. Predicting survival in heart failure: a risk score based on 39 372 patients from 30 studies. *Eur Heart J*. 2013;34:1404–1413.

~~16.~~

~~17.~~23. Corteville DC, Bibbins-Domingo K, Wu AH, Ali S, Schiller NB, Whooley MA. N-terminal pro-B-type natriuretic peptide as a diagnostic test for ventricular dysfunction in patients with coronary disease: data from the heart and soul study. Arch Intern Med. 2007;167(5):483-9.

~~18.~~24. Wang Z, Pan X, Xu H, Wu Y, Jia X, Fang Y, et al. Serum soluble ST2 is a valuable prognostic biomarker in patients with acute heart failure. Front Cardiovasc Med. 2022;9:812654.

~~19.~~25. de Boer RA, Lok DJ, Jaarsma T, van der Meer P, Voors AA, Hillege HL, et al. Predictive value of plasma galectin-3 levels in heart failure with reduced and preserved ejection fraction. Ann Med. 2011;43(1):60-8.

~~20.~~26. Available from: https://www.helini.in/uploads/3/0/9/5/30951267/purefast_microrna_mini_spin_prep_kit.pdf

~~21. Triposkiadis F, Starling RC. Chronic heart failure: diagnosis and management beyond LVEF classification. J Clin Med. 2022;11(6):1718.~~

~~22. Chengode S. Left ventricular global systolic function assessment by echocardiography. Ann Card Anaesth. 2016;19(Suppl):S26-34.~~


**Formatted:** Font: (Default) Times New Roman, 12 pt, Font color: Custom Color(RGB(33,33,33))

**Formatted:** Font: (Default) Times New Roman, 12 pt

**Formatted:** Font: (Default) Times New Roman

**Formatted:** Font: (Default) Times New Roman, 12 pt

**Formatted:** Normal, No bullets or numbering

**Formatted:** Font: (Default) Times New Roman, 12 pt, Font color: Custom Color(RGB(33,33,33))

**Formatted:** Numbered + Level: 1 + Numbering Style: 1, 2, 3, … + Start at: 1 + Alignment: Left + Aligned at: 0.25" + Indent at: 0.5"

**Formatted:** Font: (Default) Times New Roman, 12 pt

**Formatted:** Font: (Default) Times New Roman, 12 pt, Font color: Custom Color(RGB(33,33,33))

**Formatted:** Font: (Default) Times New Roman, 12 pt

**Formatted:** Font: (Default) Times New Roman, 12 pt, Font color: Custom Color(RGB(33,33,33))

**Formatted:** Font: (Default) Times New Roman, 12 pt

**Formatted:** Font: (Default) Times New Roman, 12 pt

**Formatted:** Font: (Default) Times New Roman, 12 pt, Font color: Custom Color(RGB(33,33,33))

**Formatted:** Normal, No bullets or numbering

**Formatted:** Font: (Default) Times New Roman, 12 pt

**Formatted:** Font: (Default) Times New Roman, 12 pt, Font color: Custom Color(RGB(33,33,33))

**Formatted:** Font: (Default) Times New Roman, 12 pt

~~23. Karmacharya P, Shrestha GL, Singh S, Shrestha OK. Relation of waist hip ratio and body mass index with the vital capacity. JCMC. 2019; 9(29):51-5.~~

~~24. Chobanian AV, Bakris GL, Black HR, Cushman WC, Green LA, Izzo JL Jr, et al. Joint National Committee on prevention, detection, evaluation, and treatment of high blood pressure. National Heart, Lung, and Blood Institute; National High Blood Pressure Education Program Coordinating Committee. Seventh report of the Joint National Committee on prevention, detection, evaluation, and treatment of high blood pressure. Hypertension. 2003;42(6):1206-52.~~

~~25. International Expert Committee. International Expert Committee report on the role of the A1C assay in the diagnosis of diabetes. Diabetes Care. 2009;32(7):1327-34.~~

~~26.~~27. Benjamin EJ, Blaha MJ, Chiuve SE, Cushman M, Das SR, Deo R, et al. American Heart Association Statistics Committee and Stroke Statistics Subcommittee. Heart Disease and Stroke Statistics-2017 Update: a report from the American Heart Association. Circulation. 2017;135(10):e146-603.

~~27.~~28. Joseph S, Panniyammakal J, Abdullakutty J, S S, Vaikathuseril L J, Joseph J, et al. The Cardiology Society of India-Kerala Acute Heart Failure Registry: poor adherence to guideline-directed medical therapy. Eur Heart J. 2021:ehab793.

~~28.~~29. Kumar AS, Sinha N. Cardiovascular disease in India: A 360 degree overview. Med J Armed Forces India. 2020;76(1):1-3.

~~29.~~30. Teramoto K, Teng TK, Chandramouli C, Tromp J, Sakata Y, Lam CS. Epidemiology and clinical features of heart failure with preserved ejection fraction. Card Fail Rev. 2022;8:e27.

~~30.~~31. Jarab AS, Al-Qerem W, Hamam H, Abu Heshmeh S, Mukattash TL, Alefishat EA. Factors associated with lipid control in outpatients with heart failure. Front Cardiovasc Med. 2023;10:1153310.

**Formatted:** Font: (Default) Times New Roman, 12 pt, Font color: Custom Color(RGB(33,33,33))

**Formatted:** Font: (Default) Times New Roman, 12 pt

**Formatted:** Font: (Default) Times New Roman, 12 pt, Font color: Custom Color(RGB(33,33,33))

**Formatted:** Numbered + Level: 1 + Numbering Style: 1, 2, 3, … + Start at: 1 + Alignment: Left + Aligned at: 0.25" + Indent at: 0.5"

**Formatted:** Font: (Default) Times New Roman, 12 pt

**Formatted:** Font: (Default) Times New Roman, 12 pt, Font color: Custom Color(RGB(33,33,33))

**Formatted:** Font: (Default) Times New Roman, 12 pt

**Formatted:** Font: (Default) Times New Roman, 12 pt, Font color: Custom Color(RGB(33,33,33))

**Formatted:** Font: (Default) Times New Roman, 12 pt

**Formatted:** Font: (Default) Times New Roman, 12 pt, Font color: Custom Color(RGB(33,33,33))

**Formatted:** Font: (Default) Times New Roman, 12 pt

**Formatted:** Font: (Default) Times New Roman, 12 pt, Font color: Custom Color(RGB(33,33,33))

**Formatted:** Font: (Default) Times New Roman, 12 pt

~~31.~~32. Yao YS, Li TD, Zeng ZH. Mechanisms underlying direct actions of hyperlipidemia on myocardium: an updated review. Lipids Health Dis. 2020;19(1):23.

~~32.~~33. Piek A, Du W, de Boer RA, Silljé HHW. Novel heart failure biomarkers: why do we fail to exploit their potential? Crit Rev Clin Lab Sci. 2018;55(4):246-63.

~~33.~~34. Panagopoulou V, Deftereos S, Kossyvakis C, Raisakis K, Giannopoulos G, Bouras G, et al. NT-proBNP: an important biomarker in cardiac diseases. Curr Top Med Chem. 2013;13(2):82-94.

~~34.~~35. Çelik A, Kılıçaslan B, Temizhan A, Güvenç TS, Altay H, Çavuşoğlu Y, et al. How to use natriuretic peptides in patients with heart failure with non-reduced ejection fraction? Anatol J Cardiol. 2023;27(6):308-18.

~~35.~~36. Volpe M, Carnovali M, Mastromarino V. The natriuretic peptides system in the pathophysiology of heart failure: from molecular basis to treatment. Clin Sci (Lond). 2016;130(2):57-77.

~~36.~~37. Kang SH, Park JJ, Choi DJ, Yoon CH, Oh IY, Kang SM, et al. KorHF Registry. Prognostic value of NT-proBNP in heart failure with preserved versus reduced EF. Heart. 2015;101(23):1881-8.

~~37.~~38. Patel AN, Southern WN. BNP-Response to acute heart failure treatment identifies high-risk population. Heart Lung Circ. 2020;29(3):354-60.

~~38.~~39. Ponikowski P, Voors AA, Anker SD, Bueno H, Cleland JGF, Coats AJS, et al. ESC Scientific Document Group. 2016 ESC Guidelines for the diagnosis and treatment of acute and chronic heart failure: The Task Force for the diagnosis and treatment of acute and chronic heart failure of the European Society of Cardiology (ESC)Developed with the special contribution of the Heart Failure Association (HFA) of the ESC. Eur Heart J. 2016;37(27):2129-200.

**Formatted:** Font: (Default) Times New Roman, 12 pt, Font color: Custom Color(RGB(33,33,33))
**Formatted:** Font: (Default) Times New Roman, 12 pt
**Formatted:** Font: (Default) Times New Roman, 12 pt, Font color: Custom Color(RGB(33,33,33))
**Formatted:** Font: (Default) Times New Roman, 12 pt
**Formatted:** Font: (Default) Times New Roman, 12 pt, Font color: Custom Color(RGB(33,33,33))
**Formatted:** Font: (Default) Times New Roman, 12 pt
**Formatted:** Font: (Default) Times New Roman, 12 pt, Font color: Custom Color(RGB(33,33,33))
**Formatted:** Font: (Default) Times New Roman, 12 pt
**Formatted:** Font: (Default) Times New Roman, 12 pt, Font color: Custom Color(RGB(33,33,33))
**Formatted:** Font: (Default) Times New Roman, 12 pt
**Formatted:** Font: (Default) Times New Roman, 12 pt, Font color: Custom Color(RGB(33,33,33))
**Formatted:** Font: (Default) Times New Roman, 12 pt
**Formatted:** Font: (Default) Times New Roman, 12 pt, Font color: Custom Color(RGB(33,33,33))
**Formatted:** Font: (Default) Times New Roman, 12 pt
**Formatted:** Font: (Default) Times New Roman, 12 pt, Font color: Custom Color(RGB(33,33,33))
**Formatted:** Font: (Default) Times New Roman, 12 pt

~~39.~~40. Cao Z, Jia Y, Zhu B. BNP and NT-proBNP as diagnostic biomarkers for cardiac dysfunction in both clinical and forensic medicine. Int J Mol Sci. 2019;20(8):1820.

~~40. Yancy CW, Jessup M, Bozkurt B, Butler J, Casey DE Jr, Drazner MH, et al. American College of Cardiology Foundation/American Heart Association Task Force on Practice Guidelines. 2013 ACCF/AHA guideline for the management of heart failure: a report of the American College of Cardiology Foundation/American Heart Association Task Force on practice guidelines. Circulation. 2013;128(16):e240-327.~~

41. Welsh P, Campbell RT, Mooney L, Kimenai DM, Hayward C, Campbell A, et al. Reference ranges for NT-proBNP (N-terminal pro-B-type natriuretic peptide) and risk factors for higher NT-proBNP concentrations in a large general population cohort. Circ Heart Fail. 2022;15(10):e009427.

42. Singh S, Pandey A, Neeland IJ. Diagnostic and prognostic considerations for use of natriuretic peptides in obese patients with heart failure. Prog Cardiovasc Dis. 2020;63(5):649-55.

43. Anwaruddin S, Lloyd-Jones DM, Baggish A, Chen A, Krauser D, Tung R, et al. Renal fun4ction, congestive heart failure, and amino-terminal pro-brain natriuretic peptide measurement: results from the ProBNP Investigation of Dyspnea in the Emergency Department (PRIDE) Study. J Am Coll Cardiol. 2006 Jan 3;47(1):91-7.

44. Wettersten N, Horiuchi Y, Maisel A. Advancements in biomarkers for cardiovascular disease: diagnosis, prognosis, and therapy. Fac Rev. 2021;10:34.

45. Lotierzo M, Dupuy AM, Kalmanovich E, Roubille F, Cristol JP. sST2 as a value-added biomarker in heart failure. Clin Chim Acta. 2020;501:120-30.

46. Aimo A, Januzzi JL Jr, Bayes-Genis A, Vergaro G, Sciarrone P, Passino C, et al. Clinical and prognostic significance of sST2 in heart failure: JACC review topic of the week. J Am Coll Cardiol. 2019;74(17):2193-203.


**Formatted:** Font: (Default) Times New Roman, 12 pt, Font color: Custom Color(RGB(33,33,33))

**Formatted:** Font: (Default) Times New Roman, 12 pt

**Formatted:** Font: (Default) Times New Roman, 12 pt, Font color: Custom Color(RGB(33,33,33))

**Formatted:** Normal, No bullets or numbering

**Formatted:** Font: (Default) Times New Roman, 12 pt

**Formatted:** Font: (Default) Times New Roman, 12 pt, Font color: Custom Color(RGB(33,33,33))

**Formatted:** Numbered + Level: 1 + Numbering Style: 1, 2, 3, … + Start at: 1 + Alignment: Left + Aligned at: 0.25" + Indent at: 0.5"

**Formatted:** Font: (Default) Times New Roman, 12 pt

**Formatted:** Font: (Default) Times New Roman, 12 pt, Font color: Custom Color(RGB(33,33,33))

**Formatted:** Font: (Default) Times New Roman, 12 pt

**Formatted:** Font: (Default) Times New Roman, 12 pt, Font color: Custom Color(RGB(33,33,33))

**Formatted:** Font: (Default) Times New Roman, 12 pt

**Formatted:** Font: (Default) Times New Roman, 12 pt, Font color: Custom Color(RGB(33,33,33))

**Formatted:** Font: (Default) Times New Roman, 12 pt

**Formatted:** Font: (Default) Times New Roman, 12 pt, Font color: Custom Color(RGB(33,33,33))

**Formatted:** Font: (Default) Times New Roman, 12 pt

**Formatted:** Font: (Default) Times New Roman, 12 pt, Font color: Custom Color(RGB(33,33,33))

**Formatted:** Font: (Default) Times New Roman, 12 pt

47. Firouzabadi N, Dashti M, Dehshahri A, Bahramali E. Biomarkers of IL-33 and sST2 and lack of association with carvedilol therapy in heart failure. Clin Pharmacol. 2020;12:53-8.

48. Devine PA, Cardwell C, Maxwell AP. Association of soluble ST2 with all-cause and cardiovascular mortality in renal transplant recipients: a single-centre cohort study. BMC Nephrol. 2020;21(1):22.

49. Aleksova A, Paldino A, Beltrami AP, Padoan L, Iacoviello M, Sinagra G, et al. Cardiac biomarkers in the Emergency Department: the role of soluble ST2 (sST2) in acute heart failure and acute coronary syndrome-there is meat on the bone. J Clin Med. 2019;8(2):270.

50. Bai J, Han L, Liu H. Combined use of high-sensitivity ST2 and NT-proBNP for predicting major adverse cardiovascular events in coronary heart failure. Ann Palliat Med. 2020;9(4):1976-89.

51. Janjusevic M, Fluca AL, Ferro F, Gagno G, D'Alessandra Y, Beltrami AP, et al. Traditional and emerging biomarkers in asymptomatic left ventricular dysfunction-promising non-coding RNAs and exosomes as biomarkers in early phases of cardiac damage. Int J Mol Sci. 2021;22(9):4937.

52. Blanda V, Bracale UM, Di Taranto MD, Fortunato G. Galectin-3 in cardiovascular diseases. Int J Mol Sci. 2020;21(23):9232.

53. Gehlken C, Suthahar N, Meijers WC, de Boer RA. Galectin-3 in heart failure: an update of the last 3 years. Heart Fail Clin. 2018;14(1):75-92.

54. Amin HZ, Amin LZ, Wijaya IP. Galectin-3: a novel biomarker for the prognosis of heart failure. Clujul Med. 2017;90(2):129-32.


**Formatted:** Font: (Default) Times New Roman, 12 pt, Font color: Custom Color(RGB(33,33,33))

**Formatted:** Font: (Default) Times New Roman, 12 pt

**Formatted:** Font: (Default) Times New Roman, 12 pt, Font color: Custom Color(RGB(33,33,33))

**Formatted:** Font: (Default) Times New Roman, 12 pt

**Formatted:** Font: (Default) Times New Roman, 12 pt, Font color: Custom Color(RGB(33,33,33))

**Formatted:** Font: (Default) Times New Roman, 12 pt

**Formatted:** Font: (Default) Times New Roman, 12 pt, Font color: Custom Color(RGB(33,33,33))

**Formatted:** Font: (Default) Times New Roman, 12 pt

**Formatted:** Font: (Default) Times New Roman, 12 pt, Font color: Custom Color(RGB(33,33,33))

**Formatted:** Font: (Default) Times New Roman, 12 pt

**Formatted:** Font: (Default) Times New Roman, 12 pt, Font color: Custom Color(RGB(33,33,33))

**Formatted:** Font: (Default) Times New Roman, 12 pt

**Formatted:** Font: (Default) Times New Roman, 12 pt, Font color: Custom Color(RGB(33,33,33))

**Formatted:** Font: (Default) Times New Roman, 12 pt

**Formatted:** Font: (Default) Times New Roman, 12 pt, Font color: Custom Color(RGB(33,33,33))

**Formatted:** Font: (Default) Times New Roman, 12 pt

55. Zaborska B, Sikora-Frąc M, Smarż K, Pilichowska-Paszkiet E, Budaj A, Sitkiewicz D, et al. The role of galectin-3 in heart failure-the diagnostic, prognostic and therapeutic potential-where do we stand? Int J Mol Sci. 2023;24(17):13111.

56. Zhou SS, Jin JP, Wang JQ, Zhang ZG, Freedman JH, Zheng Y, et al. miRNAS in cardiovascular diseases: potential biomarkers, therapeutic targets and challenges. Acta Pharmacol Sin. 2018;39(7):1073-84.

57. Shen NN, Wang JL, Fu YP. The microRNA expression profiling in heart failure: a systematic review and meta-analysis. Front Cardiovasc Med. 2022;9:856358.

58. Felker GM, Fiuzat M, Shaw LK, Clare R, Whellan DJ, Bettari L, et al. Galectin-3 in ambulatory patients with heart failure: results from the HF-ACTION study. Circ Heart Fail. 2012;5(1):72-8.

59. Yancy CW, Jessup M, Bozkurt B, Butler J, Casey DE Jr, Drazner MH, et al. American College of Cardiology Foundation/American Heart Association Task Force on Practice Guidelines. 2013 ACCF/AHA guideline for the management of heart failure: a report of the American College of Cardiology Foundation/American Heart Association Task Force on practice guidelines. Circulation. 2013;128(16):e240-327.

~~56.~~60. Shi Y, Liu J, Liu C, Shuang X, Yang C, Qiao W, et al. Diagnostic and prognostic value of serum soluble suppression of tumorigenicity-2 in heart failure with preserved ejection fraction: a systematic review and meta-analysis. Front Cardiovasc Med. 2022;9:937291.

61. Galluzzo A, Gallo S, Pardini B, Birolo G, Fariselli P, Boretto P, et al. Identification of novel circulating microRNAs in advanced heart failure by next-generation sequencing. ESC Heart Fail. 2021;8(4):2907-19.

**Formatted:** Font: (Default) Times New Roman, 12 pt, Font color: Custom Color(RGB(33,33,33))

**Formatted:** Font: (Default) Times New Roman, 12 pt

**Formatted:** Font: (Default) Times New Roman, 12 pt, Font color: Custom Color(RGB(33,33,33))

**Formatted:** Font: (Default) Times New Roman, 12 pt

**Formatted:** Font: (Default) Times New Roman, 12 pt, Font color: Custom Color(RGB(33,33,33))

**Formatted:** Font: (Default) Times New Roman, 12 pt

**Formatted:** Font: (Default) Times New Roman, 12 pt, Font color: Custom Color(RGB(33,33,33))

**Formatted:** Font: (Default) Times New Roman, 12 pt

**Formatted:** Font: (Default) Times New Roman, 12 pt, Font color: Custom Color(RGB(33,33,33))

**Formatted:** Font: (Default) Times New Roman, 12 pt

**Formatted:** Font: (Default) Times New Roman, 12 pt, Font color: Custom Color(RGB(33,33,33))

**Formatted:** Font: (Default) Times New Roman, 12 pt

~~57.~~62. Cui Y, Qi X, Huang A, Li J, Hou W, Liu K. Differential and predictive value of galectin-3 and soluble suppression of tumorigenicity-2 (sST2) in heart failure with preserved ejection fraction. Med Sci Monit. 2018;24:5139-46.

~~58. Felker GM, Fiuzat M, Shaw LK, Clare R, Whellan DJ, Bettari L, et al. Galectin-3 in ambulatory patients with heart failure: results from the HF-ACTION study. Circ Heart Fail. 2012;5(1):72-8.~~

~~59.~~63. ~~R~~Rincón LM, Rodríguez-Serrano M, Conde E, Lanza VF, Sanmartín M, González-Portilla P, et al. Serum microRNAs are key predictors of long-term heart failure and cardiovascular death after myocardial infarction. ESC Heart Fail. 2022;9(5):3367-79.

~~60. Zhou SS, Jin JP, Wang JQ, Zhang ZG, Freedman JH, Zheng Y, et al. miRNAS in cardiovascular diseases: potential biomarkers, therapeutic targets and challenges. Acta Pharmacol Sin. 2018;39(7):1073-84.~~

~~61. Shen NN, Wang JL, Fu YP. The microRNA expression profiling in heart failure: a systematic review and meta-analysis. Front Cardiovasc Med. 2022;9:856358.~~

64. ~~Galluzzo A, Gallo S, Pardini B, Birolo G, Fariselli P, Boretto P, et al. Identification of novel circulating microRNAs in advanced heart failure by next-generation sequencing. ESC Heart Fail. 2021;8(4):2907-19.~~ Salzano A, Marra AM, D'Assante R, Arcopinto M, Bossone E, Suzuki T, Cittadini A. Biomarkers and Imaging: Complementary or Subtractive? Heart Fail Clin. 2019 Apr;15(2):321-331.

65. Salzano A, D'Assante R, Israr MZ, Eltayeb M, D'Agostino A, Bernieh D, De Luca M, Rega S, Ranieri B, Mauro C, Bossone E, Squire IB, Suzuki T, Marra AM. Biomarkers in Heart Failure: Clinical Insights. Heart Fail Clin. 2021 Apr;17(2):223-243.

~~62.~~

**Formatted:** Font: (Default) Times New Roman, 12 pt, Font color: Custom Color(RGB(33,33,33))

**Formatted:** Font: (Default) Times New Roman, 12 pt

**Formatted:** Font: (Default) Times New Roman, 12 pt, Font color: Custom Color(RGB(33,33,33))

**Formatted:** Normal, No bullets or numbering

**Formatted:** Font: (Default) Times New Roman, 12 pt

**Formatted:** Font: (Default) Times New Roman, 12 pt, Font color: Custom Color(RGB(33,33,33))

**Formatted:** Numbered + Level: 1 + Numbering Style: 1, 2, 3, … + Start at: 1 + Alignment: Left + Aligned at: 0.25" + Indent at: 0.5"

**Formatted:** Font: (Default) Times New Roman, 12 pt

**Formatted:** Font: (Default) Times New Roman, 12 pt, Font color: Custom Color(RGB(33,33,33))

**Formatted:** Normal, No bullets or numbering

**Formatted:** Font: (Default) Times New Roman, 12 pt, Font color: Custom Color(RGB(33,33,33))

**Formatted:** Font color: Custom Color(RGB(33,33,33))

**Formatted:** Numbered + Level: 1 + Numbering Style: 1, 2, 3, … + Start at: 1 + Alignment: Left + Aligned at: 0.25" + Indent at: 0.5"

**Formatted:** Normal (Web), Justified, Numbered + Level: 1 + Numbering Style: 1, 2, 3, … + Start at: 1 + Alignment: Left + Aligned at: 0.25" + Indent at: 0.5"

**Formatted:** Strikethrough

**Formatted:** Font: (Default) Times New Roman, 14 pt

**Formatted:** Normal, No bullets or numbering

**The authors are grateful for all the suggestions raised by the reviewers and editors. Herewith we are attaching the responses to the queries. We have taken utmost care and responsibility in addressing the queries. Once again thank you very much.**

**<u>Responses to Journal requirements</u>:** 14-09-2024

1. Please ensure that your manuscript meets PLOS ONE's style requirements, including those for file naming.

   **Response: The manuscript has been modified according to the PLOS ONE's requirements.**

2. When completing the data availability statement of the submission form, you indicated that you will make your data available on acceptance. We strongly recommend all authors decide on a data sharing plan before acceptance, as the process can be lengthy and hold up publication timelines. Please note that, though access restrictions are acceptable now, your entire data will need to be made freely accessible if your manuscript is accepted for publication. This policy applies to all data except where public deposition would breach compliance with the protocol approved by your research ethics board. If you are unable to adhere to our open data policy, please kindly revise your statement to explain your reasoning and we will seek the editor's input on an exemption. Please be assured that, once you have provided your new statement, the assessment of your exemption will not hold up the peer review process.

   **Response: The authors confirm that the data supporting the findings of this study are available within the article. Raw data were generated at the location of research, Sri Ramachandra Institute of Higher Education and Research, Chennai, India. No supplementary material is available with this research.**

3. Please remove your figures from within your manuscript file, leaving only the individual TIFF/EPS image files, uploaded separately. These will be automatically included in the reviewers' PDF.

   **Responses: Figures are removed from the manuscript file and uploaded separately as TIFF files.**

**Response to the reviewers comments:**

1. Is the manuscript technically sound, and do the data support the conclusions?

The manuscript must describe a technically sound piece of scientific research with data that supports the conclusions. Experiments must have been conducted rigorously, with appropriate controls, replication, and sample sizes. The conclusions must be drawn appropriately based on the data presented.

Reviewer #1: Yes

Reviewer #2: Partly

**Response: The manuscript has been edited with the responses to the reviewers, which we think could improve the content and presentation of the manuscript.**

2. Has the statistical analysis been performed appropriately and rigorously?

Reviewer #1: Yes

Reviewer #2: No

**Response: Correlation studies and ROC curve have been modified. The information is added in the methodology, results, discussion and conclusion.**

3. Have the authors made all data underlying the findings in their manuscript fully available?

The PLOS Data policy requires authors to make all data underlying the findings described in their manuscript fully available without restriction, with rare exception (please refer to the Data Availability Statement in the manuscript PDF file). The data should be provided as part of the manuscript or its supporting information, or deposited to a public repository. For example, in addition to summary statistics, the data points behind means, medians and variance measures should be available. If there are restrictions on publicly sharing data—e.g. participant privacy or use of data from a third party—those must be specified.

Reviewer #1: Yes

Reviewer #2: Yes

4. Is the manuscript presented in an intelligible fashion and written in standard English?

PLOS ONE does not copyedit accepted manuscripts, so the language in submitted articles must be clear, correct, and unambiguous. Any typographical or grammatical errors should be corrected at revision, so please note any specific errors here.

Reviewer #1: Yes

Reviewer #2: Yes

5. Review Comments to the Author

Please use the space provided to explain your answers to the questions above. You may also include additional comments for the author, including concerns about dual publication, research ethics, or publication ethics. (Please upload your review as an attachment if it exceeds 20,000 characters)

Reviewer #1: The authors reported that NT-proBNP, sST2 and galectin-3 were significantly higher in HFrEF compared to that of HFpEF. Also, microRNA-210-3p showed significantly increased expression in HFpEF compared to controls. The findings seem to be interesting, however, I would like to make some comments.

1. Major concerns.
   - The lack of the novelty is the concern that I would like to express around the article, while data for miR-210-3p deserve to be published.

**Response: The study was intended to be conducted since NT-proBNP levels do not correlate with the severity of disease especially in cases of HFpEF. Hence, we researched to find out whether sST2 and Gal-3 could be able to diagnose those cases which were not identified by NT-proBNP. Also, microRNAs could serve as very early biomarkers of the disease. Like we do profile of certain biomarkers in case of liver or renal diseases, a profile of biomarkers in HF will reduce the chances of morbidity and mortality. Of late women tend to develop hypertension almost similar to that of men. Studies show that women respond poorly to antihypertensives compared to men. Hence, the chances of increasing cases of HFpEF is high in future. Keeping all these in mind, we proceeded with the study.**

- The authors did not validate the findings and the comparisons with widely used scores (MAGGIC, Barcelona-HF, etc.) were not performed

**Methodology: The Meta-Analysis Global Group in Chronic Heart Failure (MAGGIC) mortality risk score was calculated in all the study participants. The MAGGIC score consists of thirteen clinical predictor variables such as age, sex, body mass index (BMI), systolic blood pressure (SBP), ejection fraction (EF), serum creatinine level, participant is a current smoker, has diabetes mellitus, and chronic obstructive pulmonary disease, belongs to New York Heart Association (NYHA) class, heart failure (HF) diagnosed for at least eighteen months, use of beta-blockers and angiotensin-converting enzyme (ACE) inhibitors.**

2. Minor concerns
- Structure of the paper is not logic. Section Results contains a paragraph for B-mode Echo, while it could replaced to the subsection Methods. Please, check and correct.

**Response: The following removed in results and added in methodology**
**The study participants were subjected to the transthoracic 2D doppler echocardiography using Phillips and GE Healthcare echocardiography with patients lying supine in theleft lateral decubitus position. EF values in healthy individuals and in heart failure patients vary with ethnicity and type of ECHO instrument. According to the definition of European and United States guidelines, the normal EF is 52-72% in men and 54-74% in women [21]. According to Chengode, EF > 55% is considered to be normal. It can be 45%–54%, 30%–44% and < 30% in mild, moderate and severe LV dysfunction respectively [22].**
**Anthropometric characteristics such as body mass index (BMI) in Kg/m$^2$ and waist to hip ratio (WHR) were measured as per the Asia-Pacific guidelines [23]. Systolic and diastolic blood pressures were measured using standard procedure and patients were identified as hypertensives based on the JNC7 blood pressure classification [24]. According to International Expert Committee, diabetes mellitus was diagnosed based on glycated hemoglobin (HbA1c) ≥6.5%, fasting plasma glucose ≥126 mg/dL and 2-hour plasma glucose or random plasma glucose ≥200 mg/dL [25].**
**LV ejection fraction (EF) remains the major parameter for phenotyping, diagnosis,**

**prognosis and treatment decisions in HF. In the present study, EF of 50% was used as the cut-off to categorize the participants into two groups as group 1 – HF with EF ≤ 49% and group 2 -HF with EF ≥ 50%. As per the 2016- ESC Guidelines, EF of 40- 49% is categorized as HFmrEF. HFmrEF is an intermediate clinical entity between HFrEF and HFpEF, but more similar to HFrEF with regard to the high prevalence of AMI in these patients [7]. Patients with HFmrEF are similar to HFrEF in being younger males, increased systolic blood pressure and reduced LV and atrial dilatation. Also, HFmrEF are found to benefit from HFrEF treatment strategies [7]. In the present study, based on the above literature data, HFmrEF has been included in HFrEF.**

- The authors did not report inclusion / exclusion criteria, flow chart for patients enrolment, comorbidity status, anthropometric findings. Table 1 does not include the parameters mentioned above.

**Response: Figure 1 shows the flow chart indicating the recruitment of participants into the study along with patient characteristics. Table 1 shows the demographic data of the study participants**.

- Biomarkers evaluation and blood sampling. It remains unclear now and in which ways the sampling were performed.

**Response: The heart failure patients who were attending cardiology department were recruited. Based on EF as shown by ECHO they were categorized as group I or 2. Subsequently blood samples were drawn for analysis of biomarkers and microRNA. Serum and plasma were separated and stored at -80°C until analysis.**

- Did the authors discover other correlations between biomarkers and comorbidities, metabolic status, i.e. fasting glucose, HOMA-IR, lipid profile.

**Response: The correlations of cardiac biomarkers with lipids, MAGGIC score and HbA1c are shown in table 6**

Reviewer #2: With the aim of investigating levels of biomarkers in HF and to compare between HFrEF and HFpEF, the authors performed a cross-sectional study enrolling 270 patients. The authors are to be commended for the great effort. However, there are some points in need of further clarification.

**Thank you very much for analyzing our manuscript critically. I am grateful for the valuable suggestions and giving us the opportunity to answer to the queries. Indeed, we are thankful and most obliged for all the constructive comments. Thank you**

Specific points:

-Abstract:

Too much information about methods; please shorten it
Suddenly, the authors named in the result section controls. Please, add in the method.

**Responses: Abstract -methodology has been modified. The information about controls has been added in the methodology**

-Introduction: too long. Please, go straight to the main focus of the paper.

**Response: Introduction was edited and excess information has been removed.**

-Methods: to include only Hypertensive patients with HF and then add patients with DM could be considered as a mistake.

**Response: Initially the study was decided to be conducted in hypertensive patients only, since hypertension has not been studied in Indian population. Due to the sudden onset of COVID-19 pandemic, patient visit to the hospital was limited. Hence, with the discussion and approval of research advisory committee members, it was decided to include hypertensives who also had diabetes mellitus. Ethics approval was sought again for the change in the criteria of recruitment of patients.**

-Results: the result section should include only results. Please, delete all the information that are not results.

**Results section has been modified and only results have been included. Information related to methodology have been added in methods section.**

Please, add also comparison with regard to sex (M vs F) and age.

**Response: Tables 3 and 4 have been added to compare the groups according to gender and age respectively.**

-Discussion:
Too long and not focused on the main results. Pleas shorten it and make more focused on the main topic; in addition, add subparagraphs to improve readability.

**Response: Discussion has been edited and shortened as per the suggestions.**

To date, the role of biomarkers, and in particular the role of multimarkers, in HF can be considered in different stage (e.g., diagnosis, prognosis, management). Please comment using references (i.e., PMID 33673947 and 30832821)

**Response: I had the opportunity to go through both the manuscripts. Both are really good, with good presentation and lot of information, pointing to almost the same findings which I have put forth in the manuscript. I have them in manuscript also.**

Figures:
the figure are very low quality. Please increase.
Figure 1: please, miRNA and sST2 are too similar in color. Please change.

**Response: The color has been changed. New figure with better clarity is attached**

Consider to add in the text and in the discussion that one of the strengths of the present paper is that it has been performed in India (adding interesting additional data of patients whose ethnicity is classically not well investigated).

**Response: Strength of the study has been added.**

**The study is carried out in India, where the resource settings are limited. India as such is highly diverse in economy, food, physical habits, literacy rate across the various states. But the study was carried out in a tertiary care hospital in a Metropolitan city. So to major extent it takes into account of the various factors. A multi-marker study in a relatively large sample size comprising of both genders remains to be major strength of the study.**

Additional comment:
If the authors used AI for the preparation of this manuscript, please declare.

**Response: The authors declare that AI for not used in preparation of this manuscript.**

6. PLOS authors have the option to publish the peer review history of their article (what does this mean?). If published, this will include your full peer review and any attached files.

If you choose "no", your identity will remain anonymous but your review may still be made public.

**Do you want your identity to be public for this peer review?** For information about this choice, including consent withdrawal, please see our Privacy Policy.

Reviewer #1: No

Reviewer #2: **Yes:** Dr Andrea Salzano, MD, PhD
