# Supplementary material for: Association of microRNA-210-3p with NT-proBNP, sST2, and Galectin-3 in heart failure patients with preserved and reduced ejection fraction: A cross-sectional study
Source: PLoS One. 2025 Apr 3;20(4):e0320365. doi: 10.1371/journal.pone.0320365 (PMC11991677; doi:10.1371/journal.pone.0320365)
Supplement: Attachment — Submitted filename: Response to Reviewers.docx [file pone.0320365.s002.docx]

**The authors are grateful for all the suggestions raised by the reviewers and editors. Herewith we are attaching the responses to the queries. We have taken utmost care and responsibility in addressing the queries. Once again thank you very much.**

**Responses to Journal requirements:**	14-09-2024

1. Please ensure that your manuscript meets PLOS ONE's style requirements, including those for file naming.

**Response: The manuscript has been modified according to the PLOS ONE’s requirements.**

2. When completing the data availability statement of the submission form, you indicated that you will make your data available on acceptance. We strongly recommend all authors decide on a data sharing plan before acceptance, as the process can be lengthy and hold up publication timelines. Please note that, though access restrictions are acceptable now, your entire data will need to be made freely accessible if your manuscript is accepted for publication. This policy applies to all data except where public deposition would breach compliance with the protocol approved by your research ethics board. If you are unable to adhere to our open data policy, please kindly revise your statement to explain your reasoning and we will seek the editor's input on an exemption. Please be assured that, once you have provided your new statement, the assessment of your exemption will not hold up the peer review process.

**Response: The authors confirm that the data supporting the findings of this study are available within the article. Raw data were generated at the location of research, Sri Ramachandra Institute of Higher Education and Research, Chennai, India. No supplementary material is available with this research.**

3. Please remove your figures from within your manuscript file, leaving only the individual TIFF/EPS image files, uploaded separately. These will be automatically included in the reviewers’ PDF.

**Responses: Figures are removed from the manuscript file and uploaded separately as TIFF files.**

**Response to the reviewers comments:**
1. Is the manuscript technically sound, and do the data support the conclusions?

The manuscript must describe a technically sound piece of scientific research with data that supports the conclusions. Experiments must have been conducted rigorously, with appropriate controls, replication, and sample sizes. The conclusions must be drawn appropriately based on the data presented.

Reviewer #1: Yes

Reviewer #2: Partly

**Response: The manuscript has been edited with the responses to the reviewers, which we think could improve the content and presentation of the manuscript.**

 2. Has the statistical analysis been performed appropriately and rigorously?

Reviewer #1: Yes

Reviewer #2: No

**Response: Correlation studies and ROC curve have been modified. The information is added in the methodology, results, discussion and conclusion.**

 3. Have the authors made all data underlying the findings in their manuscript fully available?

The [PLOS Data policy](http://c05y1x9s.r.us-east-2.awstrack.me/L0/http:%2F%2Fwww.plosone.org%2Fstatic%2Fpolicies.action%23sharing/1/010f01914c85f8b1-285ed343-5c05-4831-b149-363e698f8162-000000/8c7RUrbmuPpxAYNk94QEcpjRIsM=171) requires authors to make all data underlying the findings described in their manuscript fully available without restriction, with rare exception (please refer to the Data Availability Statement in the manuscript PDF file). The data should be provided as part of the manuscript or its supporting information, or deposited to a public repository. For example, in addition to summary statistics, the data points behind means, medians and variance measures should be available. If there are restrictions on publicly sharing data—e.g. participant privacy or use of data from a third party—those must be specified.

Reviewer #1: Yes

Reviewer #2: Yes

4. Is the manuscript presented in an intelligible fashion and written in standard English?

PLOS ONE does not copyedit accepted manuscripts, so the language in submitted articles must be clear, correct, and unambiguous. Any typographical or grammatical errors should be corrected at revision, so please note any specific errors here.

Reviewer #1: Yes

Reviewer #2: Yes

5. Review Comments to the Author

Please use the space provided to explain your answers to the questions above. You may also include additional comments for the author, including concerns about dual publication, research ethics, or publication ethics. (Please upload your review as an attachment if it exceeds 20,000 characters)

Reviewer #1: The authors reported that NT-proBNP, sST2 and galectin-3 were significantly higher in HFrEF compared to that of HFpEF. Also, microRNA-210-3p showed significantly increased expression in HFpEF compared to controls. The findings seem to be interesting, however, I would like to make some comments.

1. Major concerns.
   - The lack of the novelty is the concern that I would like to express around the article, while data for miR-210-3p deserve to be published.

**Response: The study was intended to be conducted since NT-proBNP levels do not correlate with the severity of disease especially in cases of HFpEF. Hence, we researched to find out whether sST2 and Gal-3 could be able to diagnose those cases which were not identified by NT-proBNP. Also, microRNAs could serve as very early biomarkers of the disease. Like we do profile of certain biomarkers in case of liver or renal diseases, a profile of biomarkers in HF will reduce the chances of morbidity and mortality. Of late women tend to develop hypertension almost similar to that of men. Studies show that women respond poorly to antihypertensives compared to men. Hence, the chances of increasing cases of HFpEF is high in future. Keeping all these in mind, we proceeded with the study.**

- The authors did not validate the findings and the comparisons with widely used scores (MAGGIC, Barcelona-HF, etc.) were not performed

**Methodology: The Meta‐Analysis Global Group in Chronic Heart Failure (MAGGIC) mortality risk score was calculated in all the study participants. The MAGGIC score consists of thirteen clinical predictor variables such as age, sex, body mass index (BMI), systolic blood pressure (SBP), ejection fraction (EF), serum creatinine level, participant is a current smoker, has diabetes mellitus, and chronic obstructive pulmonary disease, belongs to New York Heart Association (NYHA) class, heart failure (HF) diagnosed for at least eighteen months, use of beta-blockers and angiotensin‐converting enzyme (ACE) inhibitors.** 

2. Minor concerns
- Structure of the paper is not logic. Section Results contains a paragraph for B-mode Echo, while it could replaced to the subsection Methods. Please, check and correct.

**Response: The following removed in results and added in methodology**

**The study participants were subjected to the transthoracic 2D doppler echocardiography using Phillips and GE Healthcare echocardiography with patients lying supine in the left lateral decubitus position. EF values in healthy individuals and in heart failure patients vary with ethnicity and type of ECHO instrument. According to the definition of European and United States guidelines, the normal EF is 52-72% in men and 54-74% in women [21]. According to Chengode, EF > 55% is considered to be normal. It can be 45%–54%, 30%–44% and < 30% in mild, moderate and severe LV dysfunction respectively [22].**

**Anthropometric characteristics such as body mass index (BMI) in Kg/m^2^ and waist to hip ratio (WHR) were measured as per the Asia-Pacific guidelines [23]. Systolic and diastolic blood pressures were measured using standard procedure and patients were identified as hypertensives based on the JNC7 blood pressure classification [24]. According to International Expert Committee, diabetes mellitus was diagnosed based on glycated hemoglobin (HbA1c) ≥6.5%, fasting plasma glucose ≥126 mg/dL and 2-hour plasma glucose or random plasma glucose ≥200 mg/dL [25].**

**LV ejection fraction (EF) remains the major parameter for phenotyping, diagnosis, prognosis and treatment decisions in HF.  In the present study, EF of 50% was used as the cut-off to categorize the participants into two groups as group 1 – HF with EF ≤ 49% and group 2 -HF with EF ≥ 50%. As per the 2016- ESC Guidelines, EF of 40- 49% is categorized as HFmrEF. HFmrEF is an intermediate clinical entity between HFrEF and HFpEF, but more similar to HFrEF with regard to the high prevalence of AMI in these patients [7]. Patients with HFmrEF are similar to HFrEF in being younger males, increased systolic blood pressure and reduced LV and atrial dilatation. Also, HFmrEF are found to benefit from HFrEF treatment strategies [7]. In the present study, based on the above literature data, HFmrEF has been included in HFrEF.**

- The authors did not report inclusion / exclusion criteria, flow chart for patients enrolment, comorbidity status, anthropometric findings. Table 1 does not include the parameters mentioned above.

**Response: Figure 1 shows the flow chart indicating the recruitment of participants into the study along with patient characteristics. Table 1 shows the demographic data of the study participants**.

- Biomarkers evaluation and blood sampling. It remains unclear now and in which ways the sampling were performed.

**Response: The heart failure patients who were attending cardiology department were recruited. Based on EF as shown by ECHO they were categorized as group I or 2. Subsequently blood samples were drawn for analysis of biomarkers and microRNA. Serum and plasma were separated and stored at -80°C until analysis.**

- Did the authors discover other correlations between biomarkers and comorbidities, metabolic status, i.e. fasting glucose, HOMA-IR, lipid profile.

**Response: The correlations of cardiac biomarkers with lipids, MAGGIC score and HbA1c are shown in table 6**

Reviewer #2: With the aim of investigating levels of biomarkers in HF and to compare between HFrEF and HFpEF, the authors performed a cross-sectional study enrolling 270 patients. The authors are to be commended for the great effort. However, there are some points in need of further clarification.

**Thank you very much for analyzing our manuscript critically. I am grateful for the valuable suggestions and giving us the opportunity to answer to the queries. Indeed, we are thankful and most obliged for all the constructive comments. Thank you**

Specific points:

-Abstract:
Too much information about methods; please shorten it
Suddenly, the authors named in the result section controls. Please, add in the method.

**Responses: Abstract -methodology has been modified. The information about controls has been added in the methodology**

-Introduction: too long. Please, go straight to the main focus of the paper.

**Response: Introduction was edited and excess information has been removed.**

-Methods: to include only Hypertensive patients with HF and then add patients with DM could be considered as a mistake.

**Response: Initially the study was decided to be conducted in hypertensive patients only, since hypertension has not been studied in Indian population. Due to the sudden onset of COVID-19 pandemic, patient visit to the hospital was limited. Hence, with the discussion and approval of research advisory committee members, it was decided to include hypertensives who also had diabetes mellitus**. **Ethics approval was sought again for the change in the criteria of recruitment of patients.**

-Results: the result section should include only results. Please, delete all the information that are not results.

**Results section has been modified and only results have been included. Information related to methodology have been added in methods section.**

Please, add also comparison with regard to sex (M vs F) and age.

**Response: Tables 3 and 4 have been added to compare the groups according to gender and age respectively.**

-Discussion:
Too long and not focused on the main results. Pleas shorten it and make more focused on the main topic; in addition, add subparagraphs to improve readability.

**Response: Discussion has been edited and shortened as per the suggestions.**

To date, the role of biomarkers, and in particular the role of multimarkers, in HF can be considered in different stage (e.g., diagnosis, prognosis, management). Please comment using references (i.e., PMID 33673947 and 30832821)

**Response: I had the opportunity to go through both the manuscripts. Both are really good, with good presentation and lot of information, pointing to almost the same findings which I have put forth in the manuscript. I have them in manuscript also.**

Figures:
the figure are very low quality. Please increase.
Figure 1: please, miRNA and sST2 are too similar in color. Please change.

**Response: The color has been changed. New figure with better clarity is attached**

Consider to add in the text and in the discussion that one of the strengths of the present paper is that it has been performed in India (adding interesting additional data of patients whose ethnicity is classically not well investigated).

**Response: Strength of the study has been added.**

**The study is carried out in India, where the resource settings are limited. India as such is highly diverse in economy, food, physical habits, literacy rate across the various states. But the study was carried out in a tertiary care hospital in a Metropolitan city. So to major extent it takes into account of the various factors. A multi-marker study in a relatively large sample size comprising of both genders remains to be major strength of the study.**

Additional comment:
If the authors used AI for the preparation of this manuscript, please declare.

**Response: The authors declare that AI for not used in preparation of this manuscript.**

6. PLOS authors have the option to publish the peer review history of their article ([what does this mean?](https://c05y1x9s.r.us-east-2.awstrack.me/L0/https:%2F%2Fjournals.plos.org%2Fplosone%2Fs%2Feditorial-and-peer-review-process%23loc-peer-review-history/1/010f01914c85f8b1-285ed343-5c05-4831-b149-363e698f8162-000000/IL2MfmBrUMAw97m3-TURSHkzcow=171)). If published, this will include your full peer review and any attached files.

If you choose “no”, your identity will remain anonymous but your review may still be made public.

**Do you want your identity to be public for this peer review?** For information about this choice, including consent withdrawal, please see our [Privacy Policy](https://c05y1x9s.r.us-east-2.awstrack.me/L0/https:%2F%2Fwww.plos.org%2Fprivacy-policy/1/010f01914c85f8b1-285ed343-5c05-4831-b149-363e698f8162-000000/4PEwd7RbRAWokyjc0ShtK7GTHCU=171).

 Reviewer #1: No

Reviewer #2: **Yes:** Dr Andrea Salzano, MD, PhD
