# Supplementary material for: Association of microRNA-210-3p with NT-proBNP, sST2, and Galectin-3 in heart failure patients with preserved and reduced ejection fraction: A cross-sectional study
Source: PLoS One. 2025 Apr 3;20(4):e0320365. doi: 10.1371/journal.pone.0320365 (PMC11991677; doi:10.1371/journal.pone.0320365)
Supplement: Attachment — Submitted filename: Reviewer 3.docx [file pone.0320365.s003.docx]

**Comments to the authors**

**Abstract**

It is filled with unnecessary information, make it shorter.

**Introduction**

It does not focus to the main subject of the paper (this introduction includes already known information, focus mostly on microRNAs)

**Methods**

The clinical group should be regarded as a whole, with the two subgroups with and without DM

**Results**

This section is very slopy and not very clear, please stick only to the data, not its elaboration or clarification. Each time that you have statistically significant results, emphasize it with “p” value, where there is no statistical significance do not repeat it again, it is already stated in the Tables.

**Discussion**

This part should be rewritten completely, since it contains too much data and information and it is not clear for the readers. Also, the authors should include the newest guidelines, whether ESC or AHA, since mentioning 2013, or 2017 guideline is not of relevance. Still there is much said about markers of fibrosis and inflammation, including BNP. Therefore, the authors should emphasize microRNA and to mention other biomarkers just to make the comparison with microRNA, which should be the most important and the key point of the study (see the references PMID: 37734333 and PMID: 32295985)

**Figures and Tables**

Please, make them as clear as possible for better reading and understanding
